# Realizing Inclusion and Systemic Equity in Medicine: Upstanding in the Medical Workplace (RISE UP)—an Antibias Curriculum

**DOI:** 10.15766/mep_2374-8265.11233

**Published:** 2022-04-06

**Authors:** Sarah J. Calardo, Maybelle Kou, Courtney Port, Natalie McKnight, B. Elise Switzer, Kamilah Halmon, Kenia Lobo, Ghofrane Benghanem, Patricia W. Seo-Mayer

**Affiliations:** 1 Chief Resident, Pediatrics, Inova Children's Hospital; 2 Emergency Physician, Inova Fairfax Hospital; Professor of Medical Education, University of Virginia School of Medicine; Director of Simulation and Innovative Learning for Graduate Medical Education, Inova Fairfax Medical Campus/Inova Children's Hospital; 3 Hospitalist, Pediatrics, Inova Children's Hospital; Director of Quality and Safety Curriculum, Inova Pediatric Residency Program, Inova Children's Hospital; 4 Hospitalist, Pediatrics, Inova Children's Hospital; Assistant Dean for Faculty, University of Virginia School of Medicine, Inova Campus; 5 Hospitalist, Pediatrics, Inova Children's Hospital; 6 Hospitalist, Pediatrics, and Pediatric Hospital Medicine Fellowship Director, Inova Children's Hospital; 7 Second-Year Pediatric Resident, Inova Children's Hospital; 8 Emergency Medicine Physician, Inova Fairfax Hospital; Assistant Professor, University of Virginia School of Medicine; 9 Nephrologist, Pediatrics, Pediatric Specialists of Virginia and Inova Children's Hospital; Associate Program Director, Inova Pediatric Residency Program, Inova Children's Hospital

**Keywords:** Anti-racism, Bias, Small-Group Debriefing, Diversity, Inclusion, Health Equity

## Abstract

**Introduction:**

Racism is a public health threat, and racist behaviors adversely affect clinicians in addition to patients. Medical trainees commonly experience racism and bias. More than half of pediatric residents at a single institution reported experiencing or witnessing discriminatory behavior at work; only 50% reported receiving training on implicit bias, delivering difficult feedback, or peer support. Our multispecialty team created Realizing Inclusion and Systemic Equity in Medicine: Upstanding in the Medical Workplace (RISE UP), an antibias, anti-racism communication curriculum composed of three hybrid (virtual and in-person) workshops.

**Methods:**

During the pediatric resident workshops, we introduced tools for addressing bias, presented video simulations, and led small-group debriefings with guided role-play. We also reviewed escalation pathways, reporting methods, and support systems. Residents completed an evaluation before and after each workshop to assess the curriculum's efficacy.

**Results:**

Thirty-nine residents participated in RISE UP, with 20 attending all three workshops. Ninety-six percent of participants indicated they would recommend the workshops to colleagues. After the third workshop, 92% reported having tools to respond to bias, and 85% reported knowing how to escalate concerns regarding discriminatory behavior. Chief residents were most frequently identified as sources of resident support when encountering discriminatory behavior.

**Discussion:**

This curriculum was successful in developing and strengthening residents’ responses to discrimination, including upstander support. The curriculum is adaptable for virtual, in-person, and hybrid settings, allowing for flexibility. Establishing institutional support, promoting faculty development, and creating and disseminating escalation pathways are critical to addressing racism in health care.

## Educational Objectives

By the end of this activity, learners will be able to:

1. Utilize the STR (stop, talk, roll) and the STEP (step back, think through biases, evaluate emotions, prevent patient impact) tools to respond to bias or racism in the health care setting.
2. Explore and practice individual responses to discrimination in the health care setting during guided role-play.
3. Apply the DARE (discover, actively listen, recognize, educate) tool when experiencing or witnessing discrimination in the health care setting.

## Introduction

Racism is a public health threat,^1^ and clinicians have a responsibility to mitigate the effects of racism on patients as well as trainees and members of the health care workforce.^2–4^ Bias directed against patients has epigenetic and physiologic impacts^5,6^ and adversely affects medical decision-making, access to medical procedures and treatment, and patient outcomes.^7–9^ Discrimination directed towards health care providers is also common and causes exhaustion, stress, and burnout.^3,10,11^ Medical trainees are known to experience and witness racial bias in the workplace,^12^ which threatens the building of the diverse workforce critical to providing equitable care and reducing health disparities.^13,14^ Health care providers need to be trained to talk about race and racism, and leaders in health care need to model these nuanced discussions at all levels of the system.^15^

When we designed this resource, there were many calls for institutional support for anti-racism action but few published protocols.^16,17^ Published curricula, including those in *MedEdPORTAL*, cover various facets of anti-racism education, including reducing stigmatizing language,^18^ addressing microaggressions,^19,20^ teaching intersectionality,^21^ and generating professional and leadership development on promoting open dialogue on anti-racism topics.^22^ At the time of our literature review, no publications described simulation and debrief-based methods for anti-racism instruction that had been specifically developed for virtual or hybrid settings. There was also a paucity of literature detailing how comfortable medical students and residents felt discussing and confronting racism in a clinical setting. The 2019 American Academy of Pediatrics Statement on the Impact of Racism on Child and Adolescent Health recommended that simulation opportunities and active learning strategies should be integrated in pediatric residency training to adequately prepare residents to take care of a diverse patient population and support diversity in the pediatric workforce.^2^

The Realizing Inclusion and Systemic Equity in Medicine: Upstanding in the Medical Workplace (RISE UP) Consortium at Inova Children's Hospital identified the need for directed education on confronting racism and bias in clinical settings. With the deaths of George Floyd, Breonna Taylor, Ahmaud Arbery, and others galvanizing the need to implement such training and policy making across the health system, we sought to design a curriculum to address provider training while still adhering to pandemic-related restrictions.

We began by administering a needs assessment survey as part of the curriculum evaluation in 2018. When surveyed anonymously, more than half of pediatric residents (57%, *n* = 17) in our program reported experiencing or witnessing discriminatory behavior in clinical settings. Only 50% (*n* = 15) had previously received training in implicit bias, delivering difficult feedback, or peer support. Furthermore, only 16% (*n* = 5) had received training in all three categories, and 20% (*n* = 6) had not received training in any of them. Of senior-level physicians, administrative leadership, and nursing leadership surveyed, 88% (*n* = 15) had witnessed racism or bias in the health care environment, and 53% (*n* = 9) had personally experienced it. Informal interviews and focus groups of pediatric residents, faculty, and nursing leadership revealed a lack of comfort and confidence in how to address instances of racism and discrimination. Learners identified the desire for anti-racism training—specifically, being taught tools and words to confront perpetrators of racism and discrimination in clinical settings—and the need for an escalation pathway that would be supported by the system.

This assessment survey demonstrated that acts of bias and racism from peers, superiors, and patients were affecting our trainees, faculty, and staff. Adequate support of trainees required faculty and leadership development on how to respond to bias and racism and how to provide debriefing and support as well as emphasizing the need for a defined escalation and support pathway.

From the results of the needs assessment, we outlined the objectives of a time-bound educational and virtual curriculum that would utilize simulation and debriefing. We hypothesized that using communication tools and debriefing methods through simulation would improve resident comfort in discussing bias and racism and that we could implement pathways to respond to both at the individual and institutional levels. Using three antibias communication tools—two of which were internally developed—we created an innovative virtual simulation curriculum to rapidly deploy this education across our health system. Our objective was to create an antibias training curriculum—RISE UP—that increased the percentage of residents who felt comfortable discussing racism in the clinical setting, had the tools to respond to discrimination, and were aware of institutional support structures to 90% within 6 months.

## Methods

### Curriculum Design and Faculty Training

We assembled a multispecialty team—the RISE UP Consortium—representing pediatrics, emergency medicine, and trauma surgery. Members were chosen for their expertise in trauma-informed care, simulation education, debriefing, and quality and safety. The group consisted of program leadership, faculty, licensed clinical social workers, and residents.

We designed the RISE UP curriculum to teach health care providers the skills to address and respond to bias and racism in clinical settings. The first installment was developed through biweekly meetings and iterative review from August to October 2020. We piloted the resulting curriculum, featuring two modules, with a group of regional pediatric emergency medicine fellows in November 2020 and then presented the full curriculum, in three individual workshops, to pediatric residents from November 2020 to February 2021. We next presented the RISE UP curriculum to our institution's senior physician and nursing leadership and the larger GME community in an effort to create system-level change.

Our residency program trained 13 categorical pediatric residents per year at a children's hospital within an independent academic medical center in northern Virginia (a total of 39 pediatric residents). We scheduled the resident workshops into the program's protected academic half-day education to ensure high attendance and participation. The workshops were designed for an ideal ratio of two facilitators for each small group of six to eight participants. The facilitators for the resident workshops included pediatric hospitalists and emergency physicians (some with simulation expertise), a pediatric nephrologist, and licensed clinical social workers. We trained faculty to facilitate and debrief the workshops over two separate 1-hour sessions.

We created three video simulation scenarios based upon lived experiences of the consortium members, who represented a racially, ethnically, and religiously diverse group. The video scenarios (1–2 minutes) featured standardized patient actors as well as pediatric residents and faculty and were filmed by professional media services at our institution. The scenarios were entitled The Racist Patient, The Racist Provider, and The Racist Consultant (Appendices A–C), and we included video transcripts in Appendix H.

### Curriculum Tool Kit

With the intention of sharing this curriculum, we developed this RISE UP workshop facilitator's tool kit through an iterative process. This tool kit included the facilitator guide (Appendix E), the workshop slide deck (Appendix D) with video vignettes, communication and peer support tools, and quick response (QR) codes linking to the learner pre- and postworkshop surveys (Appendices F and G). It offered a ready-to-use anti-racism curriculum that could be adapted and presented in virtual, hybrid, or in-person settings at users’ local institutions or departments. Materials needed to implement the curriculum included a debriefing room with movable furniture for small groups (if in person) and audiovisual capabilities, including a computer, projector, slide advancer, and Wi-Fi.

The tool kit outlined different curriculum format options based on timing and availability, with an option for three 60-minute workshops each focusing on a single video simulation (with the recommended order noted by the numerically ordered workshops) and another option for a two-part series consisting of a 90-minute, two-video workshop followed by a subsequent 60-minute, final-video-focused workshop.

### Curriculum Delivery

We implemented the RISE UP curriculum in three 60-minute workshops. There was no prerequisite reading designated as preparation for the curriculum, although we recommended residents complete any two of Project Implicit's Implicit Association Tests^23^ in an effort to highlight the need for the curriculum's topics and allow for self-reflection.

We asked learners to complete a preworkshop survey (Appendix F) investigating their previous experiences with bias and racism as well as how prepared they felt to address or cope with those situations. We instructed participants to keep an open mind and remain on camera throughout the workshop.

#### Workshop 1

We presented a background on the history of systemic racism in medicine and the evolution of its impact on patients and providers. We gave a prebrief that reviewed debriefing methods and tips to increase virtual engagement and introduced participants to the concept of a brave space,^24^ acknowledging the difficult topic and providing an option to step in and out of challenging conversations for those who might relive experienced racial trauma. We then introduced the first antibias communication tool, STR (stop, talk, roll), developed by the Georgetown School of Medicine Office of Diversity and Equity.^25^ Participants watched The Racist Patient (Appendix A) and entered breakout groups for a facilitated small-group debrief of the video simulation and a role-play of responses to racist patients and families. We reconvened as a large group to introduce our DARE (discover, actively listen, recognize, educate) tool for use as a peer supporter and discussed a newly designed institutional escalation pathway for bias events and sources of support.

#### Workshop 2

We introduced the STEP (step back, think through biases, evaluate emotions, prevent patient impact) tool, our novel framework for addressing personal biases. We then showed The Racist Provider (Appendix B) video. Learners debriefed in small breakout groups and completed a role-play focused on addressing personal biases and their effects on patient care, as well as responding effectively to racist or biased comments from peers.

#### Workshop 3

We began by showing The Racist Consultant (Appendix C), which was followed by small-group debriefing and further role-play. Participants practiced using the three communication tools they had been taught in the preceding workshops. Participants then returned to the concluding large-group session where we reviewed institutional escalation pathways, methods of reporting, and support systems for those impacted by racism in the workplace.

### Evaluation

We administered a postworkshop survey (Appendix G) to participants after each of the three workshops via a QR code. The survey investigated the residents’ level of ease in discussing racism; their improvement in the recognition of racism in the workplace; their knowledge of the tools to respond, escalate, and/or report; and their likelihood of using the tools when racism and bias occurred. Residents responded to each question using a 5-point Likert scale (1 = *strongly disagree,* 5 = *strongly agree*).

We collected data by electronic survey prior to the first workshop, after each of the three workshops, and 3 months after the first workshop (6 months from the preworkshop survey). We used Fisher's exact test to compare changes in the percentage of participants reporting comfort discussing bias, including having the tools to respond and knowledge of escalation pathways when encountering bias events. We also asked all residents the same questions from the needs assessment on the residency survey to assess any changes in residents’ perceptions of formal training in topics within the RISE UP curriculum.

## Results

**Figure 1.**
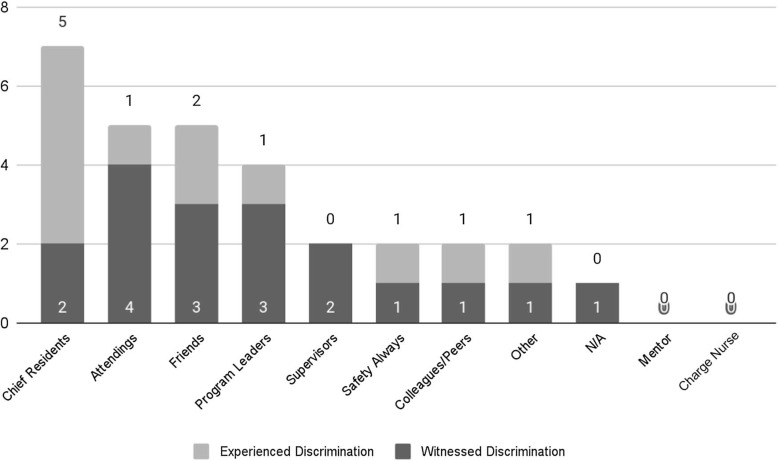
The sources of support for pediatric residents who reported experiencing and/or witnessing discrimination bias in the workplace (*N* = 30).

All 39 residents completed at least two of the three workshops, with 20 (51%) completing all three workshops. Workshop survey response rates ranged from 36% to 51%. On the preworkshop survey, chief residents were most frequently identified as sources of resident support for witnessing or experiencing discriminatory behavior, followed by program leadership, attendings, and friends (Figure 1).

RISE UP curricular feedback was positive, with 96% of residents (*n* = 25) stating they would recommend the workshop series and 92% (*n* = 24) agreeing they were better prepared to approach encounters with racism and discrimination in the workplace as a result of the curriculum. We did not succeed in increasing resident ease in discussing bias with colleagues, but our baseline presurvey data showed that 93% of residents (*n* = 28) had already reported ease in this area.

Postworkshop survey comments from residents included “Group talk helped me to know my unrecognized biased ideas” and showed that residents were interested in “more strategies on how to stop my biases at the start of [patient] encounters.” Suggestions for improvement included “More time for small group discussion,” as well as requests to tackle additional biases and to host the workshops outside of the residency academic half-day to allow reflection on the conversations before moving on to the subsequent traditional academic lectures.

### Knowledge and Tools

**Figure 2.**
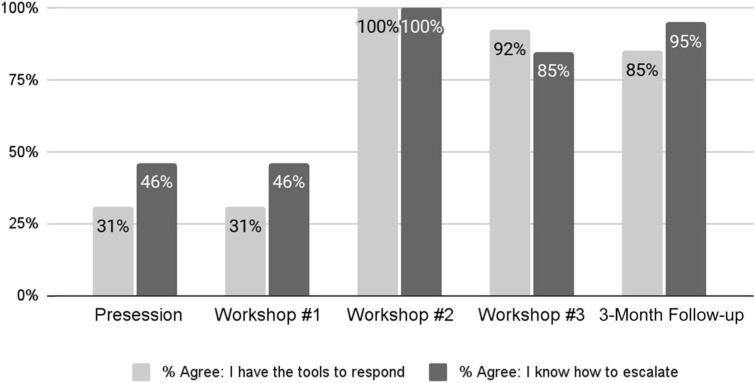
The percentage of pediatric residents who strongly agreed or agreed they had the tools to respond to, and knew how to escalate, discriminatory behavior in the workplace from presurvey to the 3-months follow-up postworkshop survey.

By the end of the third workshop, 92% of respondents (*n* = 13) strongly agreed or agreed they had the tools to respond to bias, compared to only 31% (*n* = 9) prior to the workshops (*p* < .001; Figure 2). Three months later (6 months after the presurvey), these improvements were maintained, with 85% of respondents (*n* = 17) reporting they strongly agreed or agreed they had the tools to respond to bias and 95% (*n* = 19) indicating they knew how to escalate discriminatory behavior, compared to 46% (*n* = 14) prior to the curriculum (*p* = .04; Figure 2).

On the 3-month follow-up survey, approximately two-thirds of respondents agreed or strongly agreed they were likely to use the three tools taught in the RISE UP curriculum in the future when encountering bias events in the workplace (STR: 69%, *n* = 9; STEP: 62%, *n* = 8; DARE: 62%, *n* = 8), while the remaining respondents reported they were undecided (*neither agree nor disagree*). None responded they were unlikely to use the tools (*disagree* or *strongly disagree*).

### Resident Perceptions of Training

**Figure 3.**
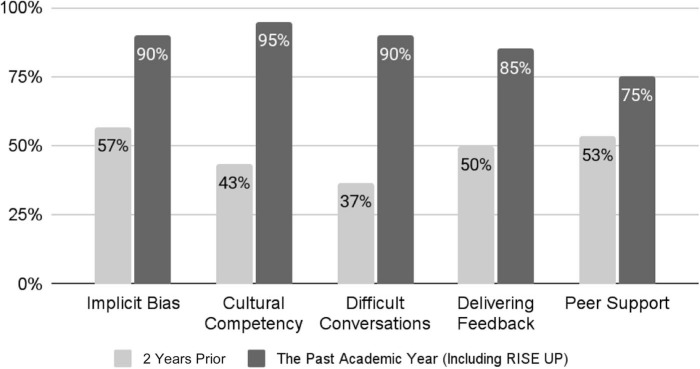
The percentages of pediatric residents who reported having formal training in topics including implicit bias, cultural competency, difficult conversations, delivery of feedback, and peer support pre- (*N* = 30) and postimplementation (*N* = 20) of the Realizing Inclusion and Systemic Equity in Medicine: Upstanding in the Medical Workplace (RISE UP) curriculum.

Following implementation of the RISE UP curriculum, 75%–95% of residents reported completion of formal training in various topics, including implicit bias, difficult conversations, and peer support, compared to only 37%–57% who had responded to the needs assessment survey 2 years prior (Figure 3).

## Discussion

We developed RISE UP to address the prevalence of witnessed and experienced bias in health care and the lack of training for confronting it. We found our curriculum was effective in teaching anti-racism and antibias techniques to pediatric residents via three workshops. Prerecorded simulated video scenarios depicting racist patients and biased colleagues provided learners the opportunity to witness, debrief, and role-play responses in a brave space. The hybrid format allowed for implementation in multiple settings, a necessity during the COVID-19 pandemic era of social distancing.

We reached our aim of increasing the percentage of residents who reported having the tools to respond to discrimination and knowing how to escalate biased events to 90% by the second of our three RISE UP workshops. Although these improvements were not immediate, they were sustained 3 months later at 85% and 90%, respectively. Since improvement was not seen after just one workshop, we theorize that multiple workshops are needed. We did not see an improvement in the percentage of residents who felt comfortable discussing racism in the clinical setting, likely because a high percentage (93%) had already reported being comfortable prior to the first RISE UP workshop. This high degree of comfort was likely multifactorial and may have included increased willingness and comfort among younger generations regarding discussing racism, our program's culture of encouraging open and honest discussions, and previous conversations between our residents and leadership on systemic racism that had taken place prior to the first RISE UP workshop.

RISE UP also trained residents to be peer supporters and active upstanders, rather than bystanders, when confronting bias and taught them how to report and escalate. Through the preworkshop survey results, we saw that training chief residents in responding to bias, escalation policies, cultural competency, and peer support was critical. The curriculum's implementation within the larger GME community acted as a catalyst to address these concerns at a system level. This resulted in ongoing development of institutional escalation and provider protection policies, which were key to supporting learners and clinicians. Modifications of this curriculum have been presented at multiple national meetings, and we plan to continue to use feedback to improve upon our rapid prototype curriculum.

### Limitations and Challenges

Limitations included a small sample size and number of participants. There was a significant amount of time required (two separate 1-hour sessions) to train our faculty to facilitate and debrief the workshops. We worked to overcome this for future workshops by designing the facilitator guide to assist novel facilitators in leading small-group debriefs. The curriculum's success was also dependent on a willingness to discuss bias, as well as buy-in from leadership and administration in terms of adoption of an institutional antidiscrimination policy, development of a universal escalation system, and enforcement of policy ideals. Challenges throughout the curriculum noted by facilitators included gaining participants’ trust when stepping into the brave space during the small-group debriefs and eliciting willing participants to role-play uncomfortable scenarios. In response to these challenges, we developed specific strategies for facilitators to manage difficult conversations with participants who might dominate conversations or be reluctant to contribute to the debriefs. Importantly, participating in these role-play scenarios and discussions may trigger previously experienced racial trauma among participants and facilitators, so facilitators should be prepared to support those individuals and each other during and after the workshops.

### Future Plans

Future plans for the RISE UP curriculum include the expansion of its focus to address other forms of bias and to target learners at different stages in their careers. RISE UP has become a part of our institution's GME orientation for incoming trainees. We recently modified this workshop to discuss microaggressions and have plans to expand it to address anti-LGBTQ bias.

## Appendices


Video 1 - The Racist Patient.mp4Video 2 - The Racist Provider.mp4Video 3 - The Racist Consultant.mp4Workshop Slides.pptxFacilitator Guide.pptxPreworkshop Survey.docxPostworkshop Survey.docxSimulation Video Transcripts.docx
*All appendices are peer reviewed as integral parts of the Original Publication.*
