# Supplementary material for: Realizing Inclusion and Systemic Equity in Medicine: Upstanding in the Medical Workplace (RISE UP)—an Antibias Curriculum
Source: MedEdPORTAL. 2022 Apr 6;18:11233. doi: 10.15766/mep_2374-8265.11233 (PMC8983799; doi:10.15766/mep_2374-8265.11233)
Supplement: Supplementary file 1 — Video 1 - The Racist Patient.mp4Video 2 - The Racist Provider.mp4Video 3 - The Racist Consultant.mp4Workshop Slides.pptxFacilitator Guide.pptxPreworkshop Survey.docxPostworkshop Survey.docxSimulation Video Transcripts.docx [file mep_2374-8265.11233-s001.zip › D. Workshop Slides.pptx]

## Slide 1
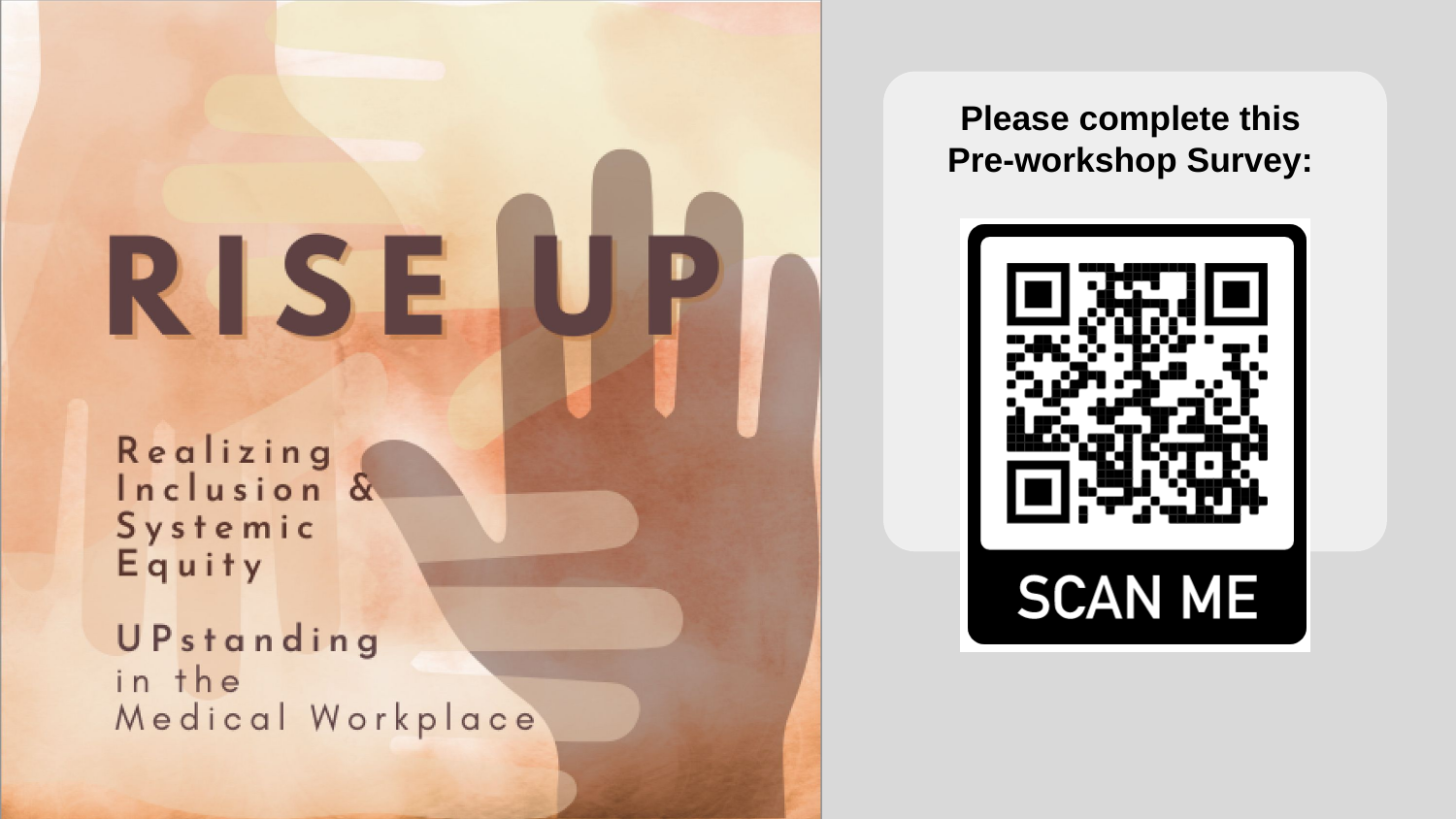

Please complete this
Pre-workshop Survey:
QR CODE***

## Slide 2
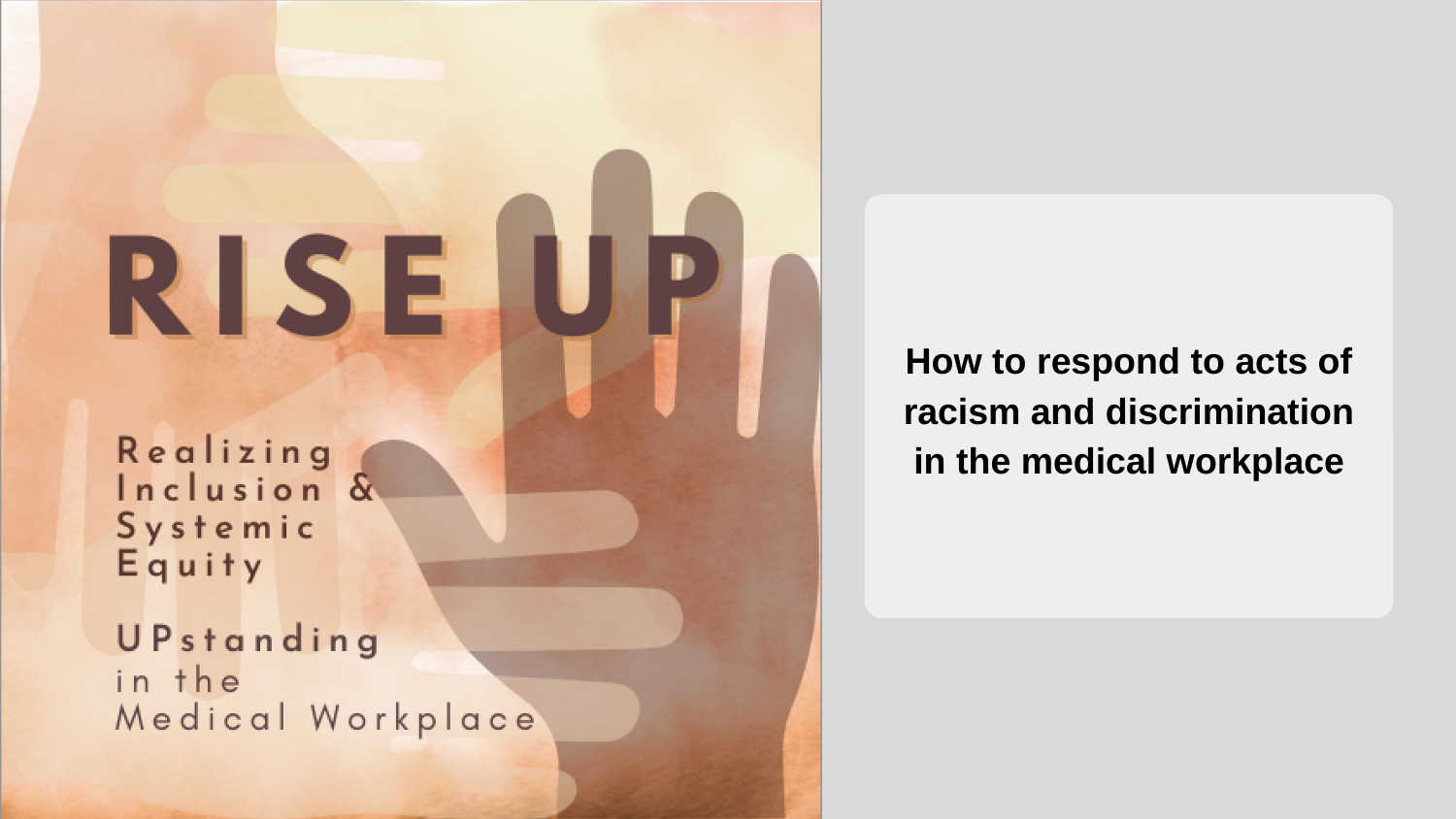

How to respond to acts of racism and discrimination in the medical workplace

## Slide 3
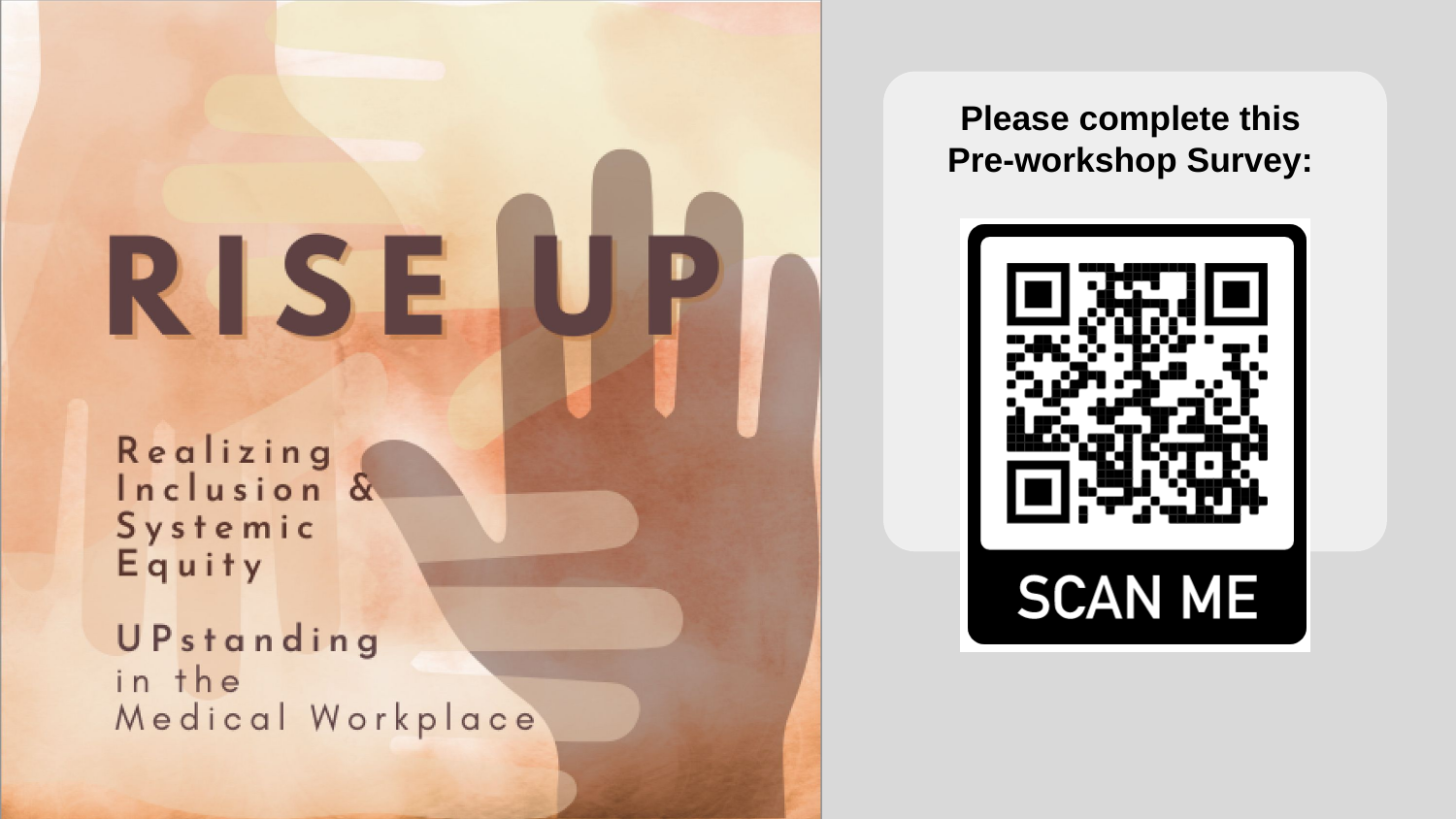

Please complete this
Pre-workshop Survey:
QR CODE***

## Slide 4
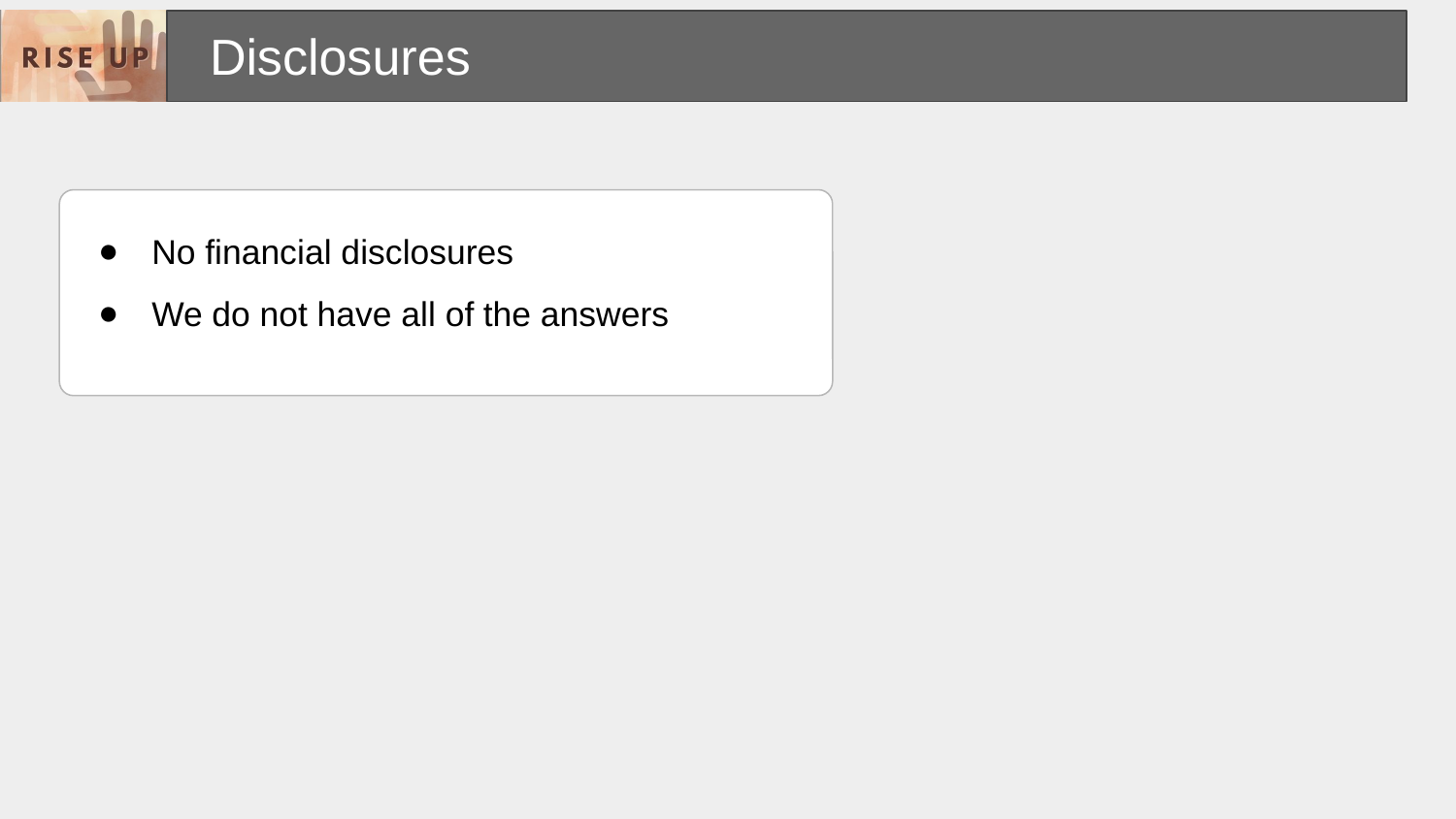

# Disclosures
No financial disclosures
We do not have all of the answers

## Slide 5
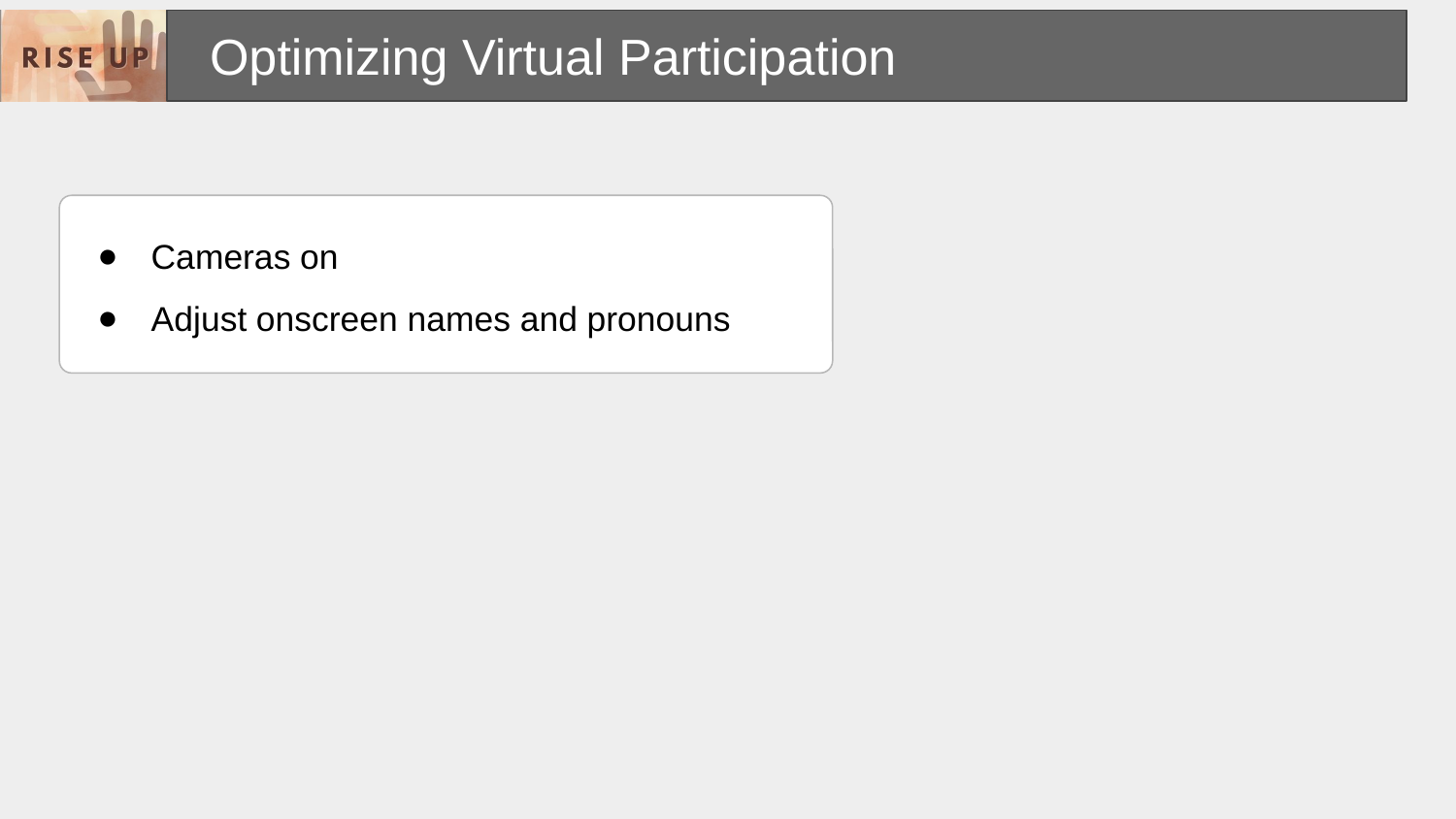

# Optimizing Virtual Participation
Cameras on
Adjust onscreen names and pronouns

## Slide 6
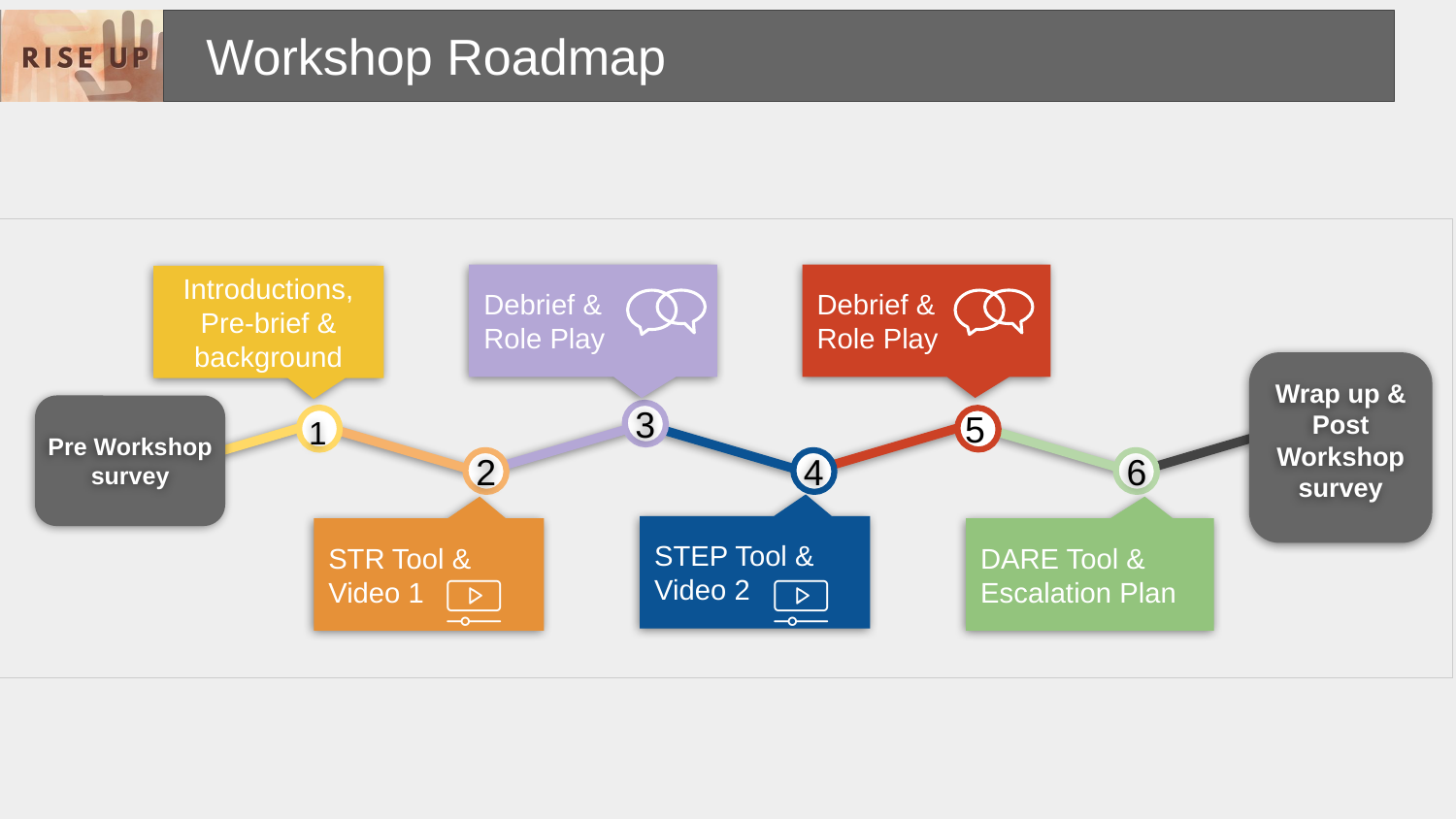

# Workshop Roadmap
Introductions, Pre-brief & background
Debrief &
Role Play
Debrief &
Role Play
Wrap up & Post Workshop survey
3
5
Pre Workshop survey
1
4
6
2
STEP Tool & Video 2
STR Tool & Video 1
DARE Tool & Escalation Plan

## Slide 7
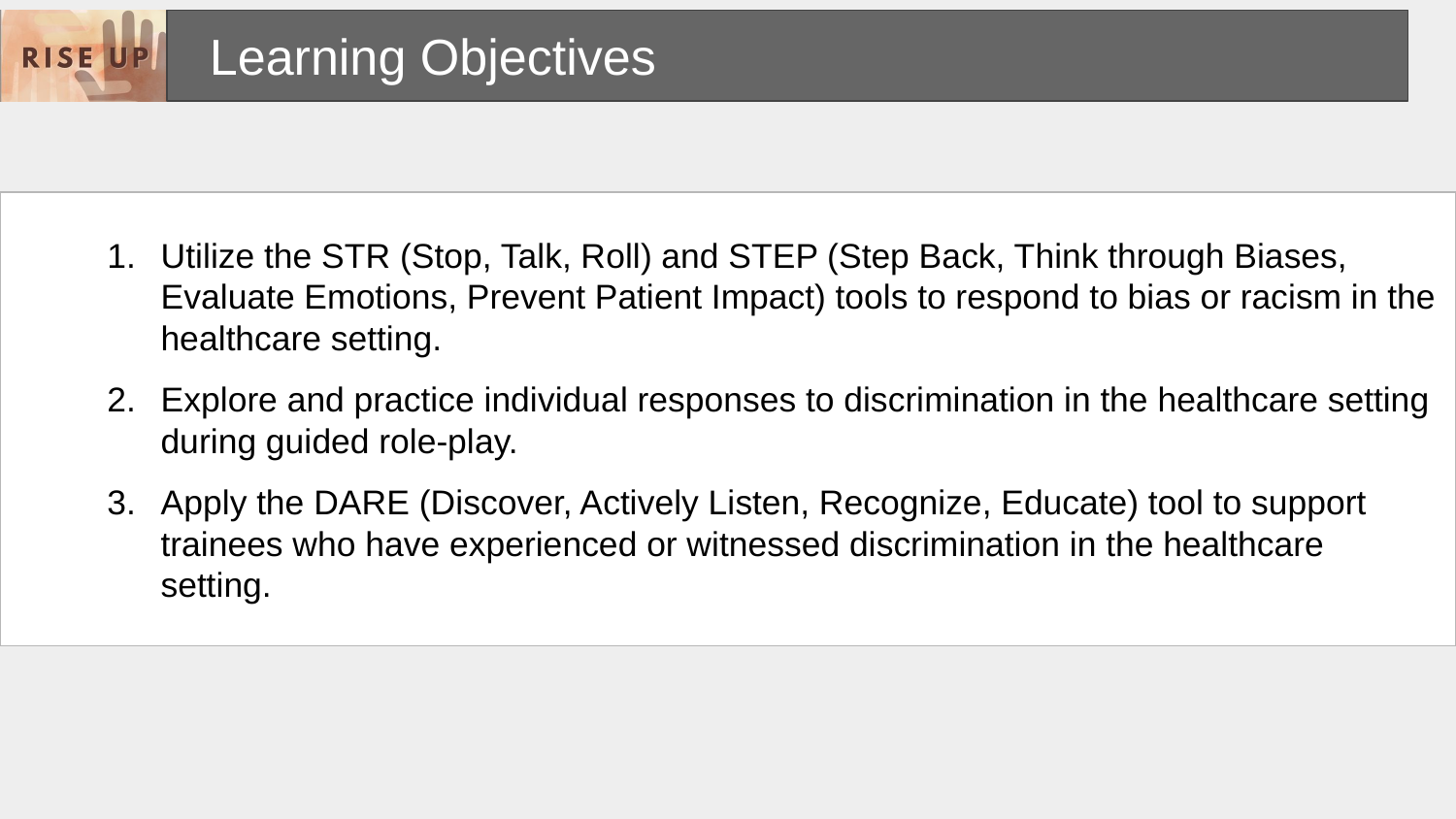

# Learning Objectives
Utilize the STR (Stop, Talk, Roll) and STEP (Step Back, Think through Biases, Evaluate Emotions, Prevent Patient Impact) tools to respond to bias or racism in the healthcare setting.
Explore and practice individual responses to discrimination in the healthcare setting during guided role-play.
Apply the DARE (Discover, Actively Listen, Recognize, Educate) tool to support trainees who have experienced or witnessed discrimination in the healthcare setting.

## Slide 8
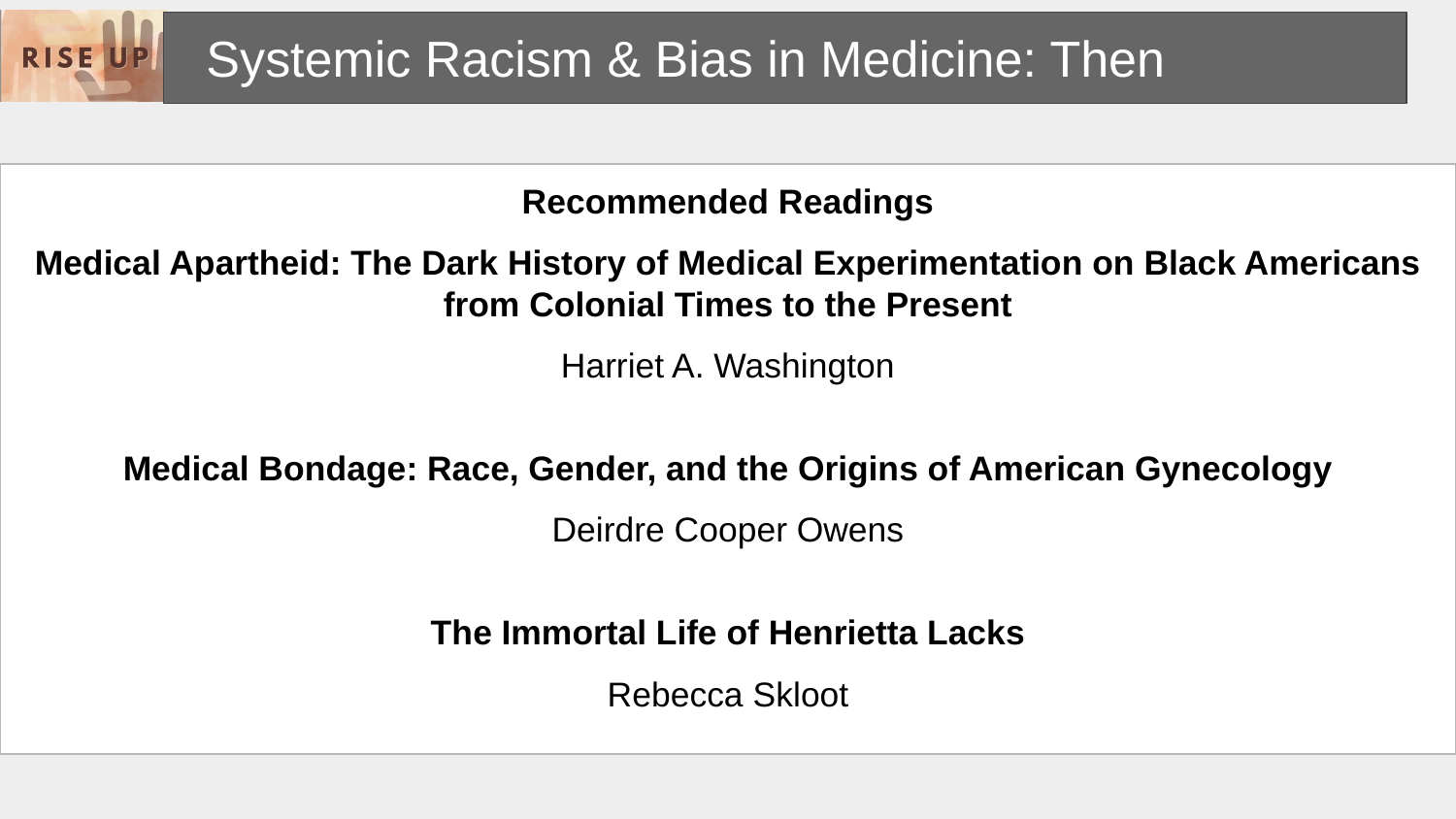

# Systemic Racism & Bias in Medicine: Then
Recommended Readings
Medical Apartheid: The Dark History of Medical Experimentation on Black Americans from Colonial Times to the Present
Harriet A. Washington
Medical Bondage: Race, Gender, and the Origins of American Gynecology
Deirdre Cooper Owens
The Immortal Life of Henrietta Lacks
Rebecca Skloot

## Slide 9
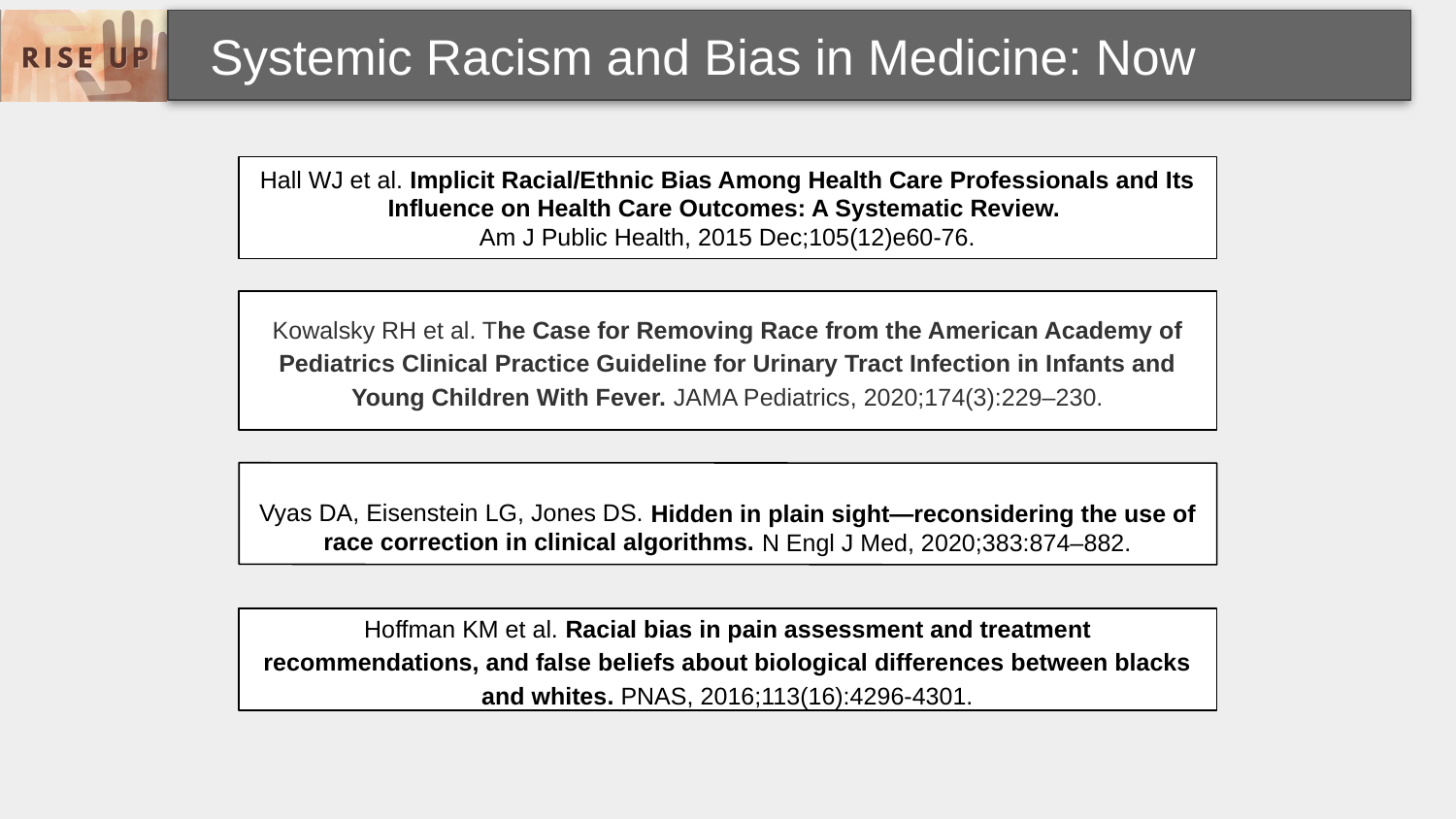

# Systemic Racism and Bias in Medicine: Now
Hall WJ et al. Implicit Racial/Ethnic Bias Among Health Care Professionals and Its Influence on Health Care Outcomes: A Systematic Review.
Am J Public Health, 2015 Dec;105(12)e60-76.
Kowalsky RH et al. The Case for Removing Race from the American Academy of Pediatrics Clinical Practice Guideline for Urinary Tract Infection in Infants and Young Children With Fever. JAMA Pediatrics, 2020;174(3):229–230.
Vyas DA, Eisenstein LG, Jones DS. Hidden in plain sight—reconsidering the use of race correction in clinical algorithms. N Engl J Med, 2020;383:874–882.
Hoffman KM et al. Racial bias in pain assessment and treatment recommendations, and false beliefs about biological differences between blacks and whites. PNAS, 2016;113(16):4296-4301.

## Slide 10
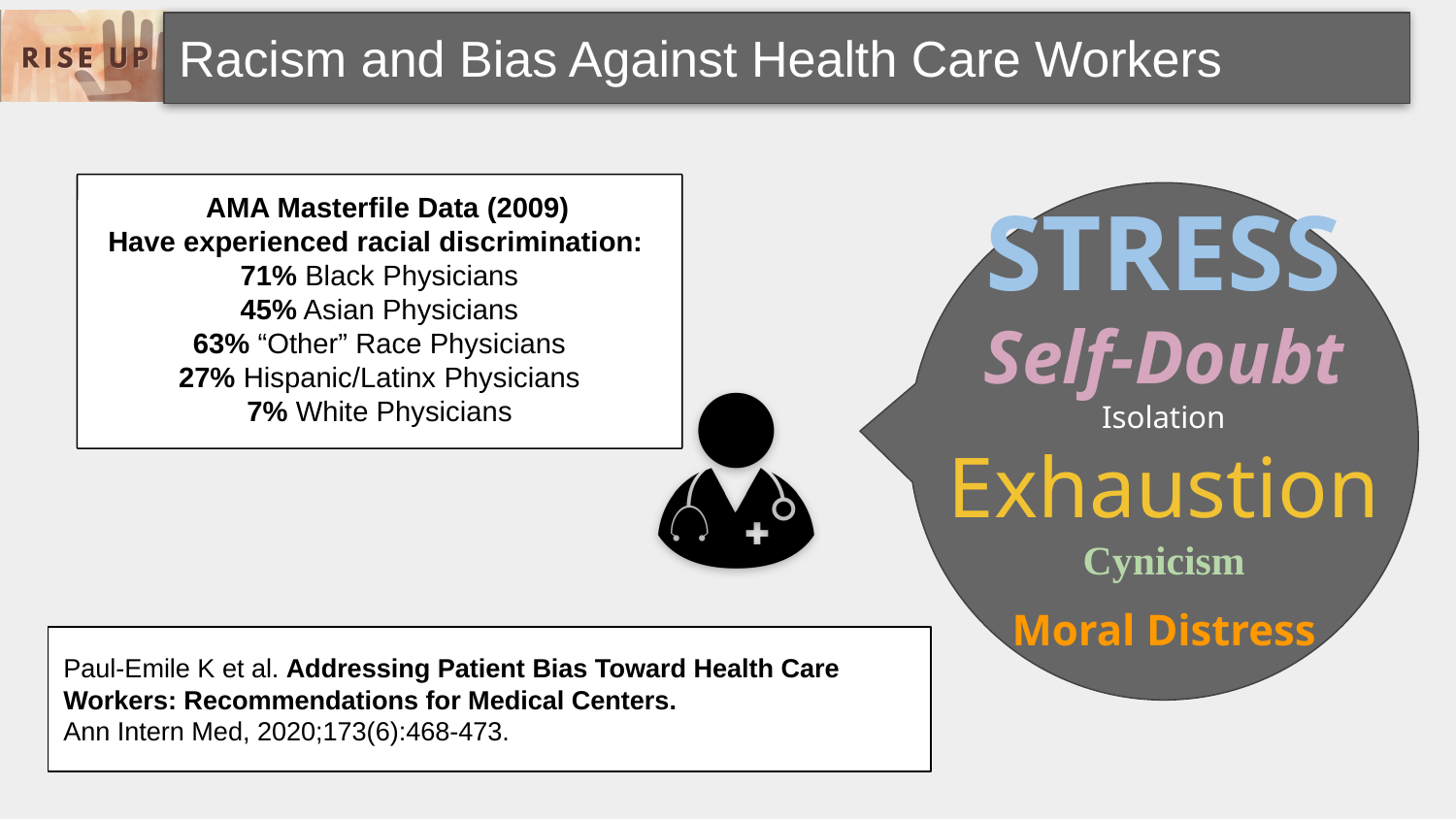

# Racism and Bias Against Health Care Workers
 AMA Masterfile Data (2009)
Have experienced racial discrimination:
71% Black Physicians
45% Asian Physicians
63% “Other” Race Physicians
27% Hispanic/Latinx Physicians
7% White Physicians
STRESS
Self-Doubt
Isolation
Exhaustion
Cynicism
Moral Distress
Paul-Emile K et al. Addressing Patient Bias Toward Health Care Workers: Recommendations for Medical Centers.
Ann Intern Med, 2020;173(6):468-473.

## Slide 11
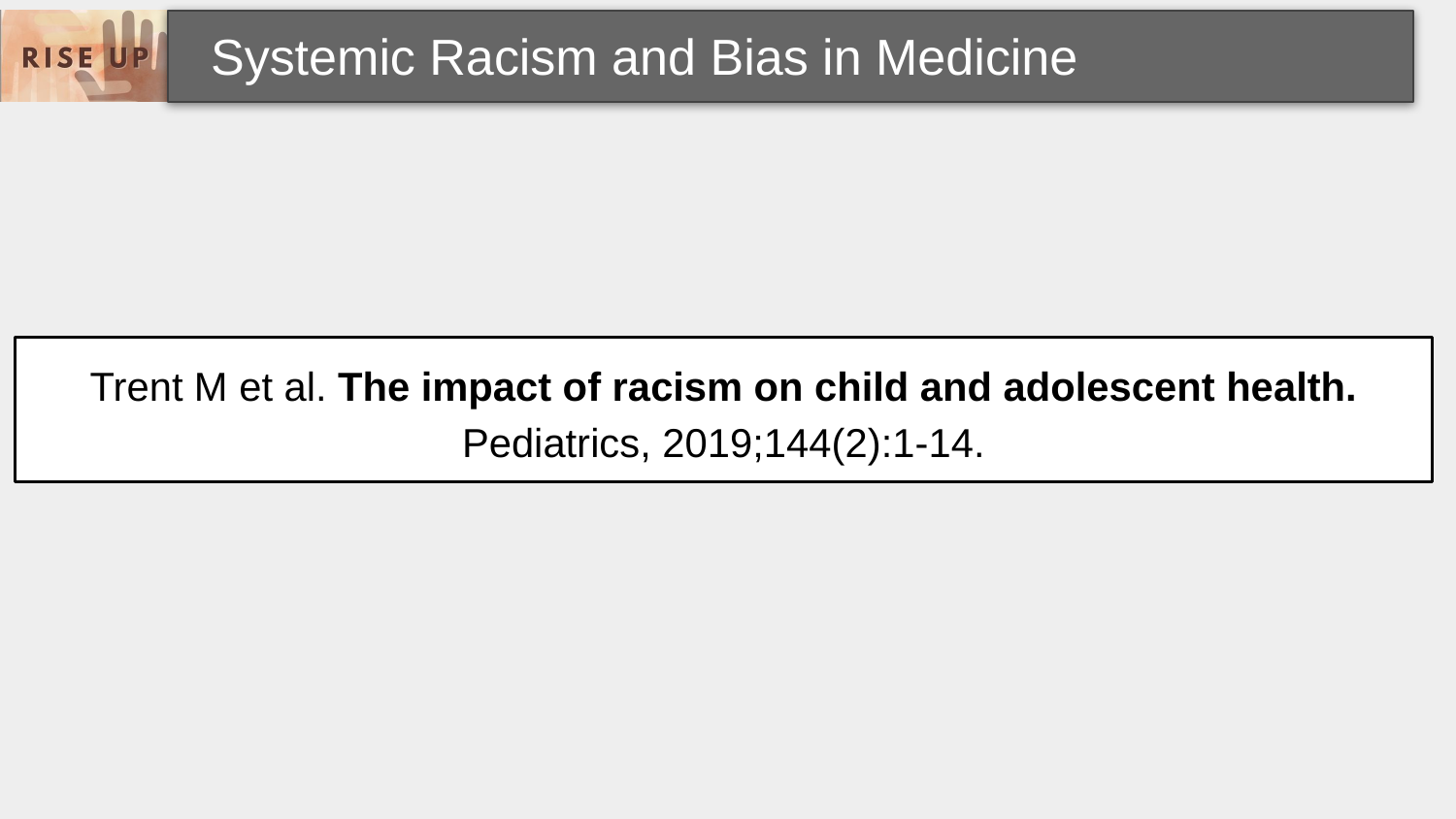

# Systemic Racism and Bias in Medicine
Trent M et al. The impact of racism on child and adolescent health. Pediatrics, 2019;144(2):1-14.

## Slide 12
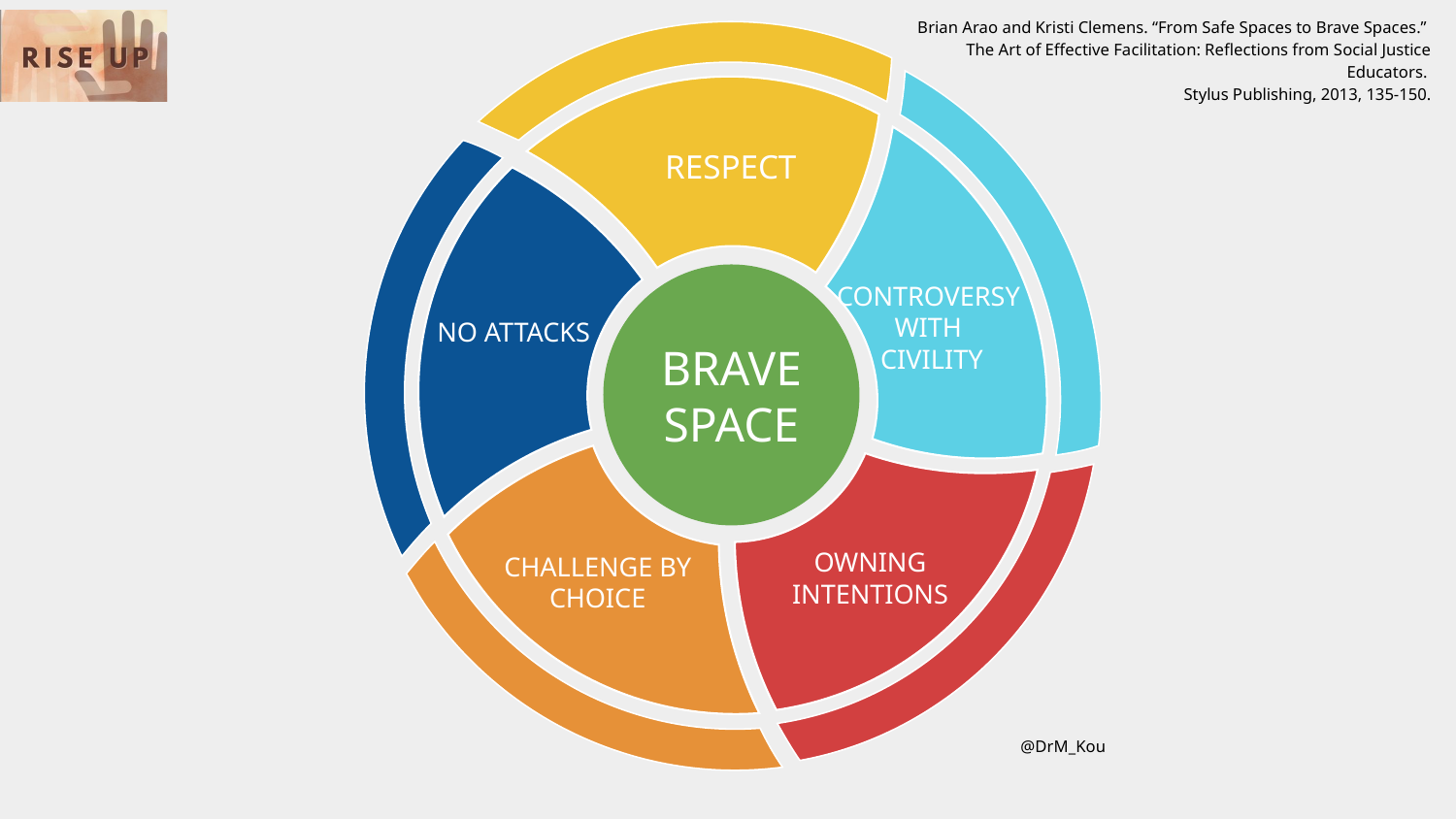

RESPECT
CONTROVERSY
WITH
CIVILITY
NO ATTACKS
BRAVE SPACE
OWNING INTENTIONS
CHALLENGE BY CHOICE
Brian Arao and Kristi Clemens. “From Safe Spaces to Brave Spaces.”
The Art of Effective Facilitation: Reflections from Social Justice Educators.
Stylus Publishing, 2013, 135-150.
@DrM_Kou

## Slide 13
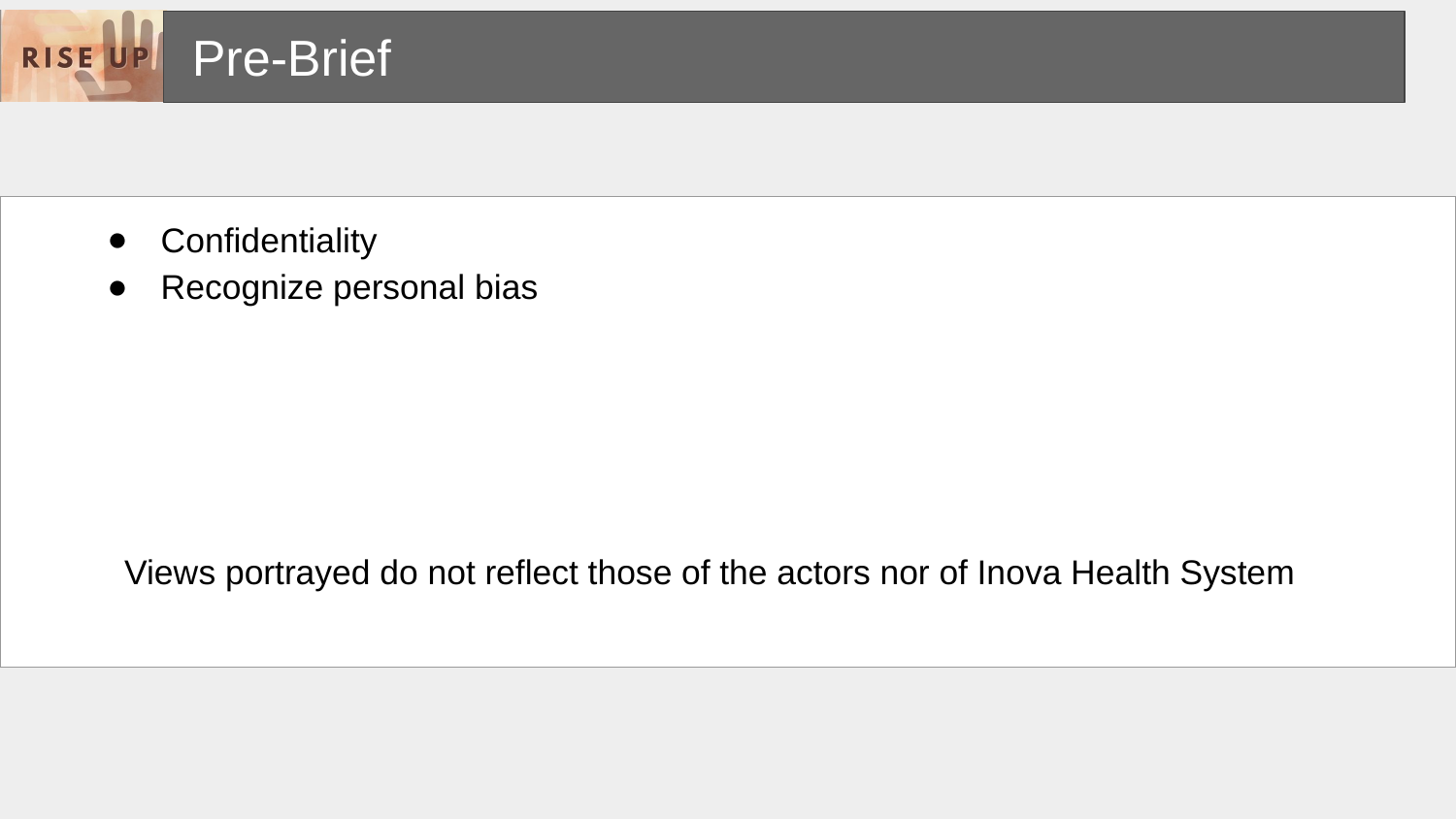

# Pre-Brief
Confidentiality
Recognize personal bias
Views portrayed do not reflect those of the actors nor of Inova Health System

## Slide 14
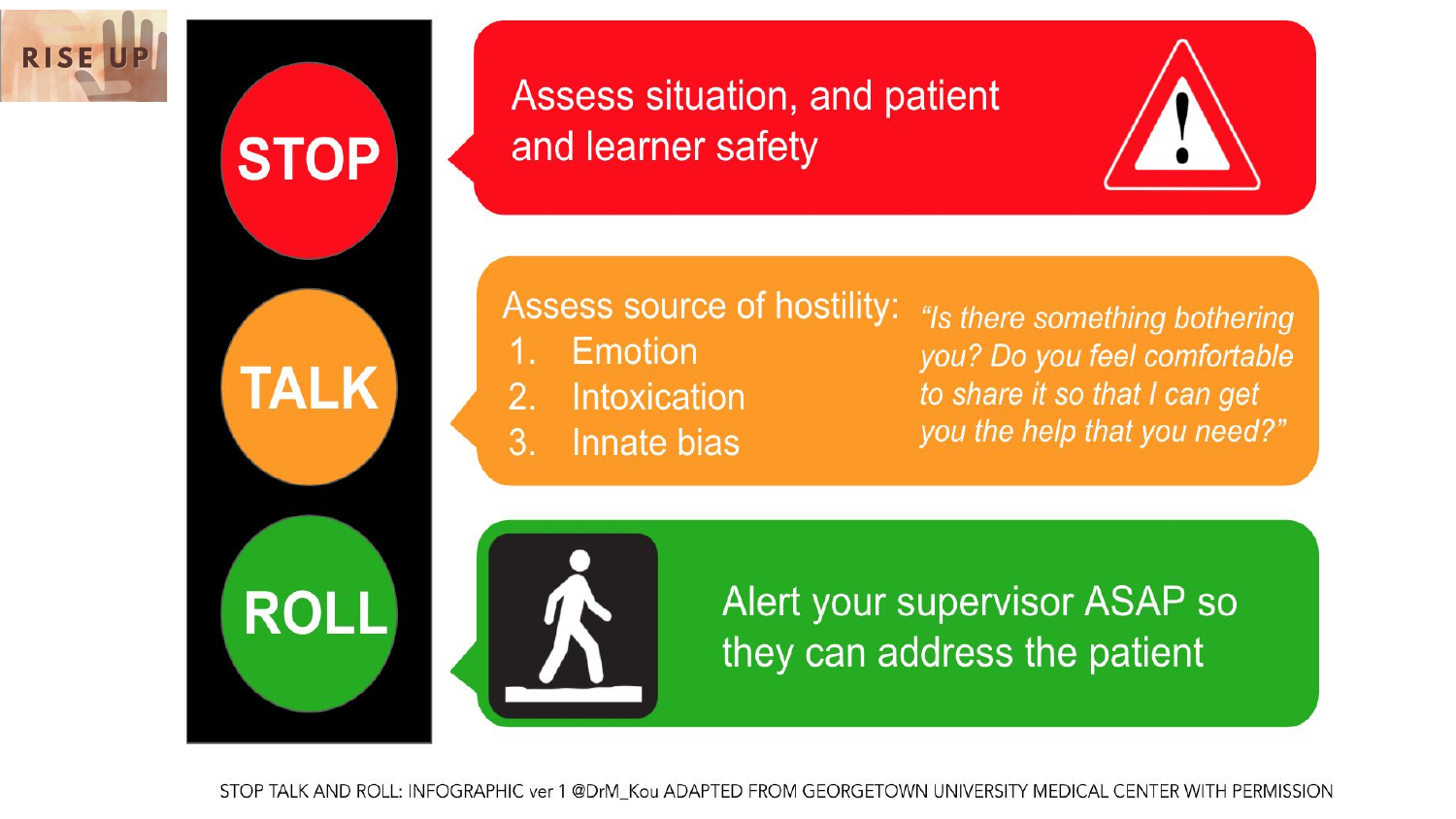

## Slide 15
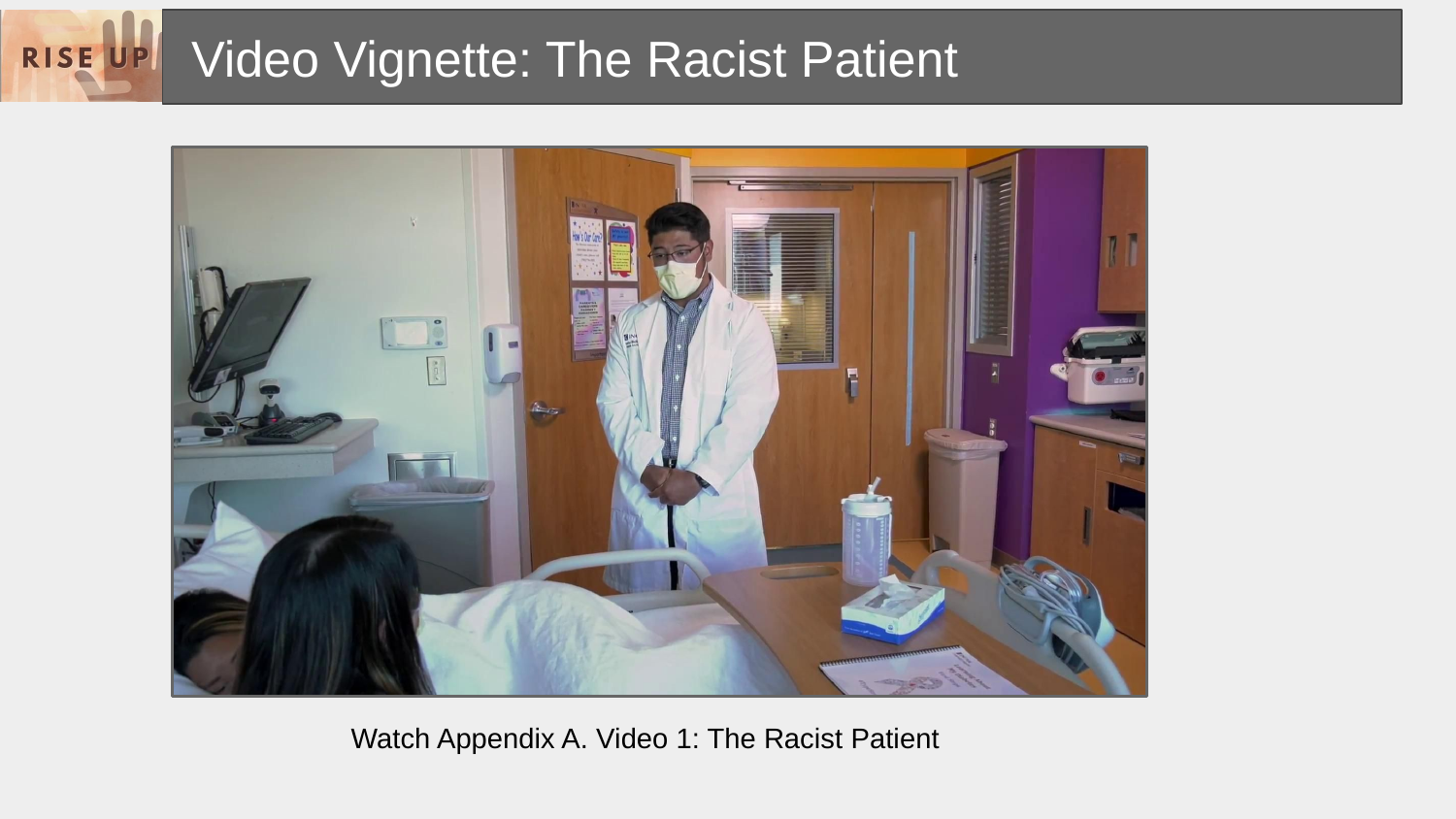

# Video Vignette: The Racist Patient
Watch Appendix A. Video 1: The Racist Patient

## Slide 16
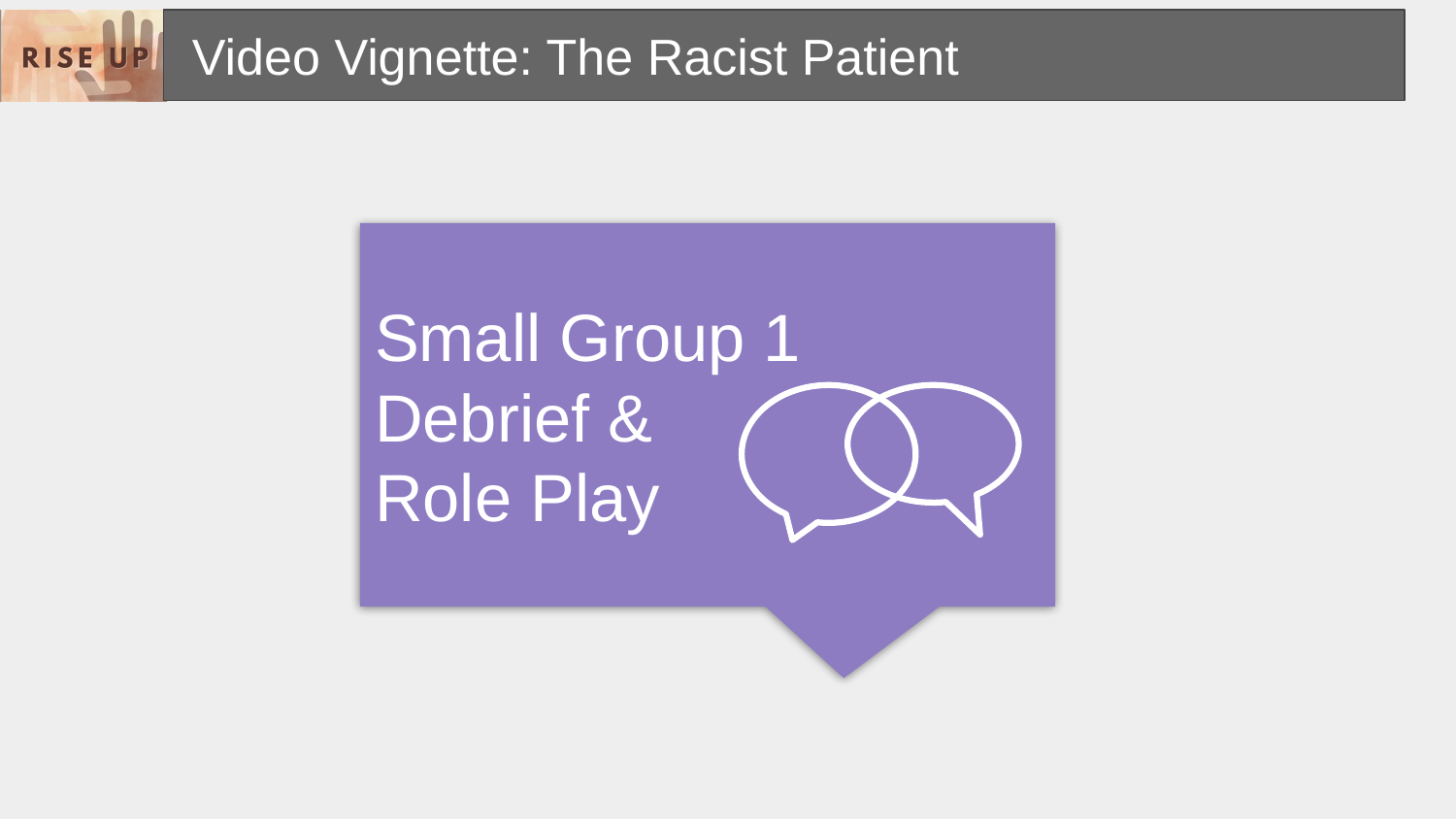

# Video Vignette: The Racist Patient
Small Group 1
Debrief &
Role Play

## Slide 17
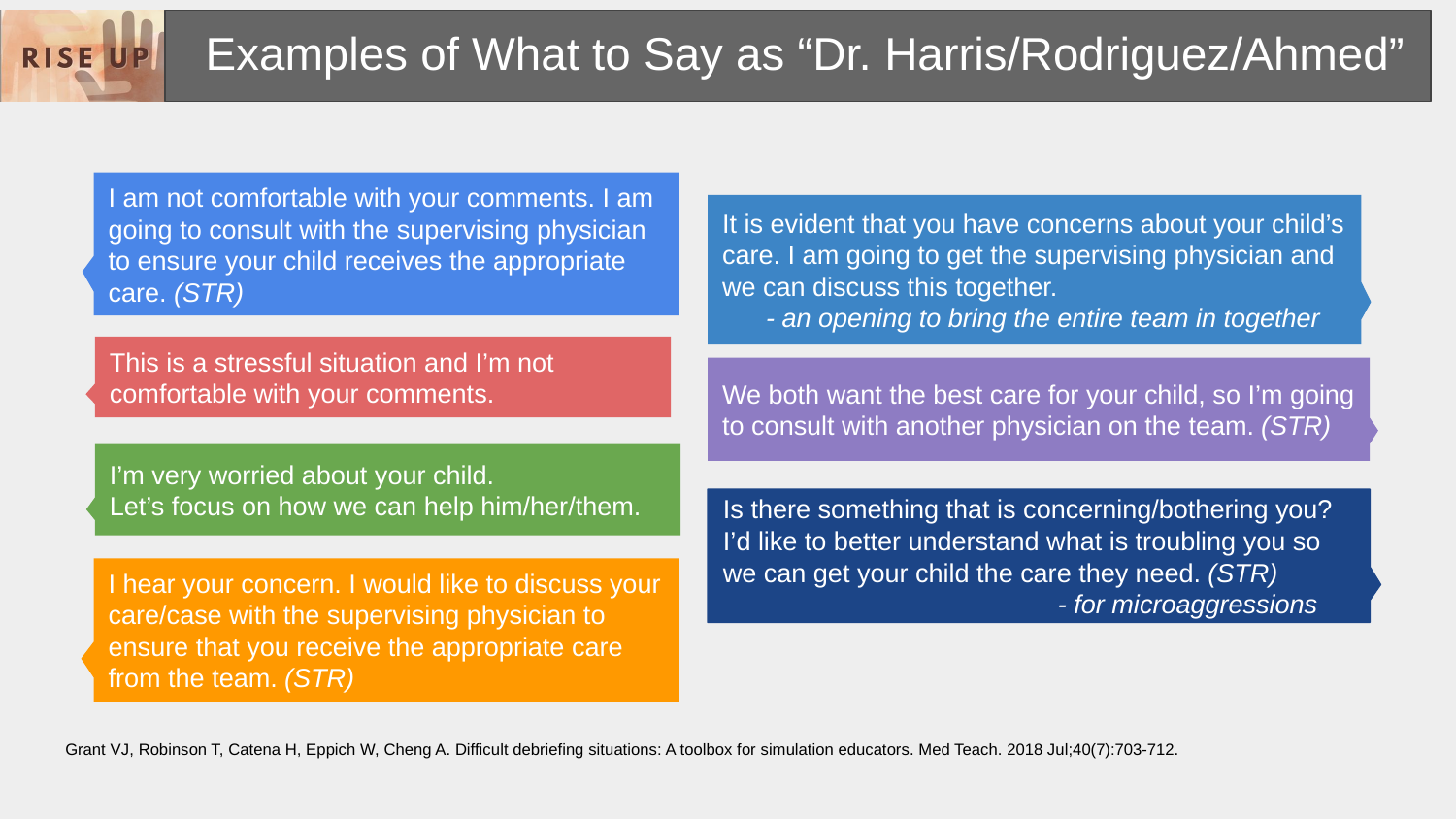

# Examples of What to Say as “Dr. Harris/Rodriguez/Ahmed”
I am not comfortable with your comments. I am going to consult with the supervising physician to ensure your child receives the appropriate care. (STR)
It is evident that you have concerns about your child’s care. I am going to get the supervising physician and we can discuss this together.
 - an opening to bring the entire team in together
This is a stressful situation and I’m not comfortable with your comments.
We both want the best care for your child, so I’m going to consult with another physician on the team. (STR)
I’m very worried about your child.
Let’s focus on how we can help him/her/them.
Is there something that is concerning/bothering you? I’d like to better understand what is troubling you so we can get your child the care they need. (STR)
 - for microaggressions
I hear your concern. I would like to discuss your care/case with the supervising physician to ensure that you receive the appropriate care from the team. (STR)
Grant VJ, Robinson T, Catena H, Eppich W, Cheng A. Difficult debriefing situations: A toolbox for simulation educators. Med Teach. 2018 Jul;40(7):703-712.

## Slide 18
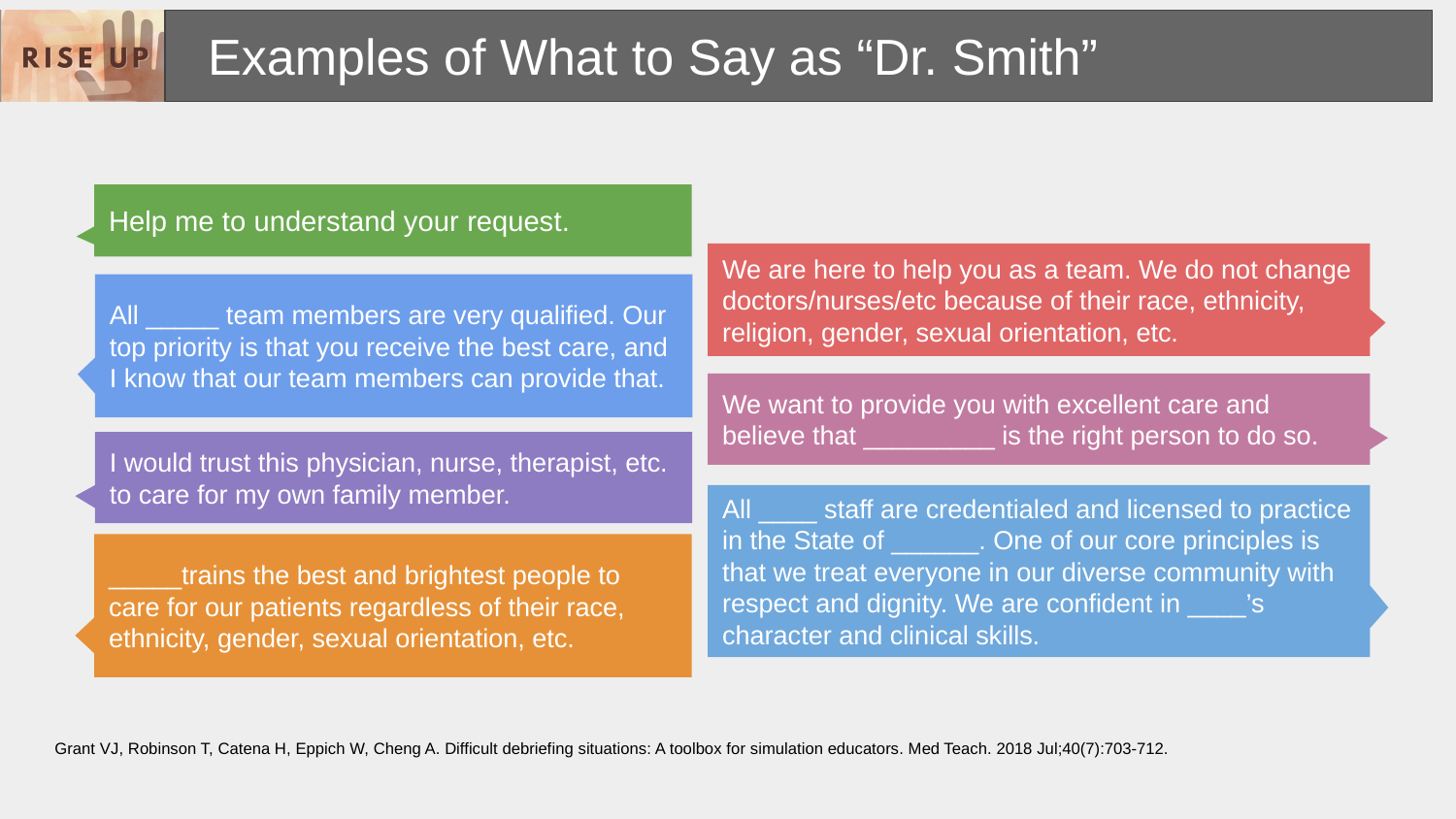

# Examples of What to Say as “Dr. Smith”
Help me to understand your request.
We are here to help you as a team. We do not change doctors/nurses/etc because of their race, ethnicity, religion, gender, sexual orientation, etc.
All _____ team members are very qualified. Our top priority is that you receive the best care, and I know that our team members can provide that.
We want to provide you with excellent care and believe that _________ is the right person to do so.
I would trust this physician, nurse, therapist, etc. to care for my own family member.
All ____ staff are credentialed and licensed to practice in the State of ______. One of our core principles is that we treat everyone in our diverse community with respect and dignity. We are confident in ____’s character and clinical skills.
_____trains the best and brightest people to care for our patients regardless of their race, ethnicity, gender, sexual orientation, etc.
Grant VJ, Robinson T, Catena H, Eppich W, Cheng A. Difficult debriefing situations: A toolbox for simulation educators. Med Teach. 2018 Jul;40(7):703-712.

## Slide 19
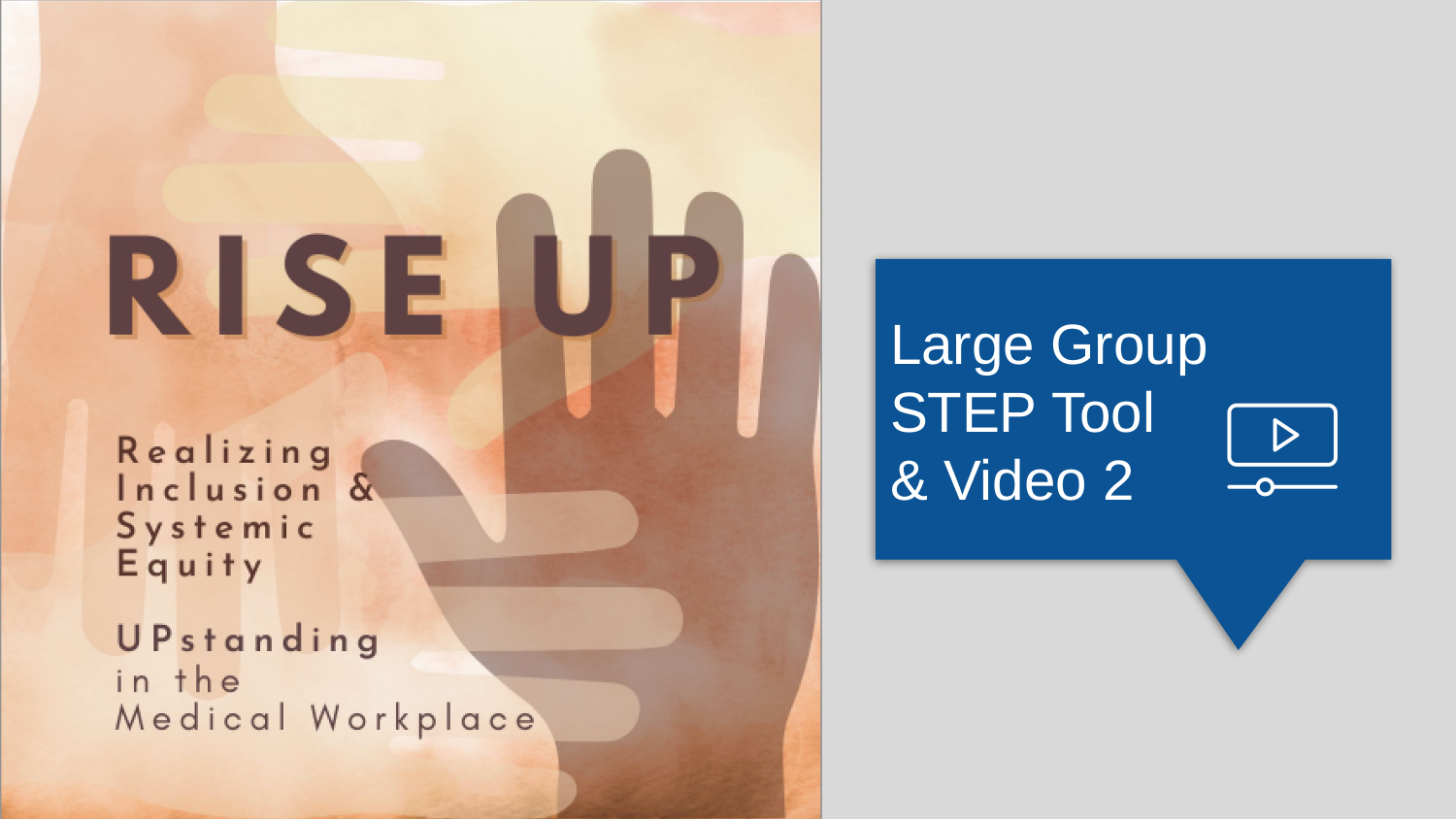

Large Group
STEP Tool
& Video 2

## Slide 20
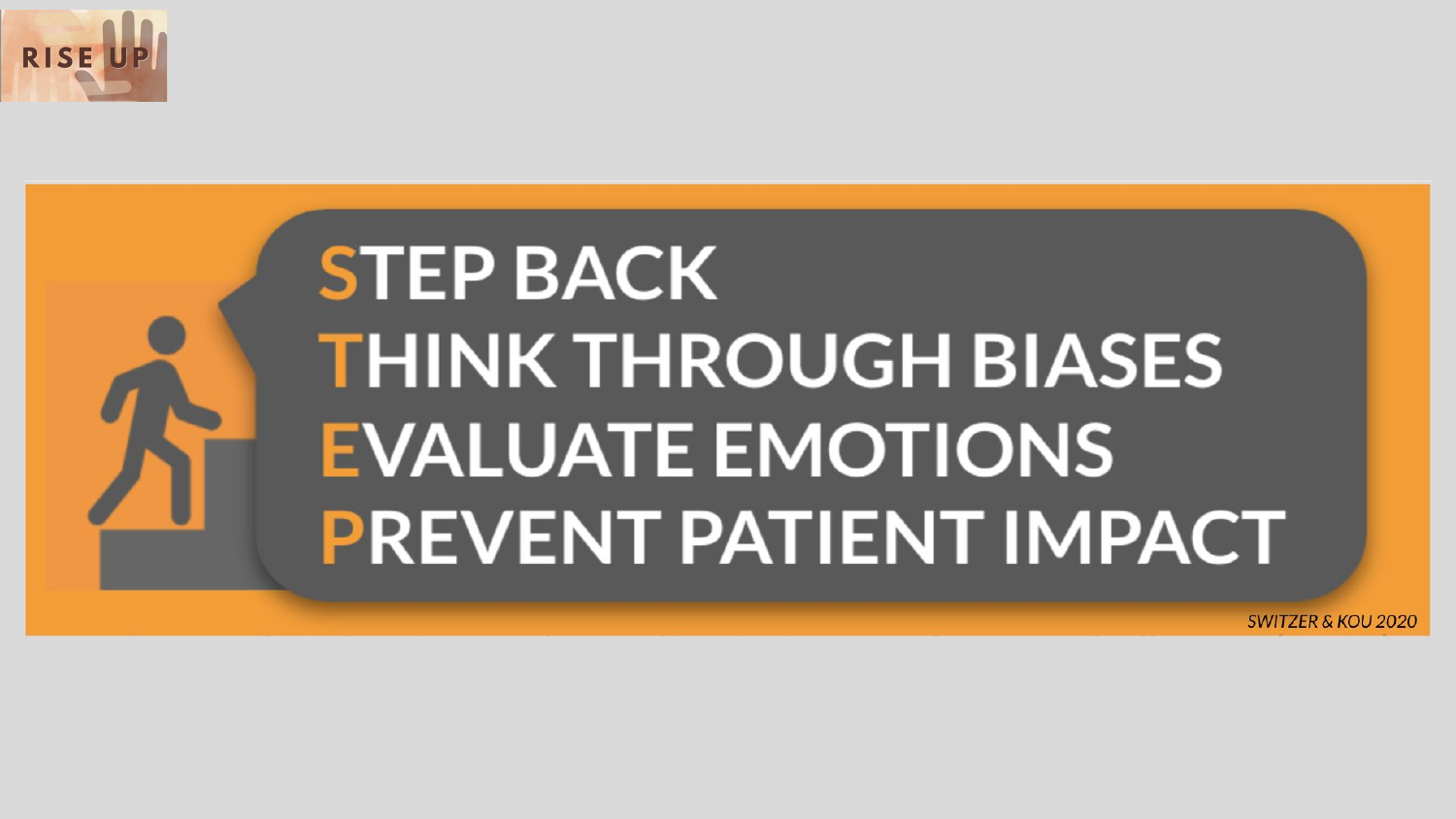

## Slide 21
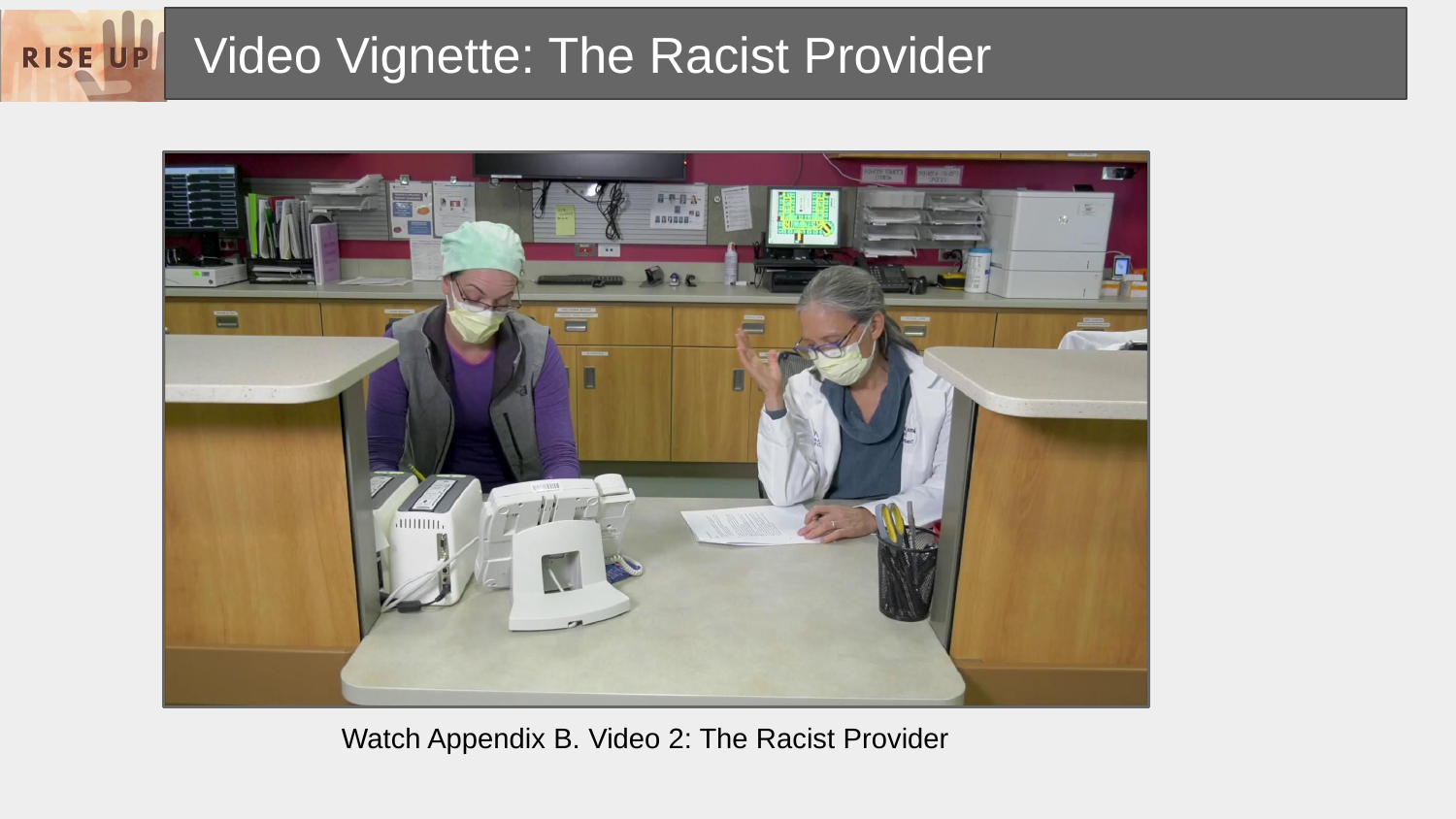

# Video Vignette: The Racist Provider
Watch Appendix B. Video 2: The Racist Provider

## Slide 22
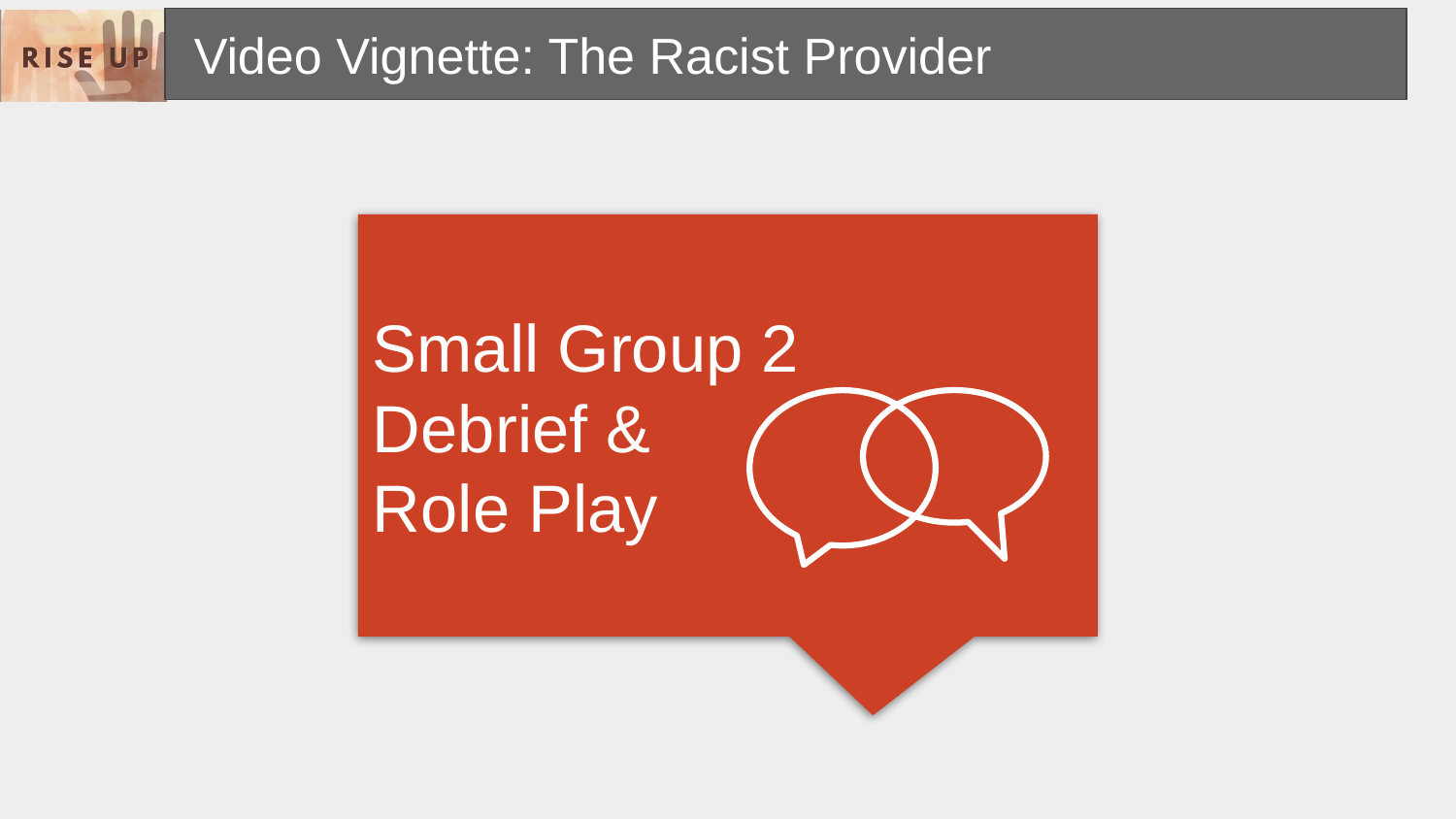

# Video Vignette: The Racist Provider
Small Group 2
Debrief &
Role Play

## Slide 23
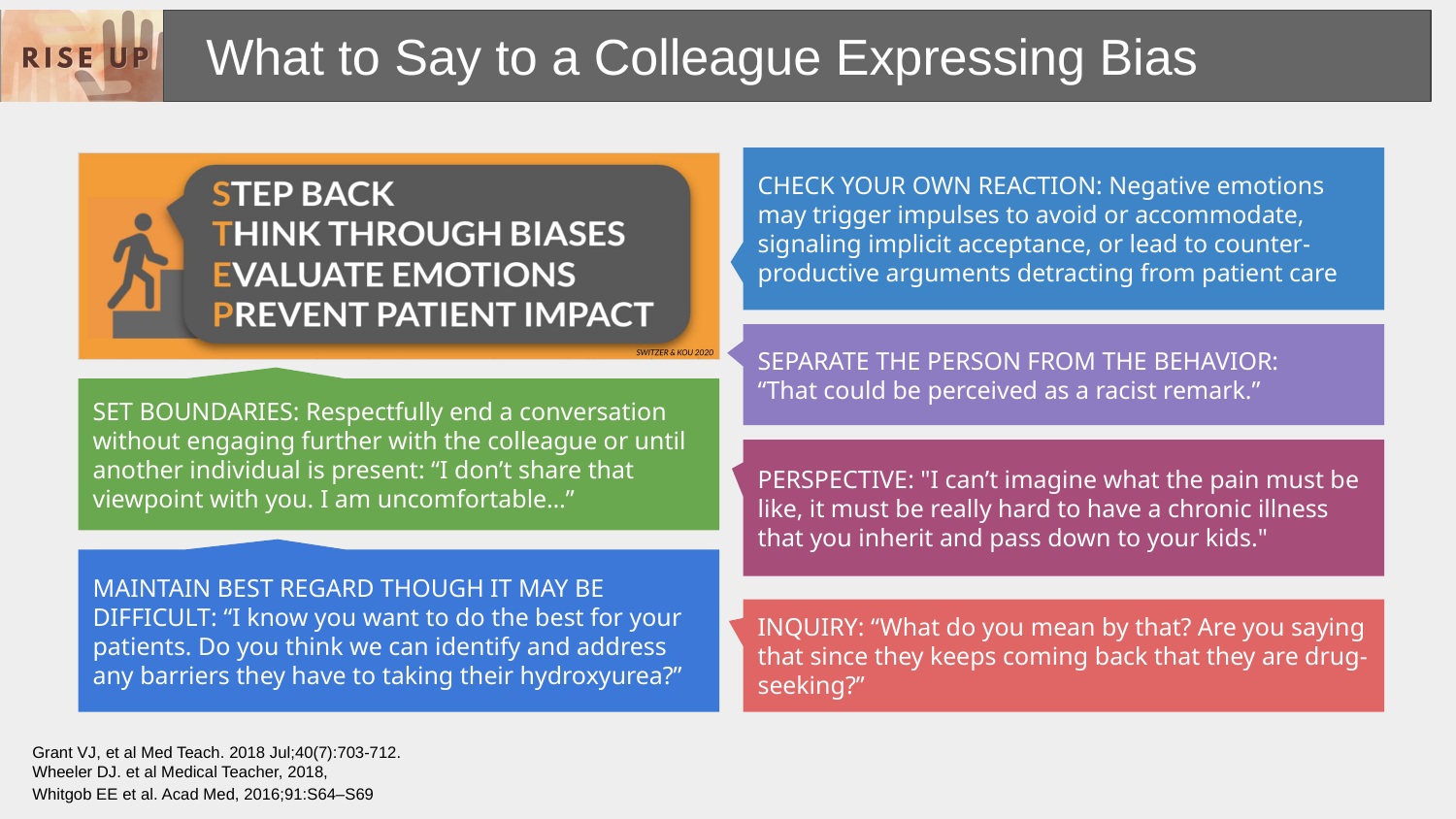

# What to Say to a Colleague Expressing Bias
CHECK YOUR OWN REACTION: Negative emotions may trigger impulses to avoid or accommodate, signaling implicit acceptance, or lead to counter-productive arguments detracting from patient care
SEPARATE THE PERSON FROM THE BEHAVIOR:
“That could be perceived as a racist remark.”
SET BOUNDARIES: Respectfully end a conversation without engaging further with the colleague or until another individual is present: “I don’t share that viewpoint with you. I am uncomfortable…”
PERSPECTIVE: "I can’t imagine what the pain must be like, it must be really hard to have a chronic illness that you inherit and pass down to your kids."
MAINTAIN BEST REGARD THOUGH IT MAY BE DIFFICULT: “I know you want to do the best for your patients. Do you think we can identify and address any barriers they have to taking their hydroxyurea?”
INQUIRY: “What do you mean by that? Are you saying that since they keeps coming back that they are drug-seeking?”
Grant VJ, et al Med Teach. 2018 Jul;40(7):703-712.
Wheeler DJ. et al Medical Teacher, 2018,
Whitgob EE et al. Acad Med, 2016;91:S64–S69

## Slide 24
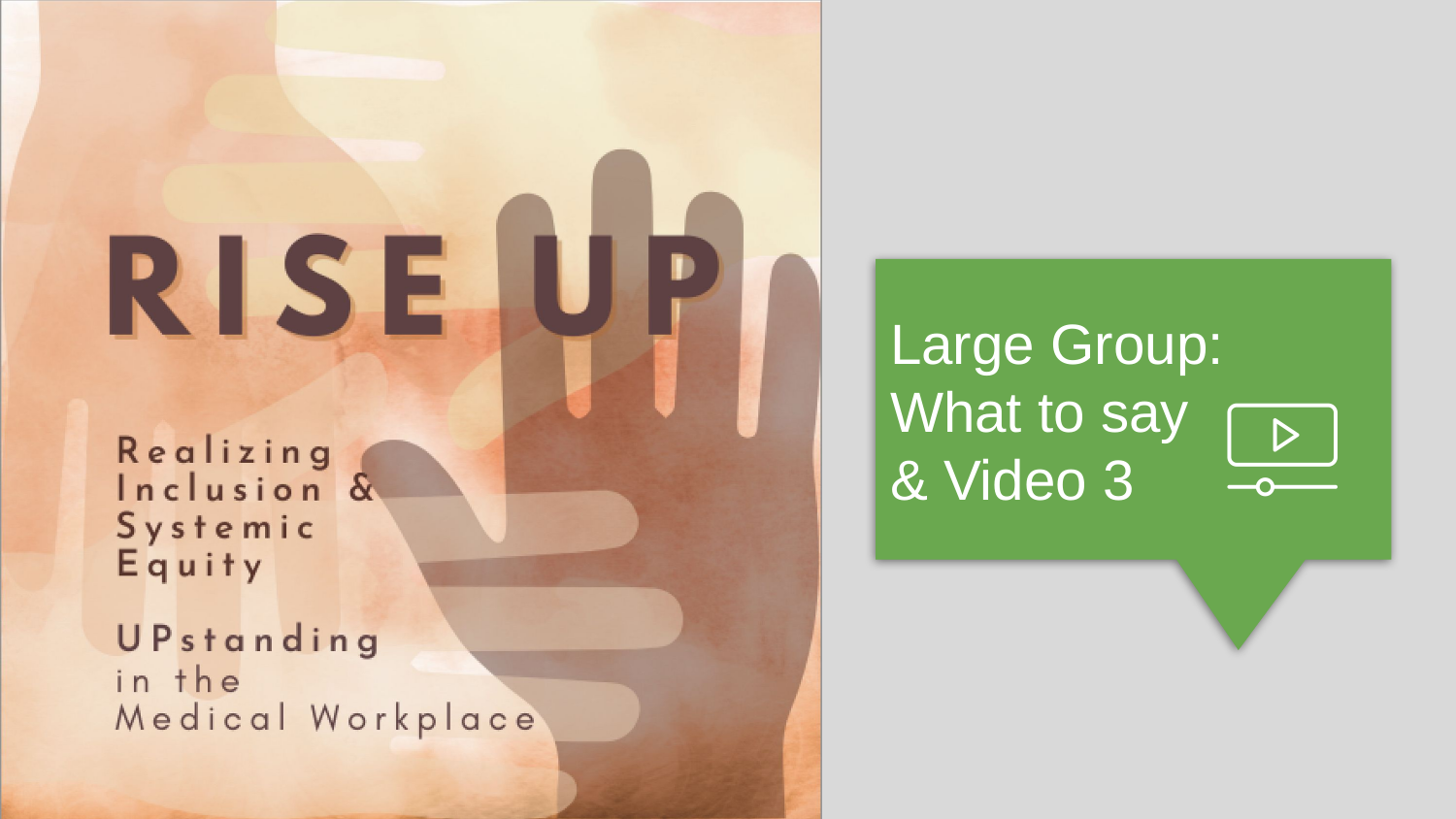

Large Group:
What to say
& Video 3

## Slide 25
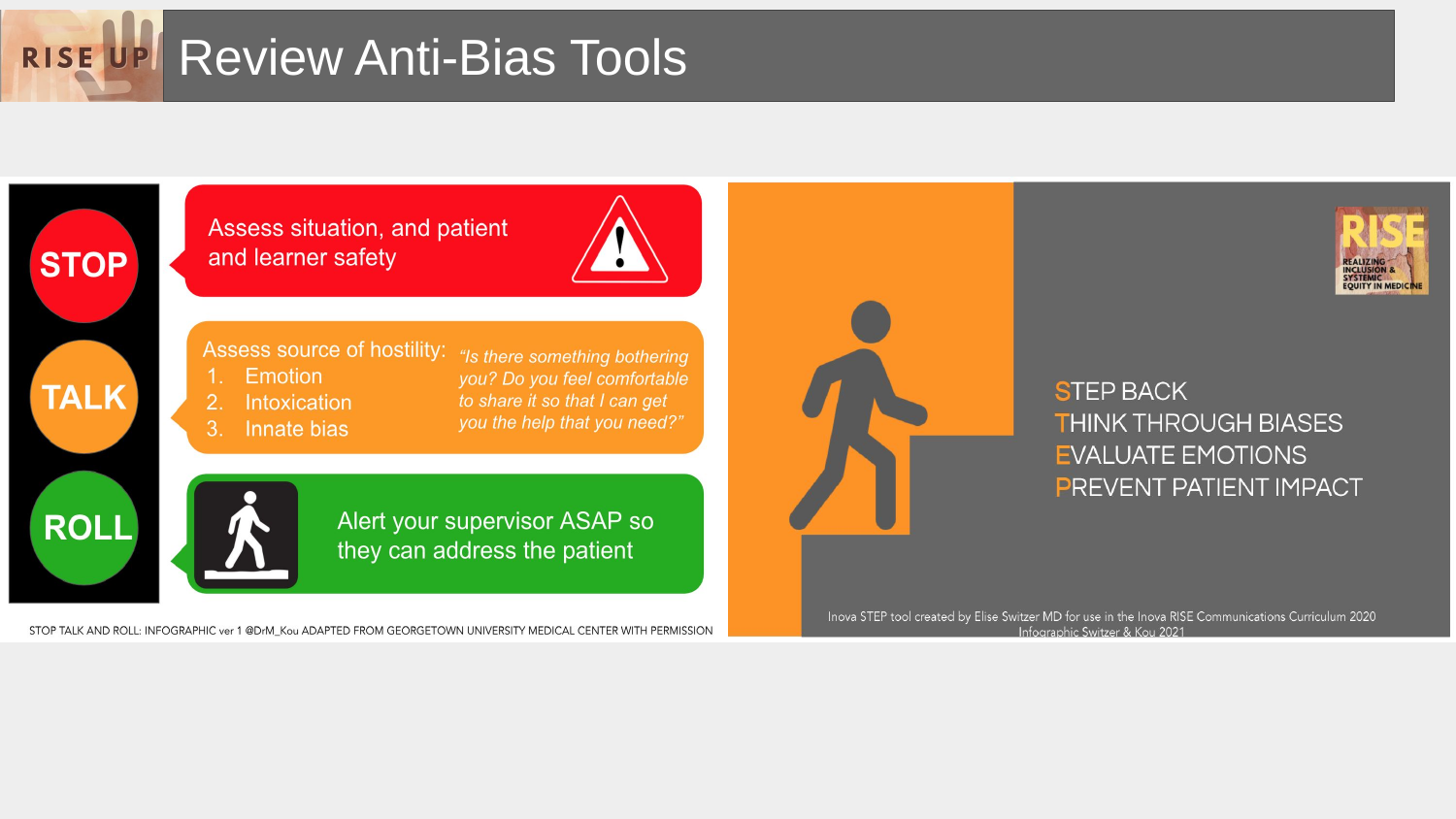

# Review Anti-Bias Tools

## Slide 26
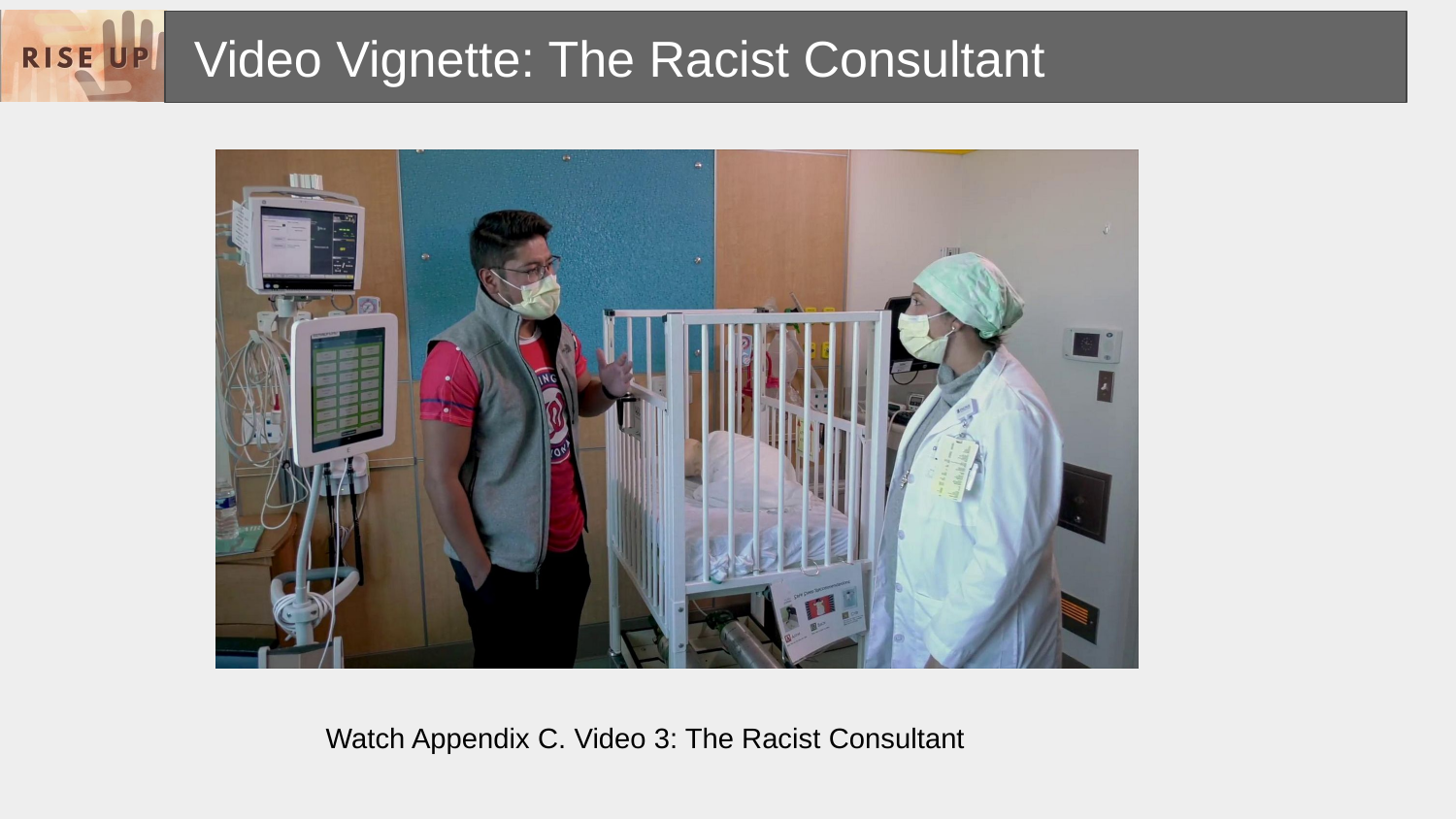

# Video Vignette: The Racist Consultant
Watch Appendix C. Video 3: The Racist Consultant

## Slide 27
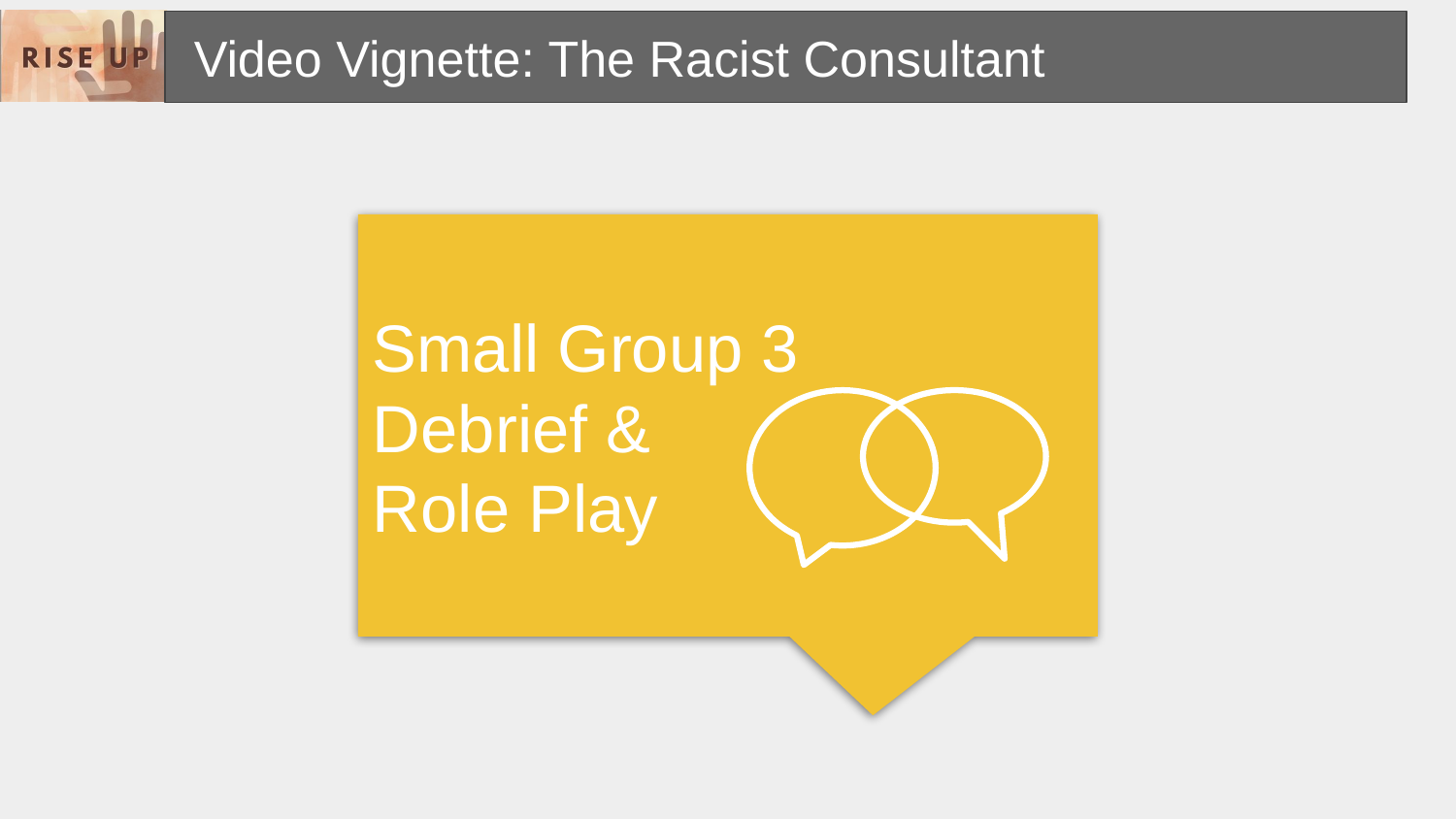

# Video Vignette: The Racist Consultant
Small Group 3
Debrief &
Role Play

## Slide 28
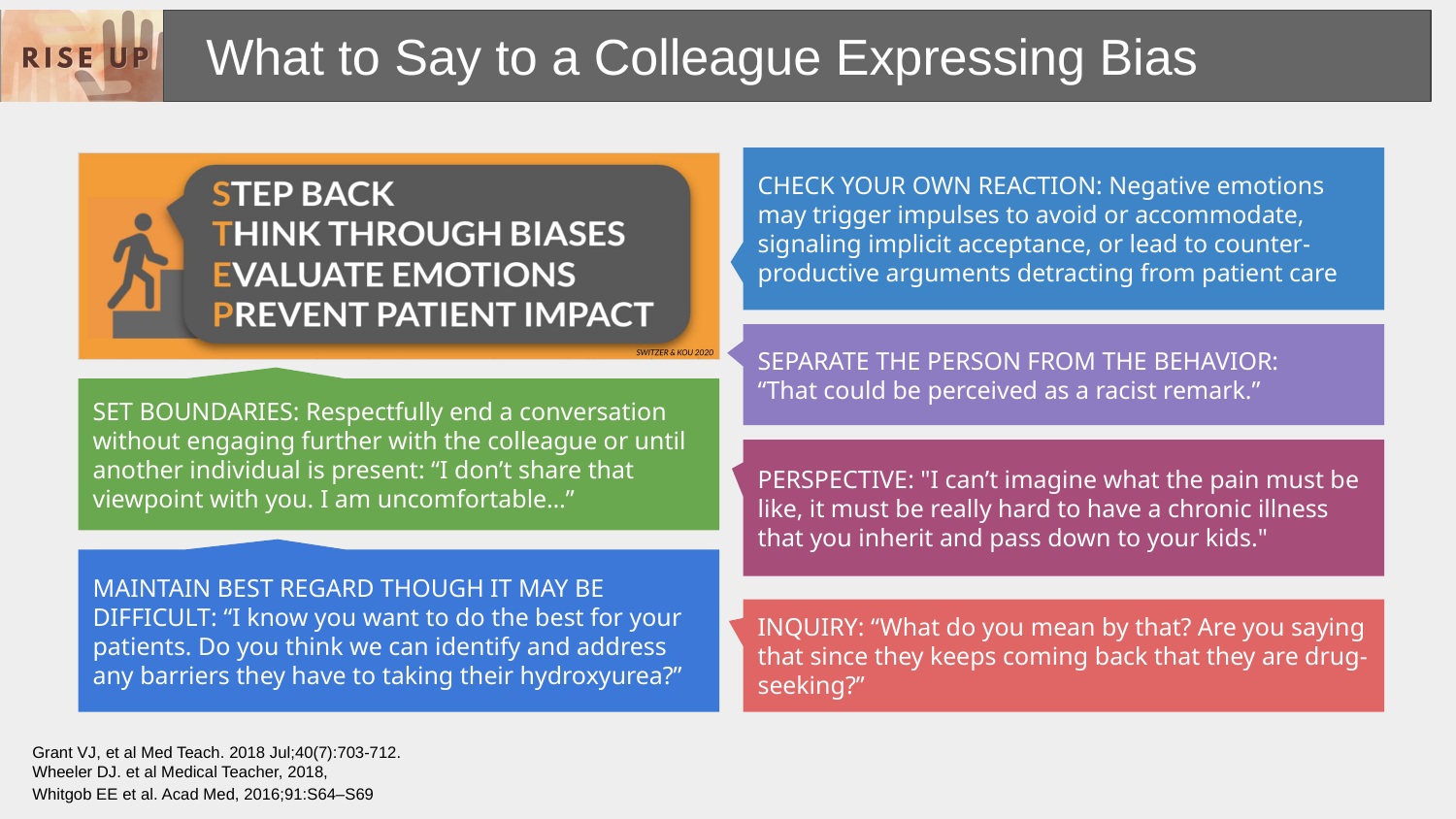

# What to Say to a Colleague Expressing Bias
CHECK YOUR OWN REACTION: Negative emotions may trigger impulses to avoid or accommodate, signaling implicit acceptance, or lead to counter-productive arguments detracting from patient care
SEPARATE THE PERSON FROM THE BEHAVIOR:
“That could be perceived as a racist remark.”
SET BOUNDARIES: Respectfully end a conversation without engaging further with the colleague or until another individual is present: “I don’t share that viewpoint with you. I am uncomfortable…”
PERSPECTIVE: "I can’t imagine what the pain must be like, it must be really hard to have a chronic illness that you inherit and pass down to your kids."
MAINTAIN BEST REGARD THOUGH IT MAY BE DIFFICULT: “I know you want to do the best for your patients. Do you think we can identify and address any barriers they have to taking their hydroxyurea?”
INQUIRY: “What do you mean by that? Are you saying that since they keeps coming back that they are drug-seeking?”
Grant VJ, et al Med Teach. 2018 Jul;40(7):703-712.
Wheeler DJ. et al Medical Teacher, 2018,
Whitgob EE et al. Acad Med, 2016;91:S64–S69

## Slide 29
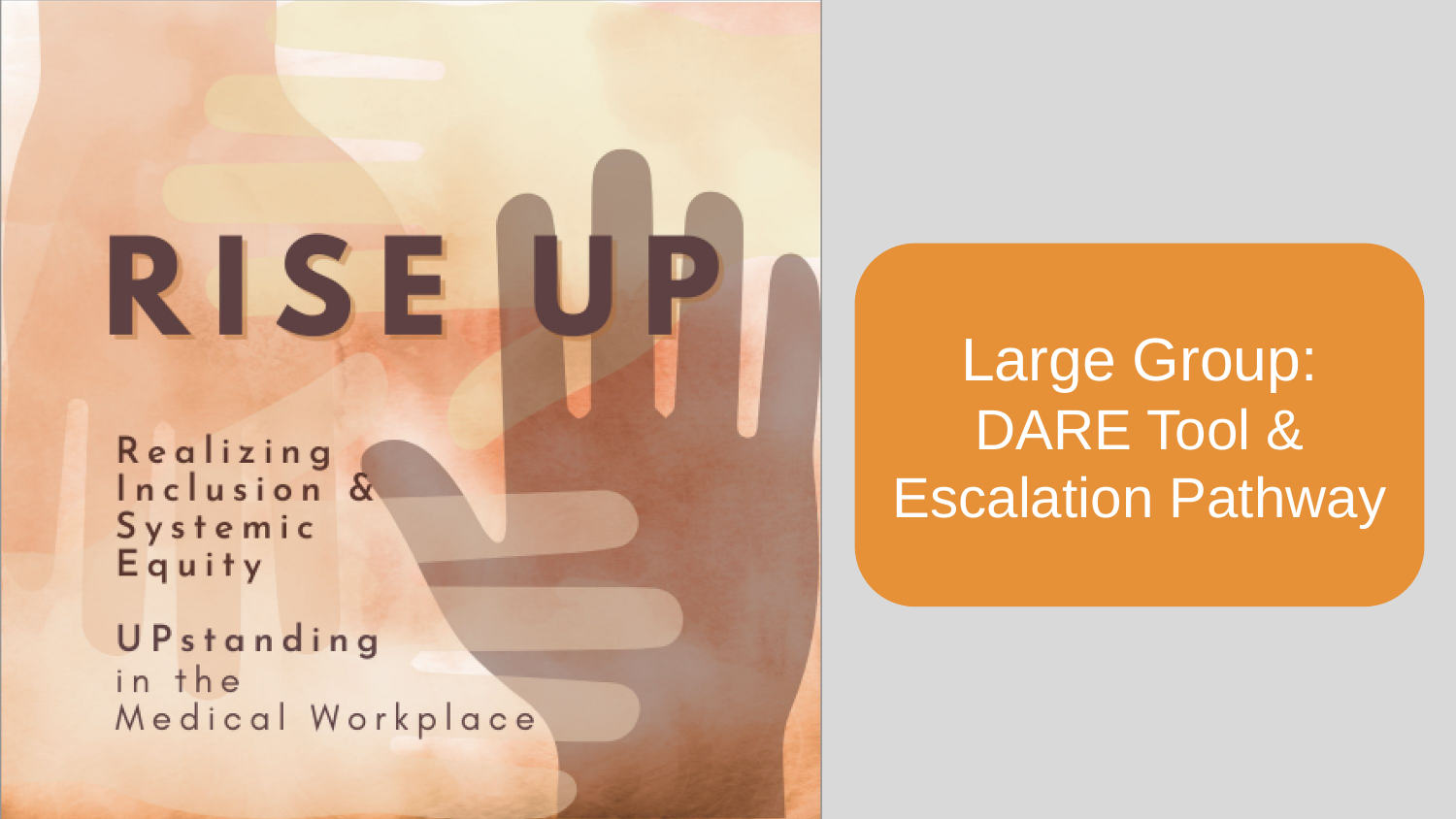

Large Group: DARE Tool & Escalation Pathway

## Slide 30
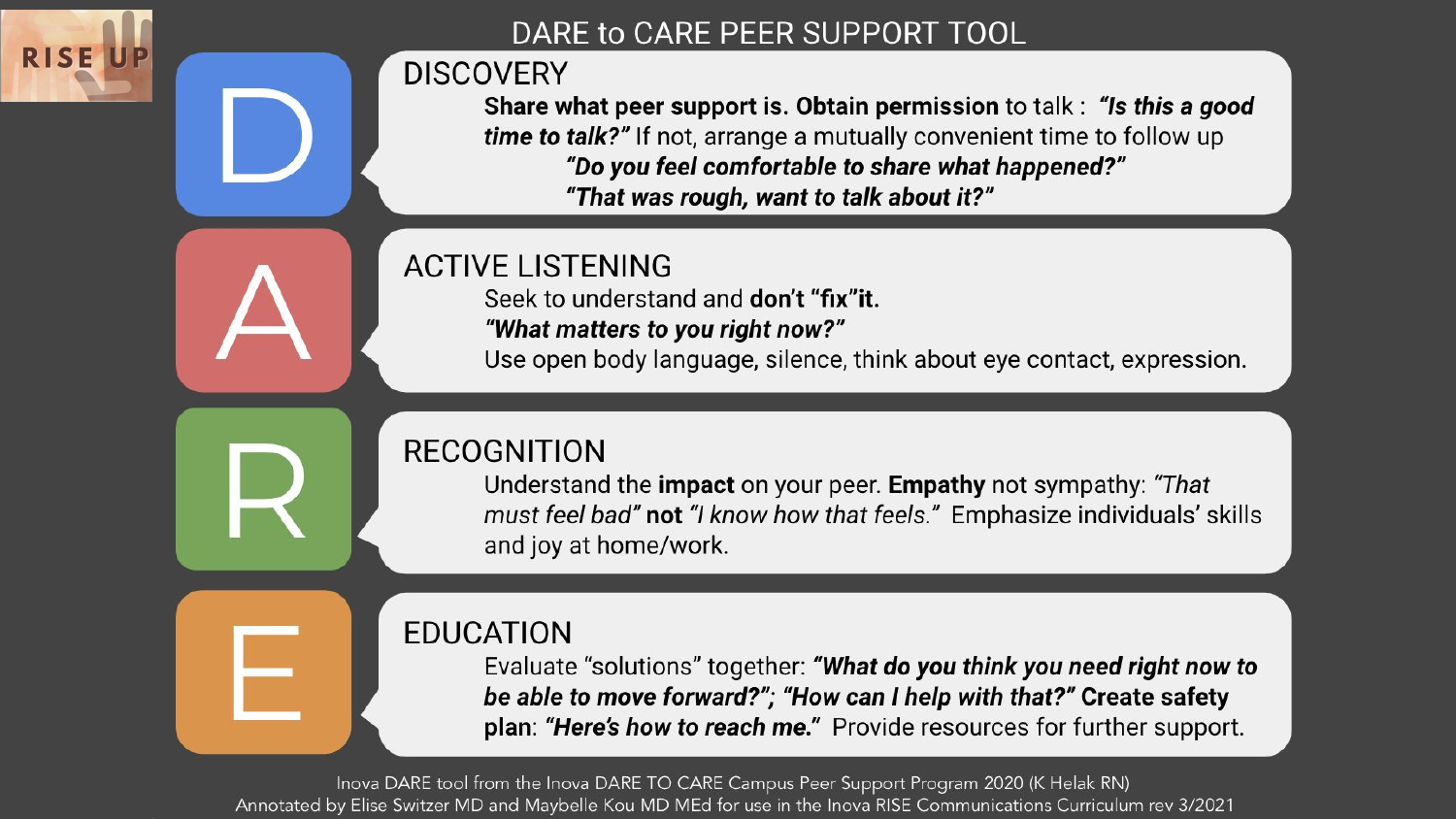

## Slide 31
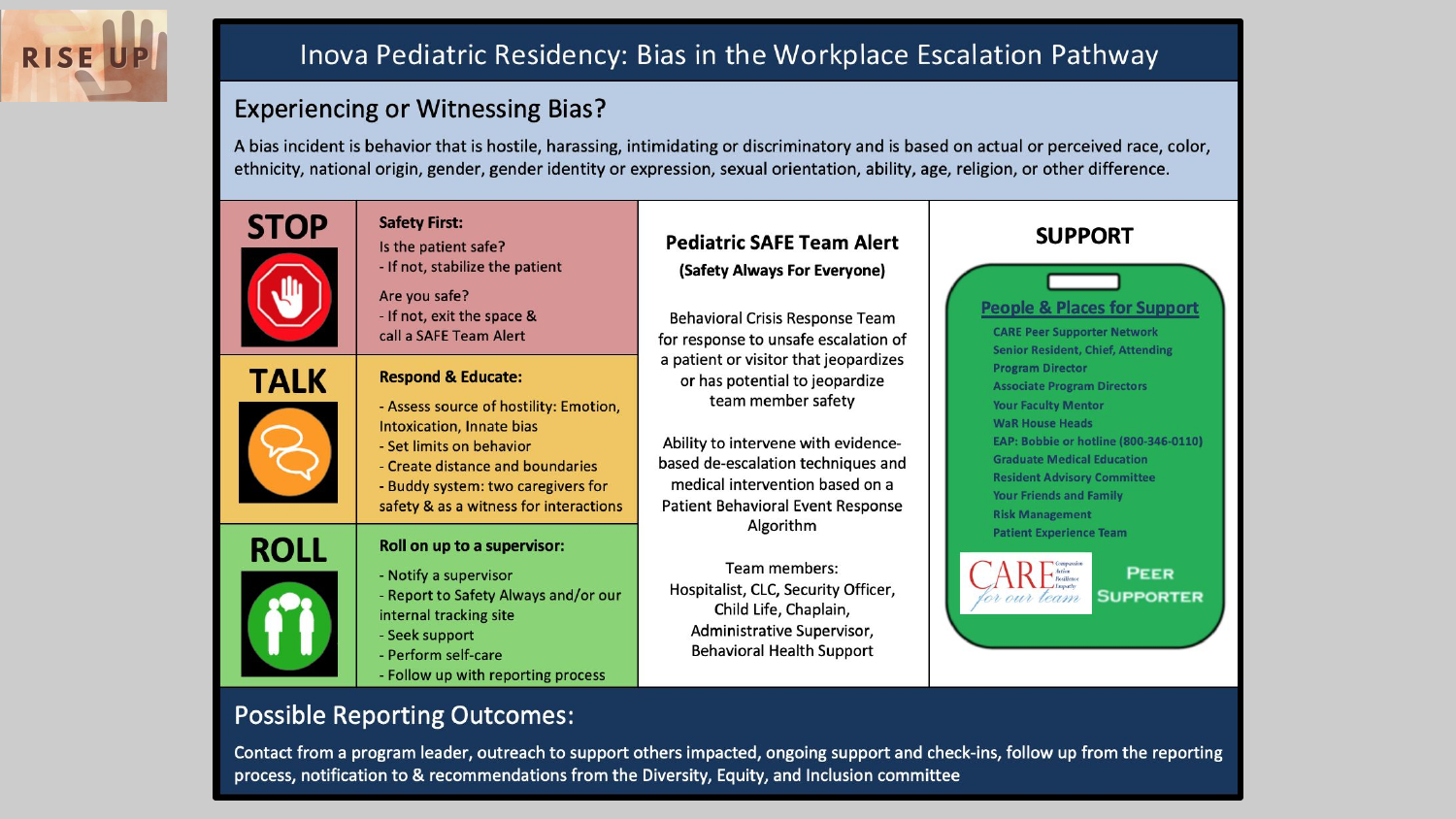

## Slide 32
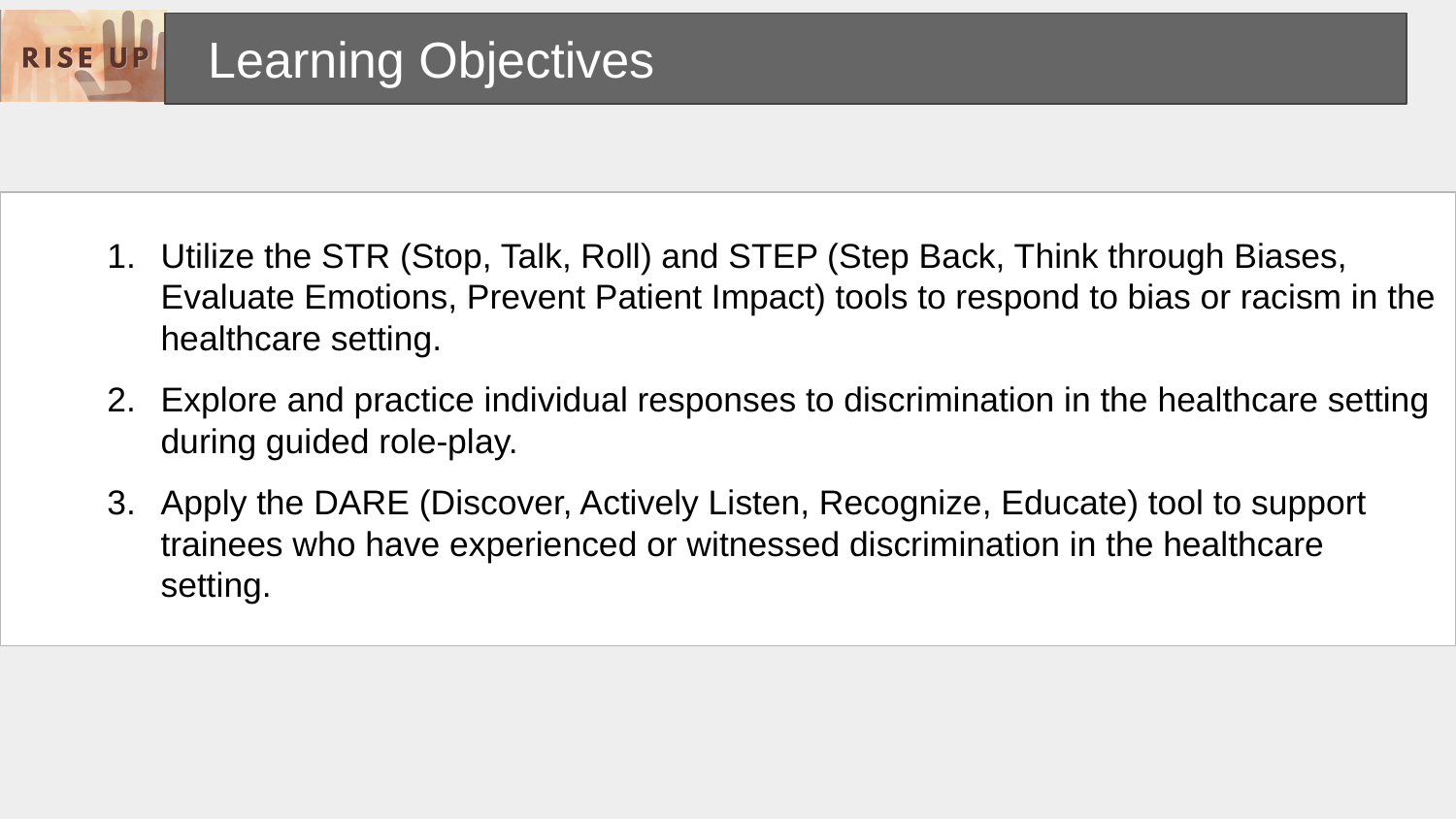

# Learning Objectives
Utilize the STR (Stop, Talk, Roll) and STEP (Step Back, Think through Biases, Evaluate Emotions, Prevent Patient Impact) tools to respond to bias or racism in the healthcare setting.
Explore and practice individual responses to discrimination in the healthcare setting during guided role-play.
Apply the DARE (Discover, Actively Listen, Recognize, Educate) tool to support trainees who have experienced or witnessed discrimination in the healthcare setting.

## Slide 33
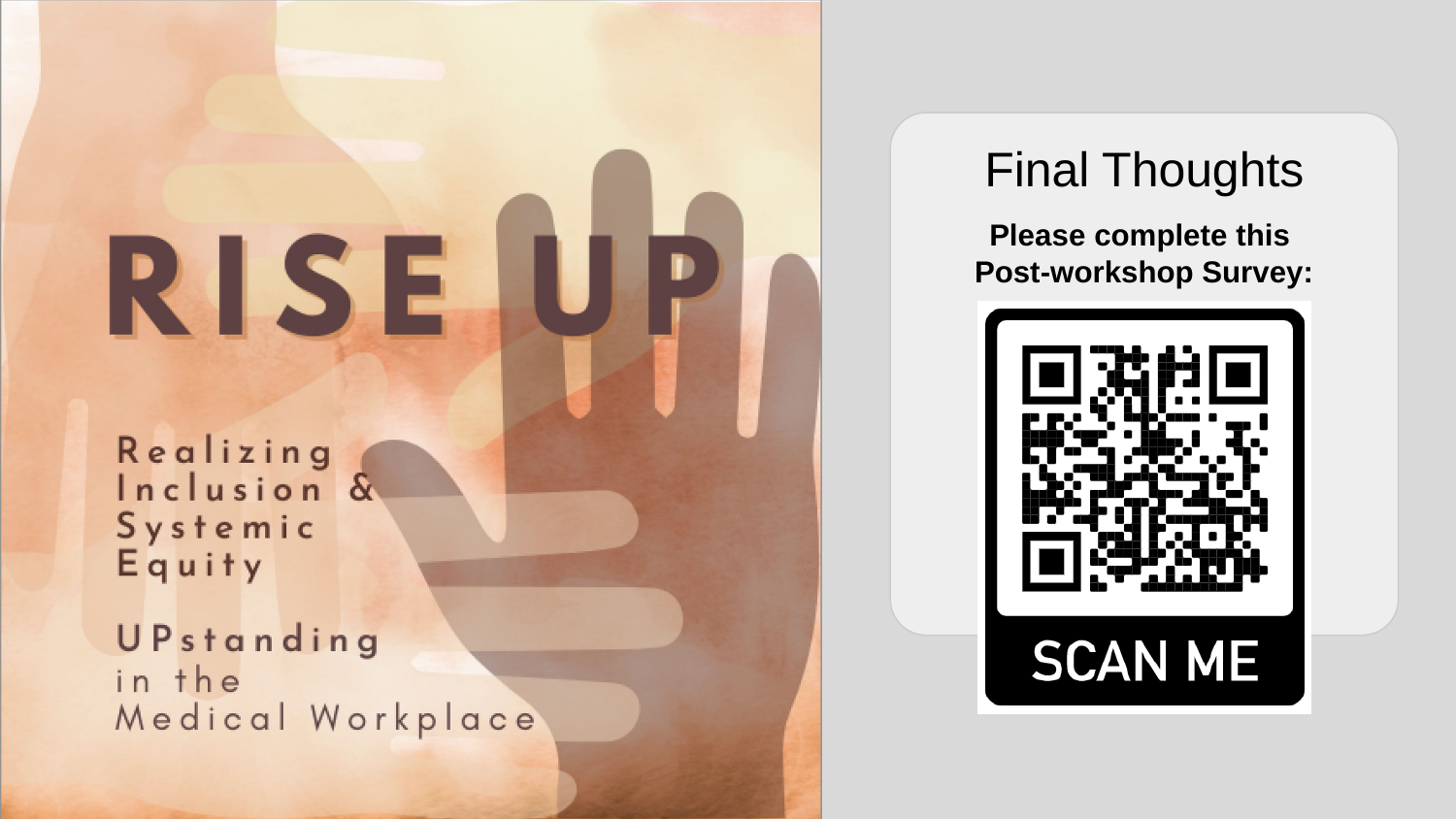

Final Thoughts
Please complete this
Post-workshop Survey:

## Slide 34
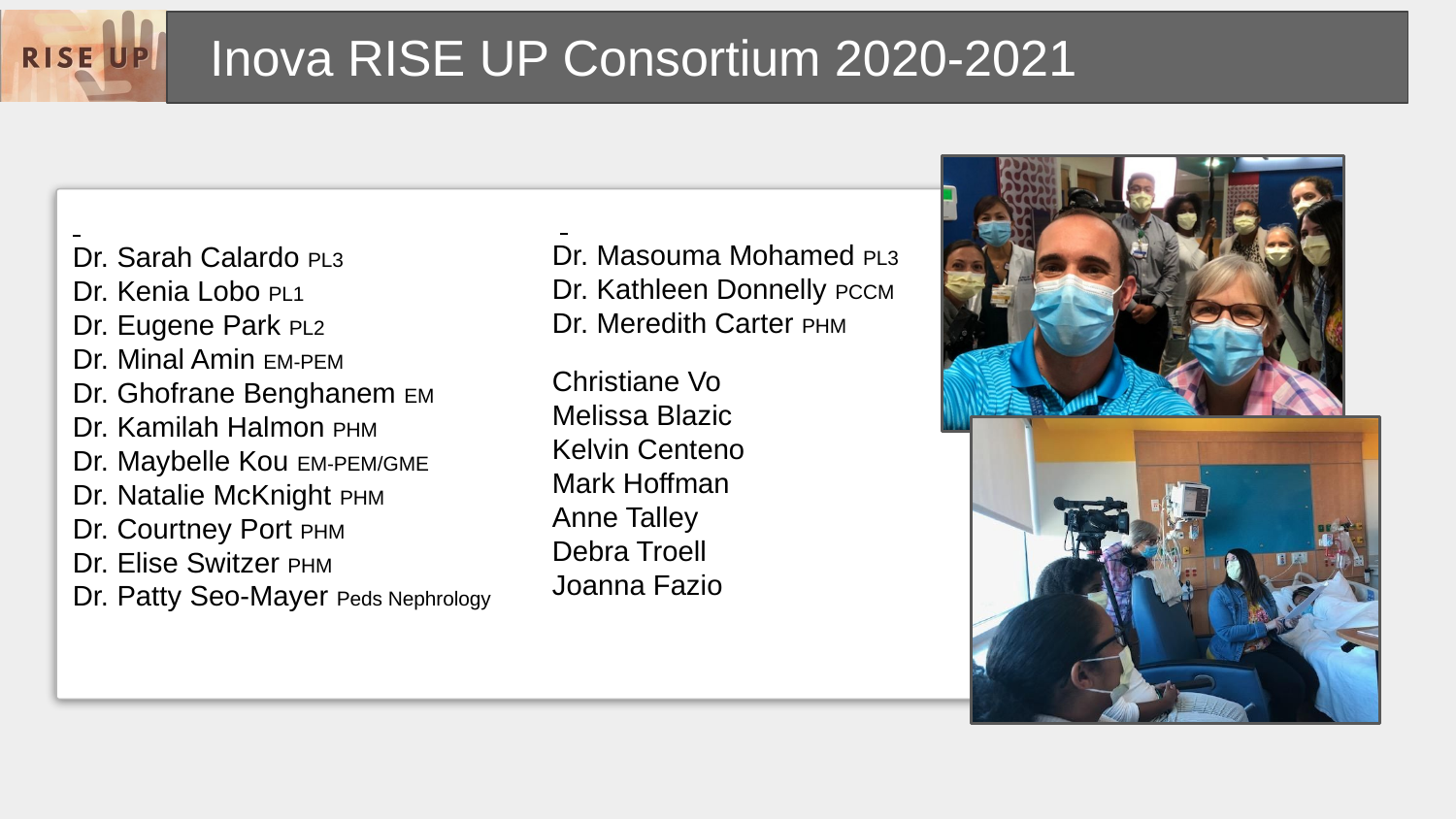

# Inova RISE UP Consortium 2020-2021
Dr. Sarah Calardo PL3
Dr. Kenia Lobo PL1
Dr. Eugene Park PL2
Dr. Minal Amin EM-PEM
Dr. Ghofrane Benghanem EM
Dr. Kamilah Halmon PHM
Dr. Maybelle Kou EM-PEM/GME
Dr. Natalie McKnight PHM
Dr. Courtney Port PHM
Dr. Elise Switzer PHM
Dr. Patty Seo-Mayer Peds Nephrology
Dr. Masouma Mohamed PL3
Dr. Kathleen Donnelly PCCM
Dr. Meredith Carter PHM
Christiane Vo
Melissa Blazic
Kelvin Centeno
Mark Hoffman
Anne Talley
Debra Troell
Joanna Fazio

## Slide 35
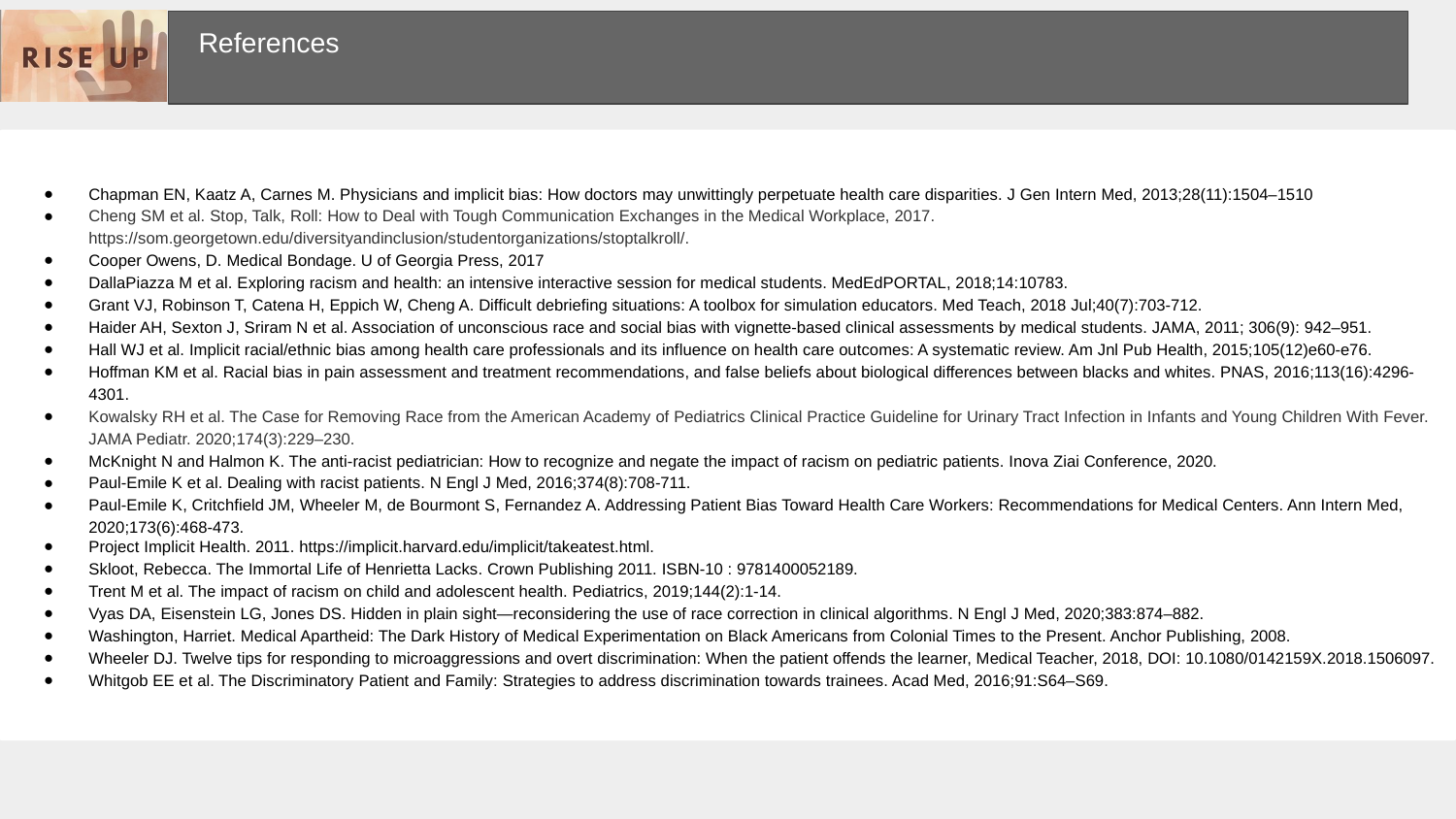

# References
Chapman EN, Kaatz A, Carnes M. Physicians and implicit bias: How doctors may unwittingly perpetuate health care disparities. J Gen Intern Med, 2013;28(11):1504–1510
Cheng SM et al. Stop, Talk, Roll: How to Deal with Tough Communication Exchanges in the Medical Workplace, 2017. https://som.georgetown.edu/diversityandinclusion/studentorganizations/stoptalkroll/.
Cooper Owens, D. Medical Bondage. U of Georgia Press, 2017
DallaPiazza M et al. Exploring racism and health: an intensive interactive session for medical students. MedEdPORTAL, 2018;14:10783.
Grant VJ, Robinson T, Catena H, Eppich W, Cheng A. Difficult debriefing situations: A toolbox for simulation educators. Med Teach, 2018 Jul;40(7):703-712.
Haider AH, Sexton J, Sriram N et al. Association of unconscious race and social bias with vignette-based clinical assessments by medical students. JAMA, 2011; 306(9): 942–951.
Hall WJ et al. Implicit racial/ethnic bias among health care professionals and its influence on health care outcomes: A systematic review. Am Jnl Pub Health, 2015;105(12)e60-e76.
Hoffman KM et al. Racial bias in pain assessment and treatment recommendations, and false beliefs about biological differences between blacks and whites. PNAS, 2016;113(16):4296-4301.
Kowalsky RH et al. The Case for Removing Race from the American Academy of Pediatrics Clinical Practice Guideline for Urinary Tract Infection in Infants and Young Children With Fever. JAMA Pediatr. 2020;174(3):229–230.
McKnight N and Halmon K. The anti-racist pediatrician: How to recognize and negate the impact of racism on pediatric patients. Inova Ziai Conference, 2020.
Paul-Emile K et al. Dealing with racist patients. N Engl J Med, 2016;374(8):708-711.
Paul-Emile K, Critchfield JM, Wheeler M, de Bourmont S, Fernandez A. Addressing Patient Bias Toward Health Care Workers: Recommendations for Medical Centers. Ann Intern Med, 2020;173(6):468-473.
Project Implicit Health. 2011. https://implicit.harvard.edu/implicit/takeatest.html.
Skloot, Rebecca. The Immortal Life of Henrietta Lacks. Crown Publishing 2011. ISBN-10 : 9781400052189.
Trent M et al. The impact of racism on child and adolescent health. Pediatrics, 2019;144(2):1-14.
Vyas DA, Eisenstein LG, Jones DS. Hidden in plain sight—reconsidering the use of race correction in clinical algorithms. N Engl J Med, 2020;383:874–882.
Washington, Harriet. Medical Apartheid: The Dark History of Medical Experimentation on Black Americans from Colonial Times to the Present. Anchor Publishing, 2008.
Wheeler DJ. Twelve tips for responding to microaggressions and overt discrimination: When the patient offends the learner, Medical Teacher, 2018, DOI: 10.1080/0142159X.2018.1506097.
Whitgob EE et al. The Discriminatory Patient and Family: Strategies to address discrimination towards trainees. Acad Med, 2016;91:S64–S69.
