# Supplementary material for: Realizing Inclusion and Systemic Equity in Medicine: Upstanding in the Medical Workplace (RISE UP)—an Antibias Curriculum
Source: MedEdPORTAL. 2022 Apr 6;18:11233. doi: 10.15766/mep_2374-8265.11233 (PMC8983799; doi:10.15766/mep_2374-8265.11233)
Supplement: Supplementary file 1 — Video 1 - The Racist Patient.mp4Video 2 - The Racist Provider.mp4Video 3 - The Racist Consultant.mp4Workshop Slides.pptxFacilitator Guide.pptxPreworkshop Survey.docxPostworkshop Survey.docxSimulation Video Transcripts.docx [file mep_2374-8265.11233-s001.zip › E. Facilitator Guide.pptx]

## Slide 1
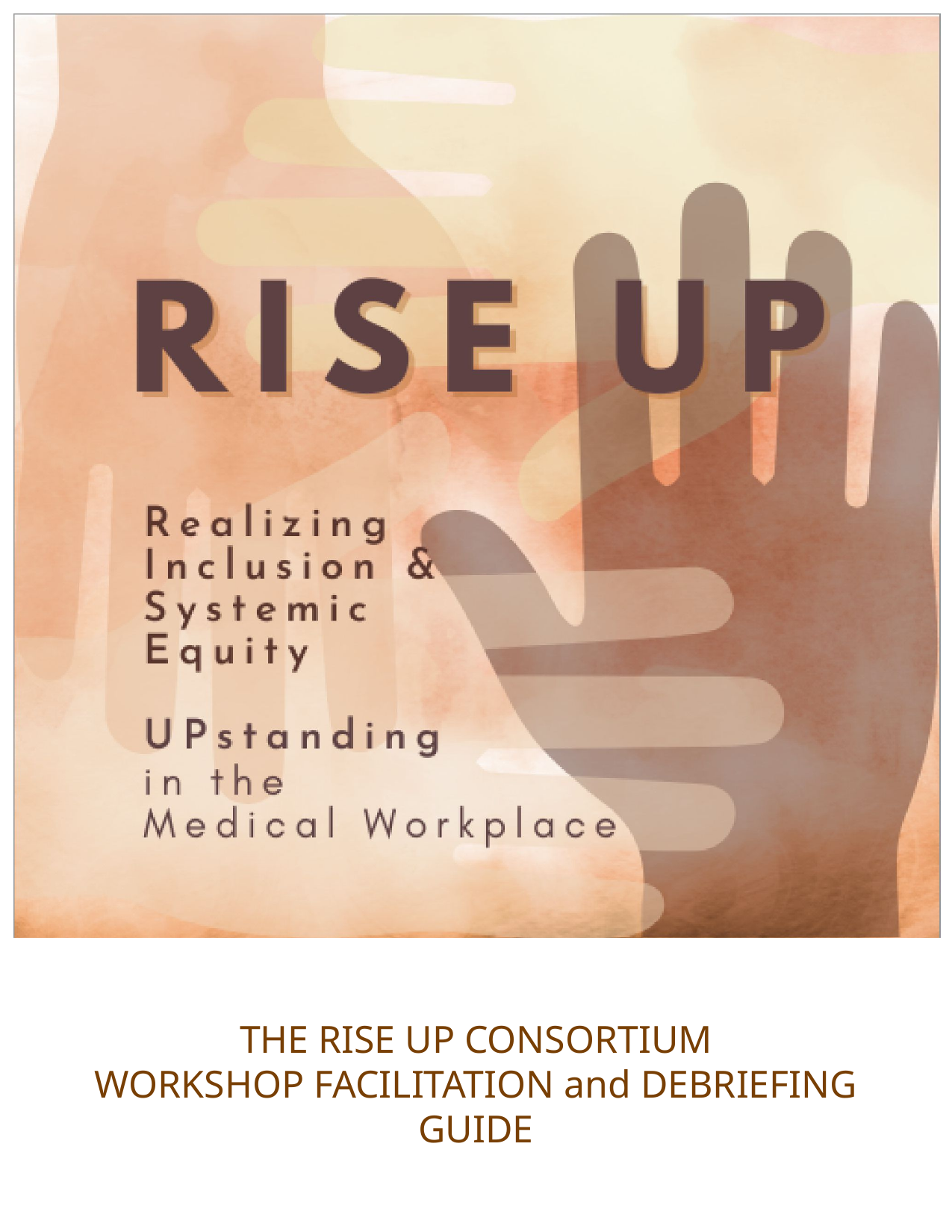

THE RISE UP CONSORTIUM
WORKSHOP FACILITATION and DEBRIEFING GUIDE

## Slide 2
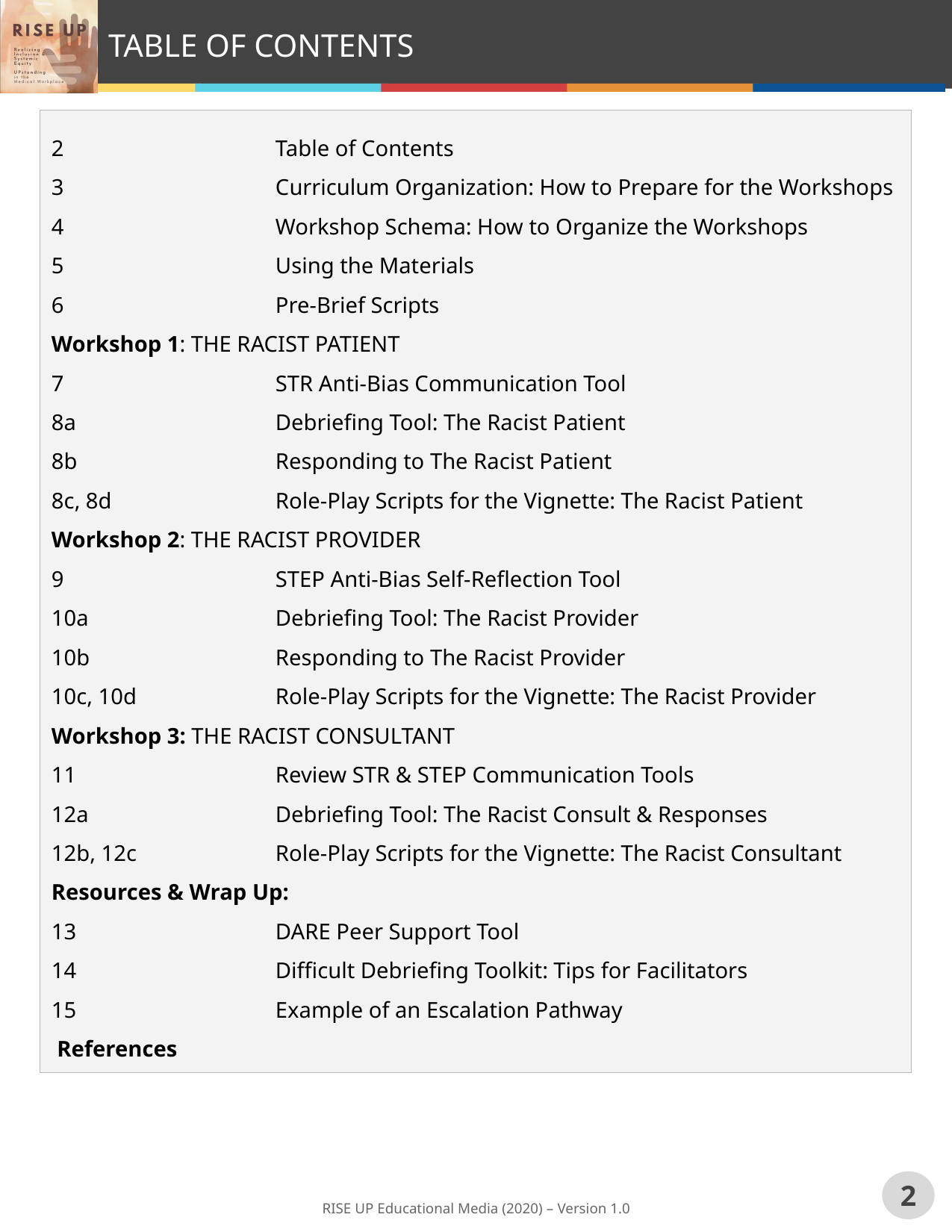

TABLE OF CONTENTS
2 		Table of Contents
3 		Curriculum Organization: How to Prepare for the Workshops
4 		Workshop Schema: How to Organize the Workshops
5 		Using the Materials
6 		Pre-Brief Scripts
Workshop 1: THE RACIST PATIENT
7	 	STR Anti-Bias Communication Tool
8a	 	Debriefing Tool: The Racist Patient
8b 	 	Responding to The Racist Patient
8c, 8d 		Role-Play Scripts for the Vignette: The Racist Patient
Workshop 2: THE RACIST PROVIDER
9 		STEP Anti-Bias Self-Reflection Tool
10a 		Debriefing Tool: The Racist Provider
10b 		Responding to The Racist Provider
10c, 10d 		Role-Play Scripts for the Vignette: The Racist Provider
Workshop 3: THE RACIST CONSULTANT
11		Review STR & STEP Communication Tools
12a 		Debriefing Tool: The Racist Consult & Responses
12b, 12c 		Role-Play Scripts for the Vignette: The Racist Consultant
Resources & Wrap Up:
13 		DARE Peer Support Tool
14		Difficult Debriefing Toolkit: Tips for Facilitators
15 		Example of an Escalation Pathway
 References
2
RISE UP Educational Media (2020) – Version 1.0

## Slide 3
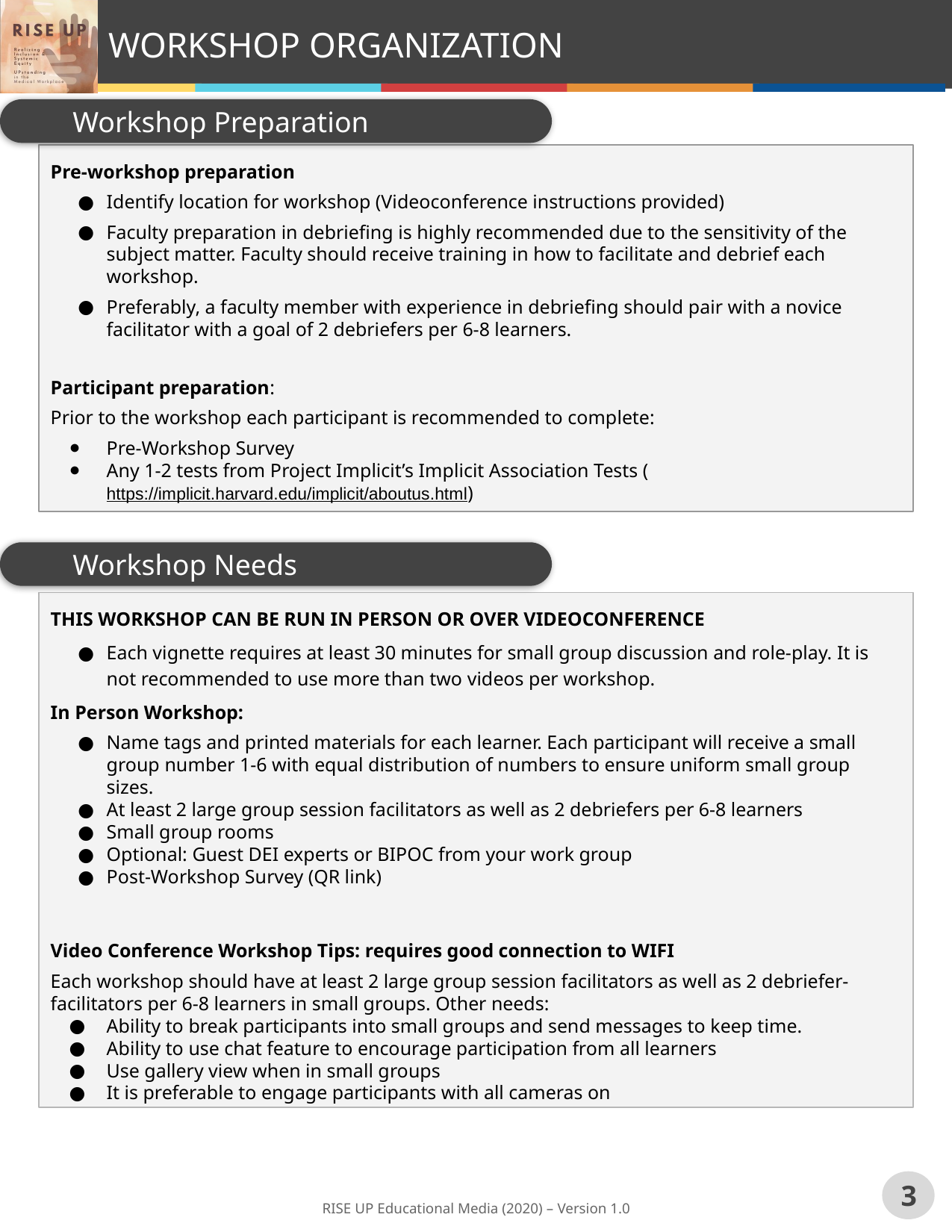

WORKSHOP ORGANIZATION
Workshop Preparation
Pre-workshop preparation
Identify location for workshop (Videoconference instructions provided)
Faculty preparation in debriefing is highly recommended due to the sensitivity of the subject matter. Faculty should receive training in how to facilitate and debrief each workshop.
Preferably, a faculty member with experience in debriefing should pair with a novice facilitator with a goal of 2 debriefers per 6-8 learners.
Participant preparation:
Prior to the workshop each participant is recommended to complete:
Pre-Workshop Survey
Any 1-2 tests from Project Implicit’s Implicit Association Tests (https://implicit.harvard.edu/implicit/aboutus.html)
Workshop Needs
THIS WORKSHOP CAN BE RUN IN PERSON OR OVER VIDEOCONFERENCE
Each vignette requires at least 30 minutes for small group discussion and role-play. It is not recommended to use more than two videos per workshop.
In Person Workshop:
Name tags and printed materials for each learner. Each participant will receive a small group number 1-6 with equal distribution of numbers to ensure uniform small group sizes.
At least 2 large group session facilitators as well as 2 debriefers per 6-8 learners
Small group rooms
Optional: Guest DEI experts or BIPOC from your work group
Post-Workshop Survey (QR link)
Video Conference Workshop Tips: requires good connection to WIFI
Each workshop should have at least 2 large group session facilitators as well as 2 debriefer-facilitators per 6-8 learners in small groups. Other needs:
Ability to break participants into small groups and send messages to keep time.
Ability to use chat feature to encourage participation from all learners
Use gallery view when in small groups
It is preferable to engage participants with all cameras on
3
RISE UP Educational Media (2020) – Version 1.0

## Slide 4
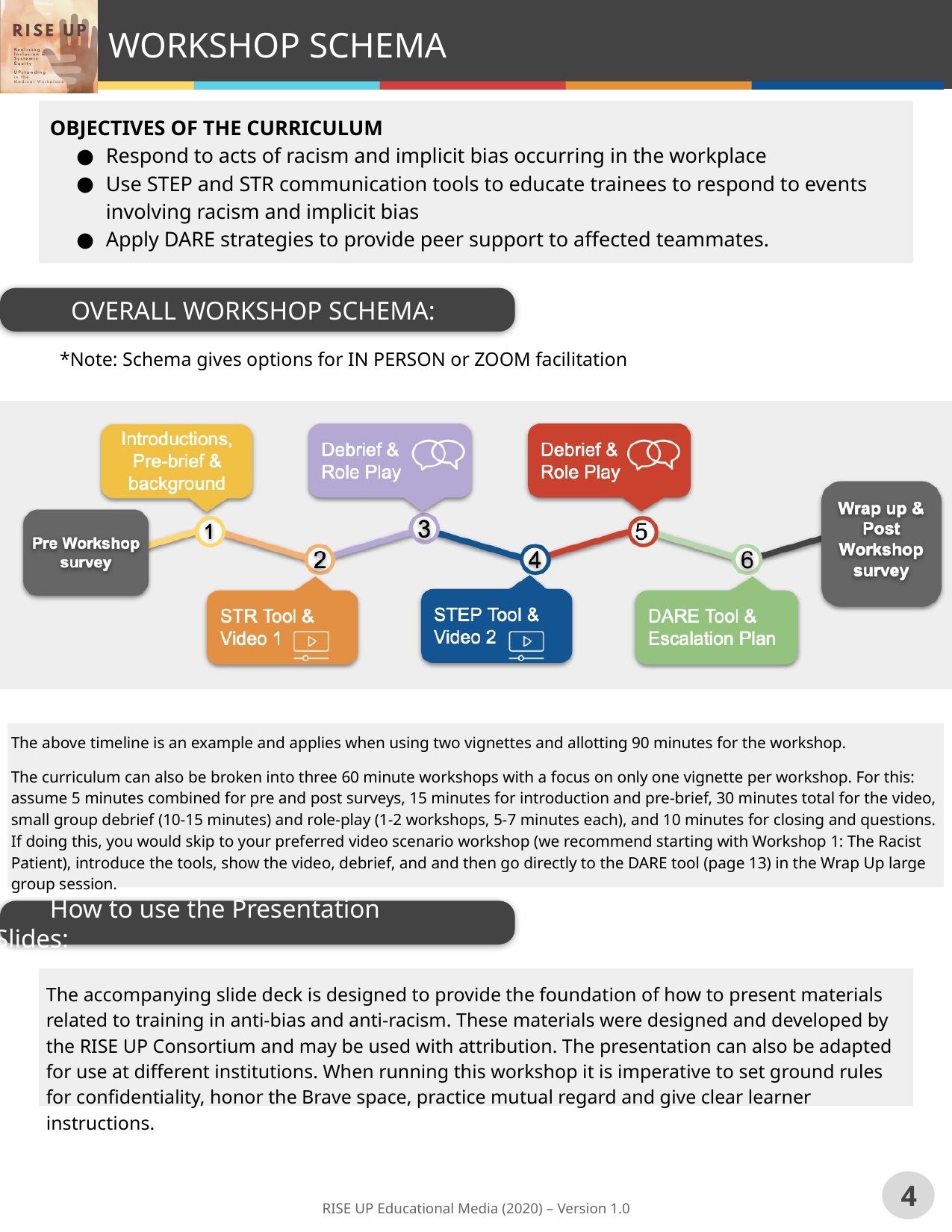

WORKSHOP SCHEMA
Preparation
OBJECTIVES OF THE CURRICULUM
Respond to acts of racism and implicit bias occurring in the workplace
Use STEP and STR communication tools to educate trainees to respond to events involving racism and implicit bias
Apply DARE strategies to provide peer support to affected teammates.
OVERALL WORKSHOP SCHEMA:
*Note: Schema gives options for IN PERSON or ZOOM facilitation
The above timeline is an example and applies when using two vignettes and allotting 90 minutes for the workshop.
The curriculum can also be broken into three 60 minute workshops with a focus on only one vignette per workshop. For this: assume 5 minutes combined for pre and post surveys, 15 minutes for introduction and pre-brief, 30 minutes total for the video, small group debrief (10-15 minutes) and role-play (1-2 workshops, 5-7 minutes each), and 10 minutes for closing and questions. If doing this, you would skip to your preferred video scenario workshop (we recommend starting with Workshop 1: The Racist Patient), introduce the tools, show the video, debrief, and and then go directly to the DARE tool (page 13) in the Wrap Up large group session.
How to use the Presentation Slides:
The accompanying slide deck is designed to provide the foundation of how to present materials related to training in anti-bias and anti-racism. These materials were designed and developed by the RISE UP Consortium and may be used with attribution. The presentation can also be adapted for use at different institutions. When running this workshop it is imperative to set ground rules for confidentiality, honor the Brave space, practice mutual regard and give clear learner instructions.
4
RISE UP Educational Media (2020) – Version 1.0

## Slide 5
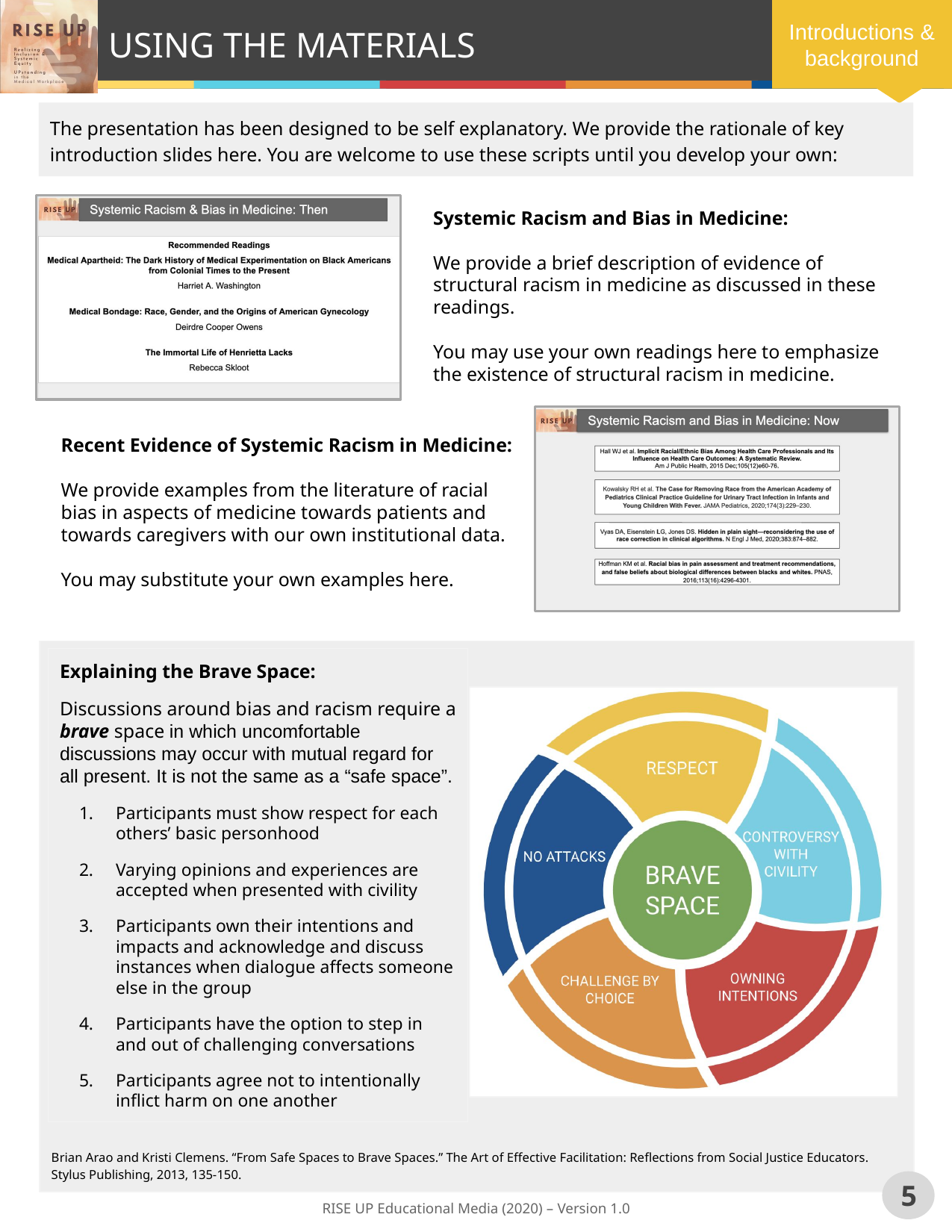

USING THE MATERIALS
Introductions & background
The presentation has been designed to be self explanatory. We provide the rationale of key introduction slides here. You are welcome to use these scripts until you develop your own:
Systemic Racism and Bias in Medicine:
We provide a brief description of evidence of structural racism in medicine as discussed in these readings.
You may use your own readings here to emphasize the existence of structural racism in medicine.
Recent Evidence of Systemic Racism in Medicine:
We provide examples from the literature of racial bias in aspects of medicine towards patients and towards caregivers with our own institutional data.
You may substitute your own examples here.
Brian Arao and Kristi Clemens. “From Safe Spaces to Brave Spaces.” The Art of Effective Facilitation: Reflections from Social Justice Educators. Stylus Publishing, 2013, 135-150.
Explaining the Brave Space:
Discussions around bias and racism require a brave space in which uncomfortable discussions may occur with mutual regard for all present. It is not the same as a “safe space”.
Participants must show respect for each others’ basic personhood
Varying opinions and experiences are accepted when presented with civility
Participants own their intentions and impacts and acknowledge and discuss instances when dialogue affects someone else in the group
Participants have the option to step in and out of challenging conversations
Participants agree not to intentionally inflict harm on one another
Infographic @DrM_Kou
5
RISE UP Educational Media (2020) – Version 1.0

## Slide 6
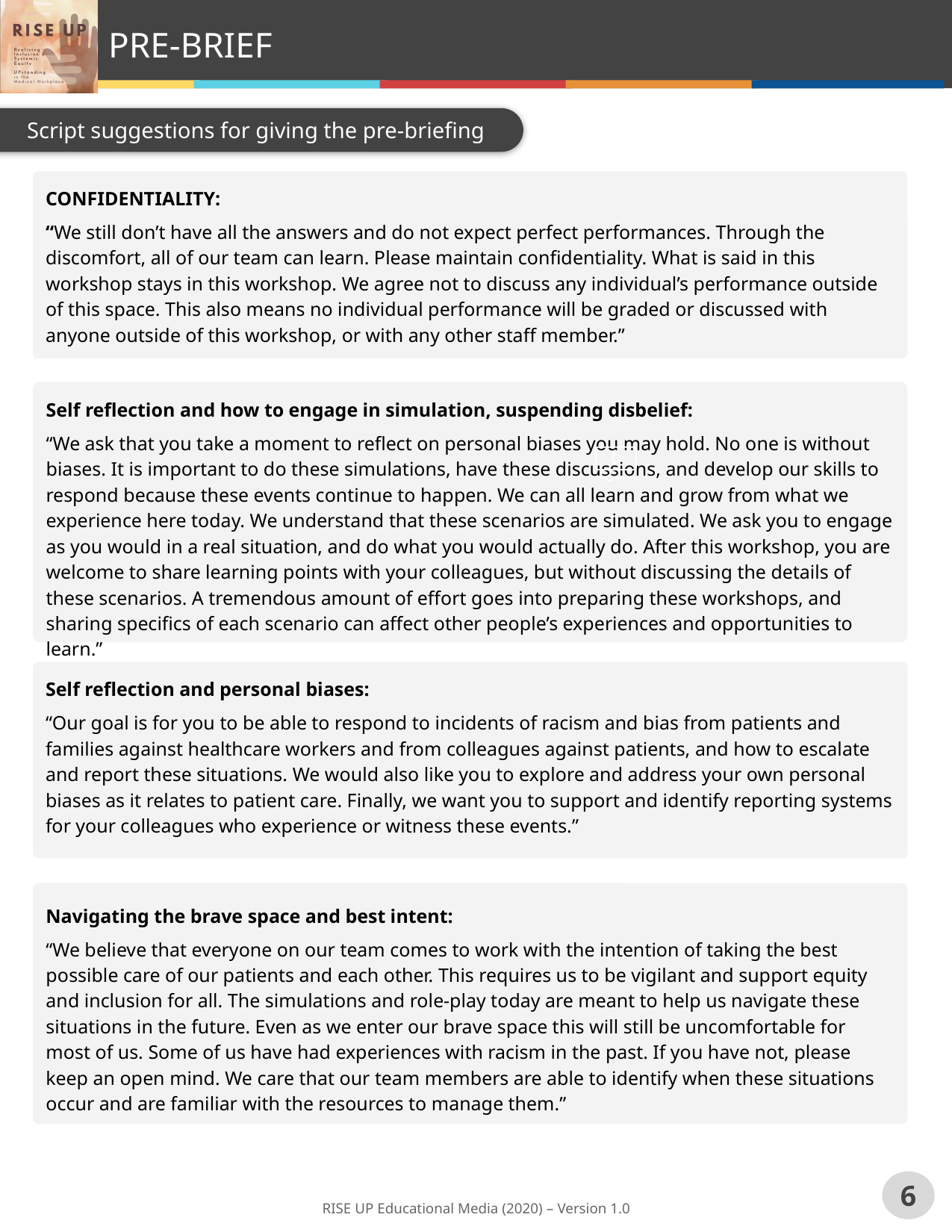

PRE-BRIEF
Script suggestions for giving the pre-briefing
CONFIDENTIALITY:
“We still don’t have all the answers and do not expect perfect performances. Through the discomfort, all of our team can learn. Please maintain confidentiality. What is said in this workshop stays in this workshop. We agree not to discuss any individual’s performance outside of this space. This also means no individual performance will be graded or discussed with anyone outside of this workshop, or with any other staff member.”
Self reflection and how to engage in simulation, suspending disbelief:
“We ask that you take a moment to reflect on personal biases you may hold. No one is without biases. It is important to do these simulations, have these discussions, and develop our skills to respond because these events continue to happen. We can all learn and grow from what we experience here today. We understand that these scenarios are simulated. We ask you to engage as you would in a real situation, and do what you would actually do. After this workshop, you are welcome to share learning points with your colleagues, but without discussing the details of these scenarios. A tremendous amount of effort goes into preparing these workshops, and sharing specifics of each scenario can affect other people’s experiences and opportunities to learn.”
Self reflection and personal biases:
“Our goal is for you to be able to respond to incidents of racism and bias from patients and families against healthcare workers and from colleagues against patients, and how to escalate and report these situations. We would also like you to explore and address your own personal biases as it relates to patient care. Finally, we want you to support and identify reporting systems for your colleagues who experience or witness these events.”
Navigating the brave space and best intent:
“We believe that everyone on our team comes to work with the intention of taking the best possible care of our patients and each other. This requires us to be vigilant and support equity and inclusion for all. The simulations and role-play today are meant to help us navigate these situations in the future. Even as we enter our brave space this will still be uncomfortable for most of us. Some of us have had experiences with racism in the past. If you have not, please keep an open mind. We care that our team members are able to identify when these situations occur and are familiar with the resources to manage them.”
10 min
6
RISE UP Educational Media (2020) – Version 1.0

## Slide 7
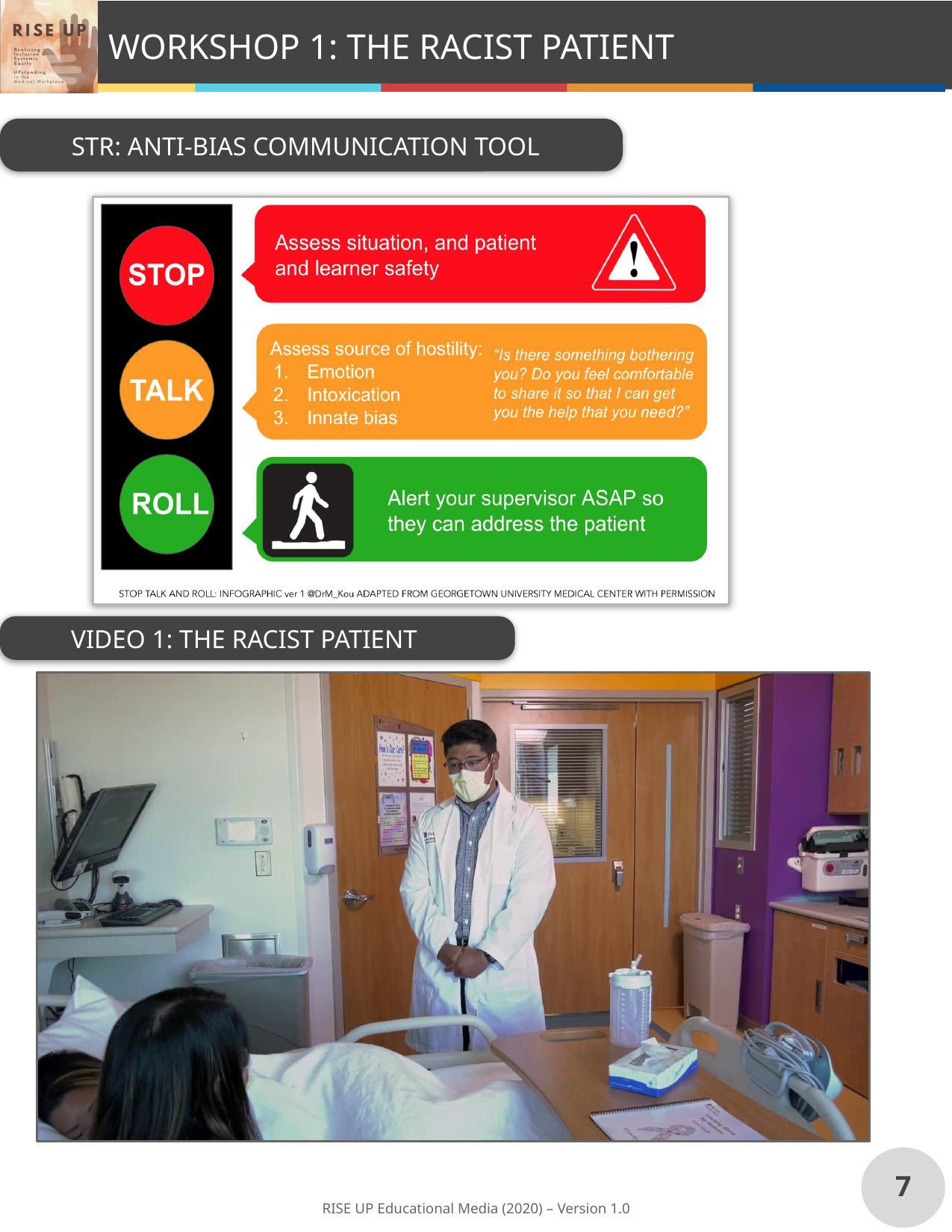

WORKSHOP 1: THE RACIST PATIENT
STR: ANTI-BIAS COMMUNICATION TOOL
VIDEO 1: THE RACIST PATIENT
7
RISE UP Educational Media (2020) – Version 1.0

## Slide 8
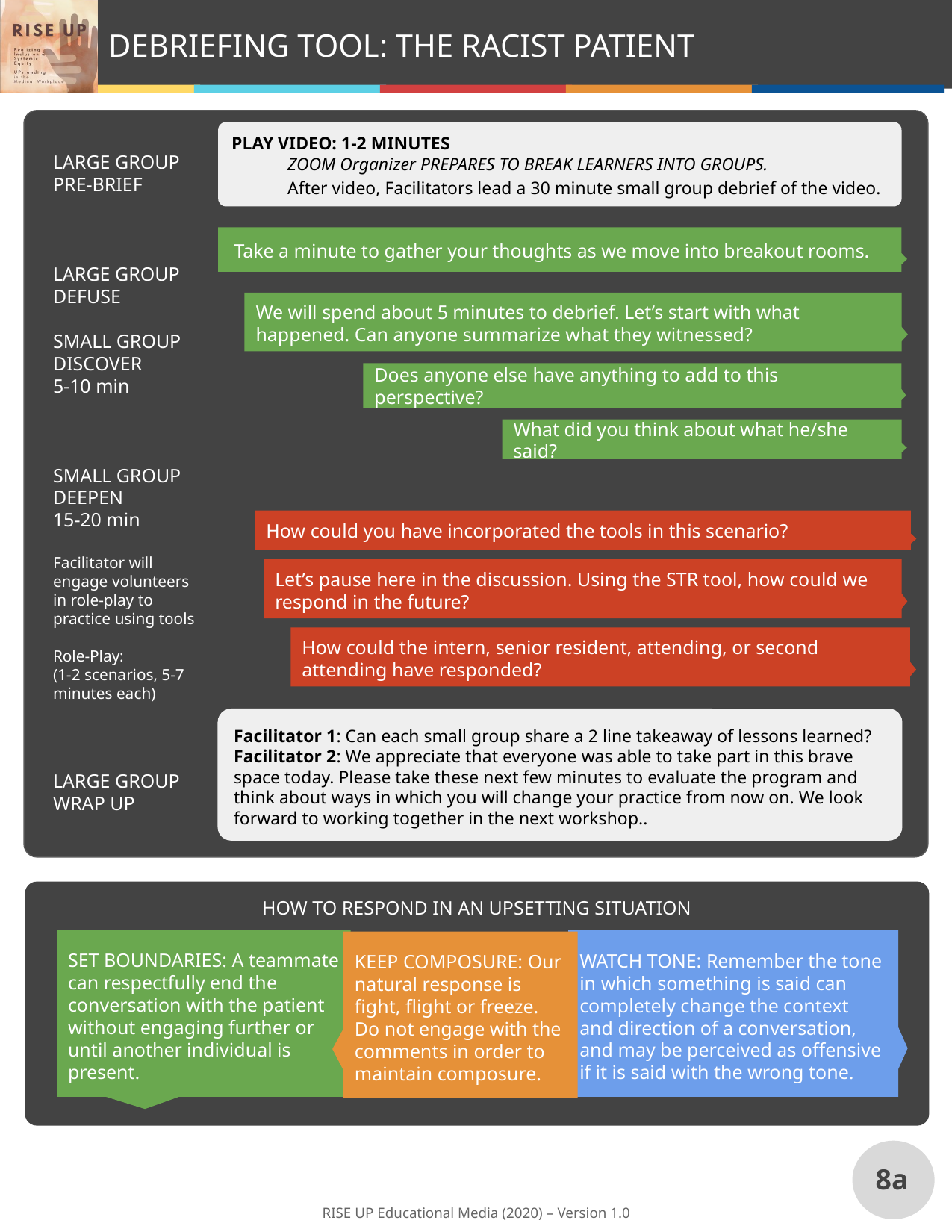

DEBRIEFING TOOL: THE RACIST PATIENT
PLAY VIDEO: 1-2 MINUTES
ZOOM Organizer PREPARES TO BREAK LEARNERS INTO GROUPS.
After video, Facilitators lead a 30 minute small group debrief of the video.
 Take a minute to gather your thoughts as we move into breakout rooms.
We will spend about 5 minutes to debrief. Let’s start with what happened. Can anyone summarize what they witnessed?
Does anyone else have anything to add to this perspective?
What did you think about what he/she said?
How could you have incorporated the tools in this scenario?
Let’s pause here in the discussion. Using the STR tool, how could we respond in the future?
How could the intern, senior resident, attending, or second attending have responded?
Facilitator 1: Can each small group share a 2 line takeaway of lessons learned?
Facilitator 2: We appreciate that everyone was able to take part in this brave space today. Please take these next few minutes to evaluate the program and think about ways in which you will change your practice from now on. We look forward to working together in the next workshop..
LARGE GROUP
PRE-BRIEF
LARGE GROUP
DEFUSE
SMALL GROUP
DISCOVER
5-10 min
SMALL GROUP
DEEPEN
15-20 min
Facilitator will engage volunteers in role-play to practice using tools
Role-Play:
(1-2 scenarios, 5-7 minutes each)
LARGE GROUP
WRAP UP
HOW TO RESPOND IN AN UPSETTING SITUATION
WATCH TONE: Remember the tone in which something is said can completely change the context and direction of a conversation, and may be perceived as offensive if it is said with the wrong tone.
SET BOUNDARIES: A teammate can respectfully end the conversation with the patient without engaging further or until another individual is present.
KEEP COMPOSURE: Our natural response is fight, flight or freeze. Do not engage with the comments in order to maintain composure.
8a
RISE UP Educational Media (2020) – Version 1.0

## Slide 9
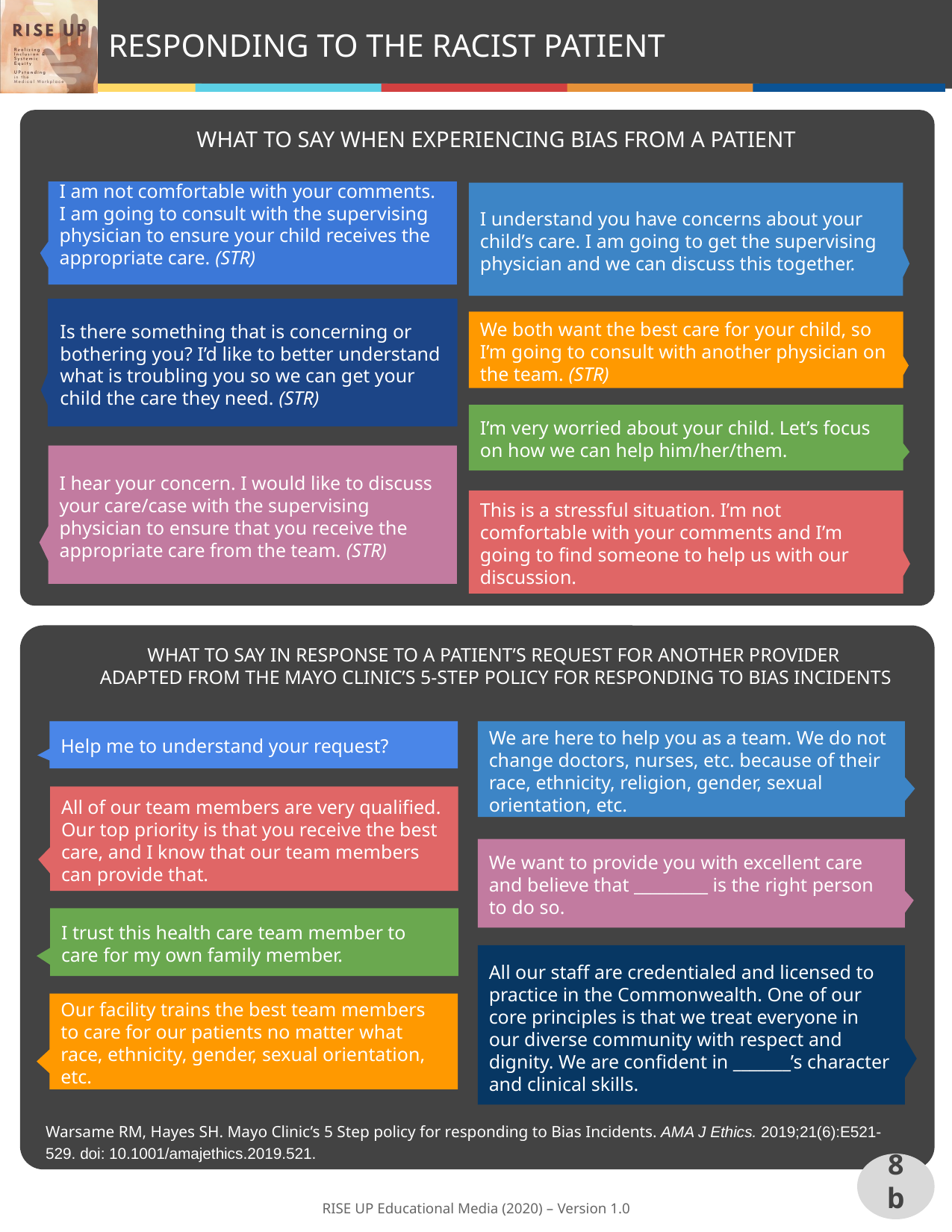

RESPONDING TO THE RACIST PATIENT
WHAT TO SAY WHEN EXPERIENCING BIAS FROM A PATIENT
I am not comfortable with your comments. I am going to consult with the supervising physician to ensure your child receives the appropriate care. (STR)
I understand you have concerns about your child’s care. I am going to get the supervising physician and we can discuss this together.
Is there something that is concerning or bothering you? I’d like to better understand what is troubling you so we can get your child the care they need. (STR)
We both want the best care for your child, so I’m going to consult with another physician on the team. (STR)
I’m very worried about your child. Let’s focus on how we can help him/her/them.
I hear your concern. I would like to discuss your care/case with the supervising physician to ensure that you receive the appropriate care from the team. (STR)
This is a stressful situation. I’m not comfortable with your comments and I’m going to find someone to help us with our discussion.
WHAT TO SAY IN RESPONSE TO A PATIENT’S REQUEST FOR ANOTHER PROVIDER
ADAPTED FROM THE MAYO CLINIC’S 5-STEP POLICY FOR RESPONDING TO BIAS INCIDENTS
We are here to help you as a team. We do not change doctors, nurses, etc. because of their race, ethnicity, religion, gender, sexual orientation, etc.
Help me to understand your request?
All of our team members are very qualified. Our top priority is that you receive the best care, and I know that our team members can provide that.
We want to provide you with excellent care and believe that _________ is the right person to do so.
I trust this health care team member to care for my own family member.
All our staff are credentialed and licensed to practice in the Commonwealth. One of our core principles is that we treat everyone in our diverse community with respect and dignity. We are confident in _______’s character and clinical skills.
Our facility trains the best team members to care for our patients no matter what race, ethnicity, gender, sexual orientation, etc.
Warsame RM, Hayes SH. Mayo Clinic’s 5 Step policy for responding to Bias Incidents. AMA J Ethics. 2019;21(6):E521-529. doi: 10.1001/amajethics.2019.521.
8b
RISE UP Educational Media (2020) – Version 1.0

## Slide 10
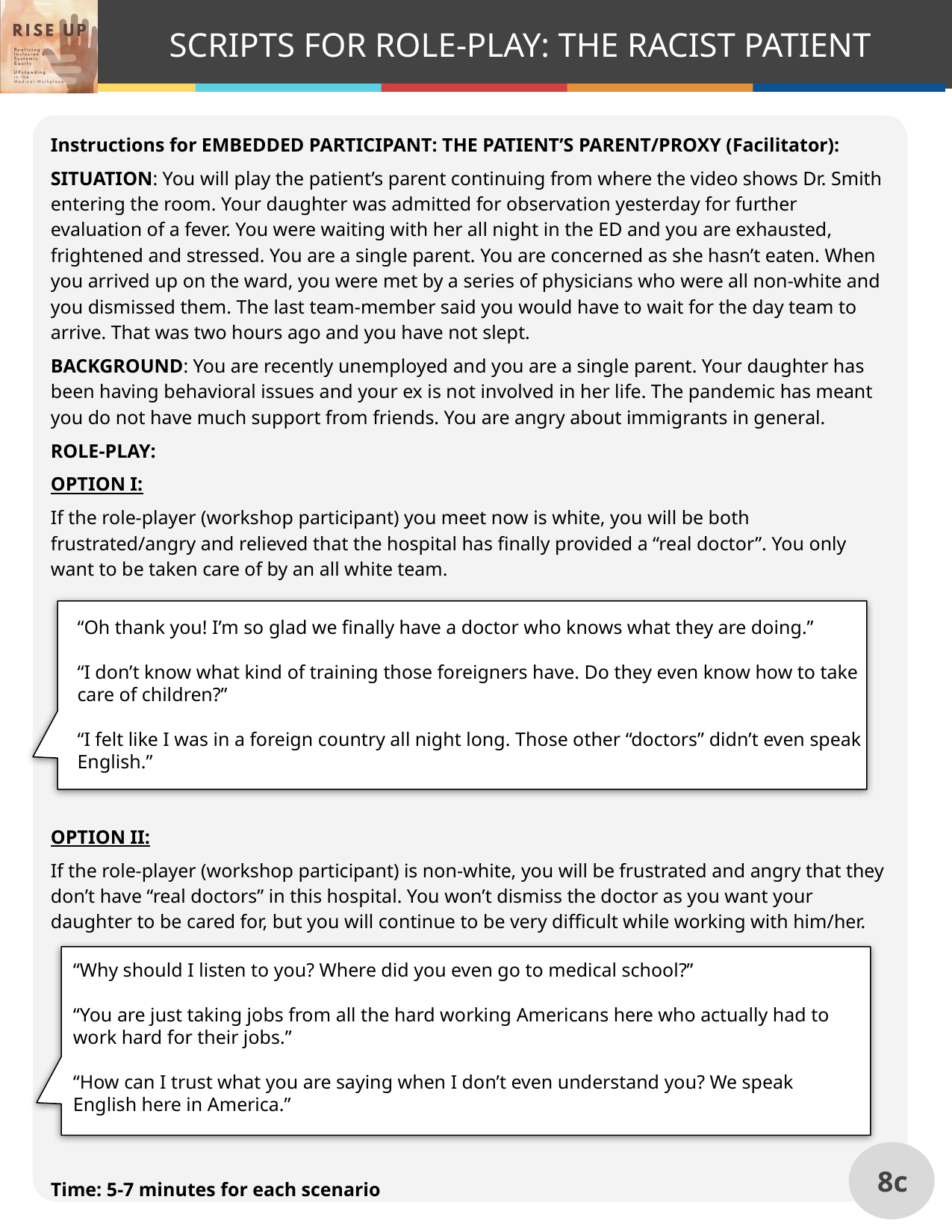

SCRIPTS FOR ROLE-PLAY: THE RACIST PATIENT
Instructions for EMBEDDED PARTICIPANT: THE PATIENT’S PARENT/PROXY (Facilitator):
SITUATION: You will play the patient’s parent continuing from where the video shows Dr. Smith entering the room. Your daughter was admitted for observation yesterday for further evaluation of a fever. You were waiting with her all night in the ED and you are exhausted, frightened and stressed. You are a single parent. You are concerned as she hasn’t eaten. When you arrived up on the ward, you were met by a series of physicians who were all non-white and you dismissed them. The last team-member said you would have to wait for the day team to arrive. That was two hours ago and you have not slept.
BACKGROUND: You are recently unemployed and you are a single parent. Your daughter has been having behavioral issues and your ex is not involved in her life. The pandemic has meant you do not have much support from friends. You are angry about immigrants in general.
ROLE-PLAY:
OPTION I:
If the role-player (workshop participant) you meet now is white, you will be both frustrated/angry and relieved that the hospital has finally provided a “real doctor”. You only want to be taken care of by an all white team.
OPTION II:
If the role-player (workshop participant) is non-white, you will be frustrated and angry that they don’t have “real doctors” in this hospital. You won’t dismiss the doctor as you want your daughter to be cared for, but you will continue to be very difficult while working with him/her.
Time: 5-7 minutes for each scenario
“Oh thank you! I’m so glad we finally have a doctor who knows what they are doing.”
“I don’t know what kind of training those foreigners have. Do they even know how to take care of children?”
“I felt like I was in a foreign country all night long. Those other “doctors” didn’t even speak English.”
“Why should I listen to you? Where did you even go to medical school?”
“You are just taking jobs from all the hard working Americans here who actually had to work hard for their jobs.”
“How can I trust what you are saying when I don’t even understand you? We speak English here in America.”
8c

## Slide 11
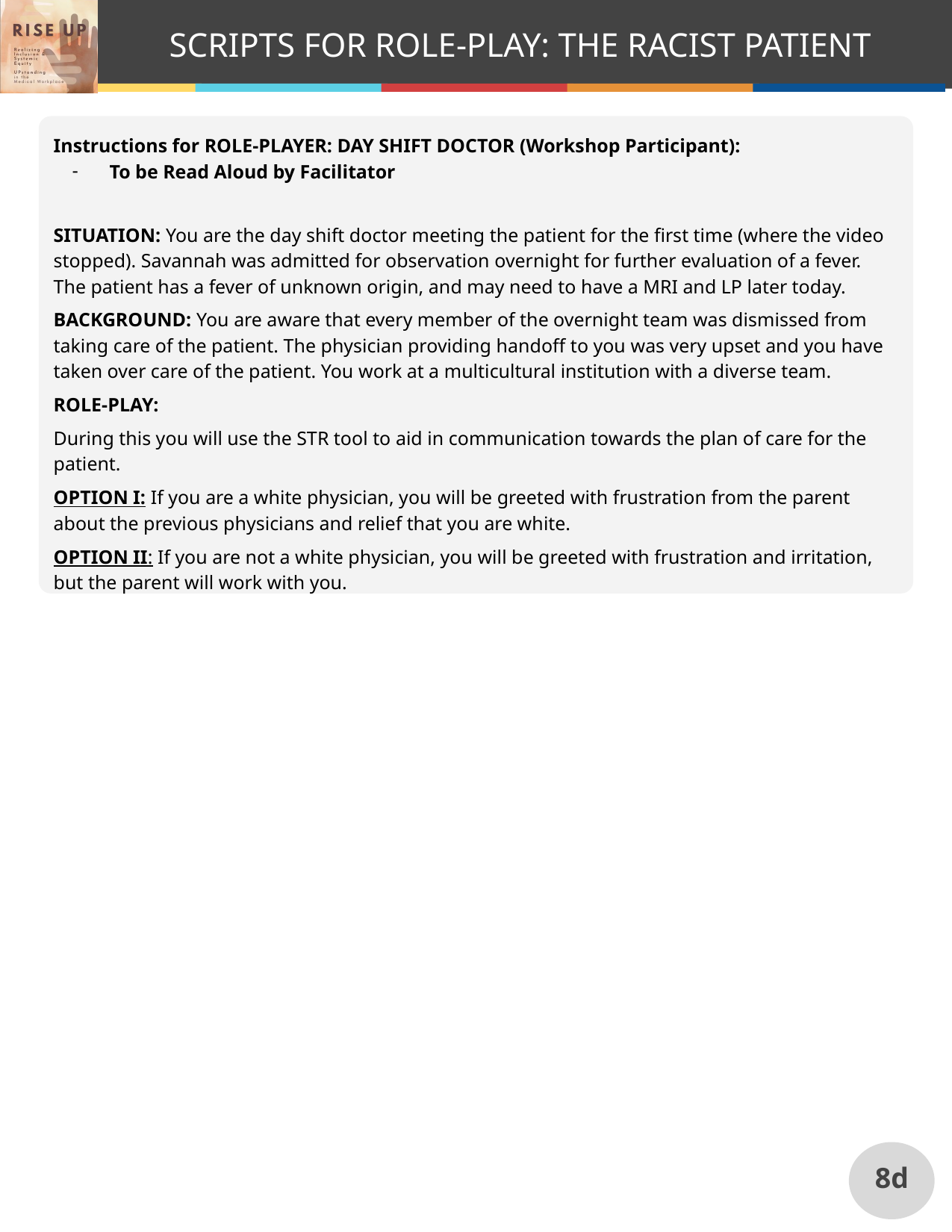

SCRIPTS FOR ROLE-PLAY: THE RACIST PATIENT
Instructions for ROLE-PLAYER: DAY SHIFT DOCTOR (Workshop Participant):
To be Read Aloud by Facilitator
SITUATION: You are the day shift doctor meeting the patient for the first time (where the video stopped). Savannah was admitted for observation overnight for further evaluation of a fever. The patient has a fever of unknown origin, and may need to have a MRI and LP later today.
BACKGROUND: You are aware that every member of the overnight team was dismissed from taking care of the patient. The physician providing handoff to you was very upset and you have taken over care of the patient. You work at a multicultural institution with a diverse team.
ROLE-PLAY:
During this you will use the STR tool to aid in communication towards the plan of care for the patient.
OPTION I: If you are a white physician, you will be greeted with frustration from the parent about the previous physicians and relief that you are white.
OPTION II: If you are not a white physician, you will be greeted with frustration and irritation, but the parent will work with you.
8d

## Slide 12
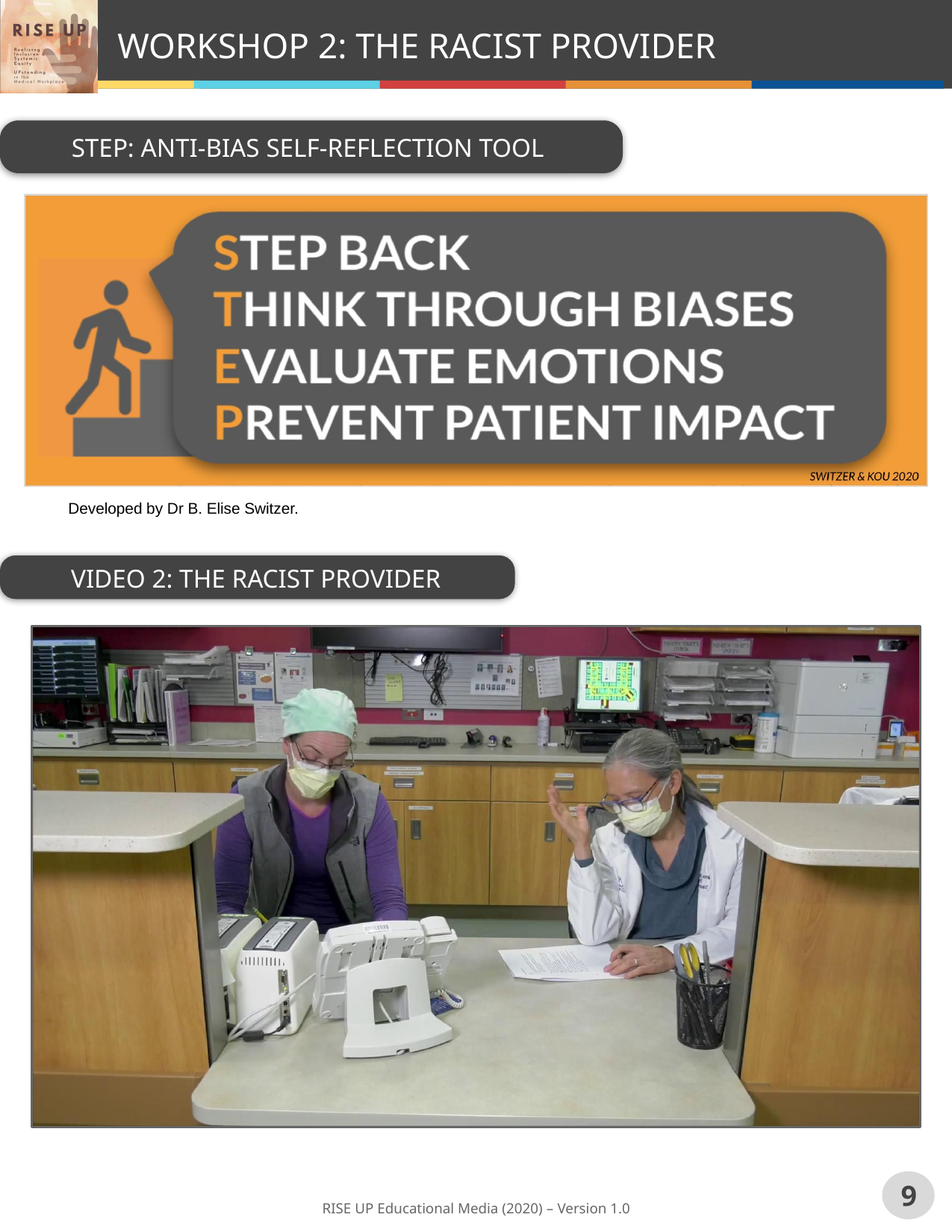

WORKSHOP 2: THE RACIST PROVIDER
STEP: ANTI-BIAS SELF-REFLECTION TOOL
STEP
Developed by Dr B. Elise Switzer.
VIDEO 2: THE RACIST PROVIDER
9
RISE UP Educational Media (2020) – Version 1.0

## Slide 13
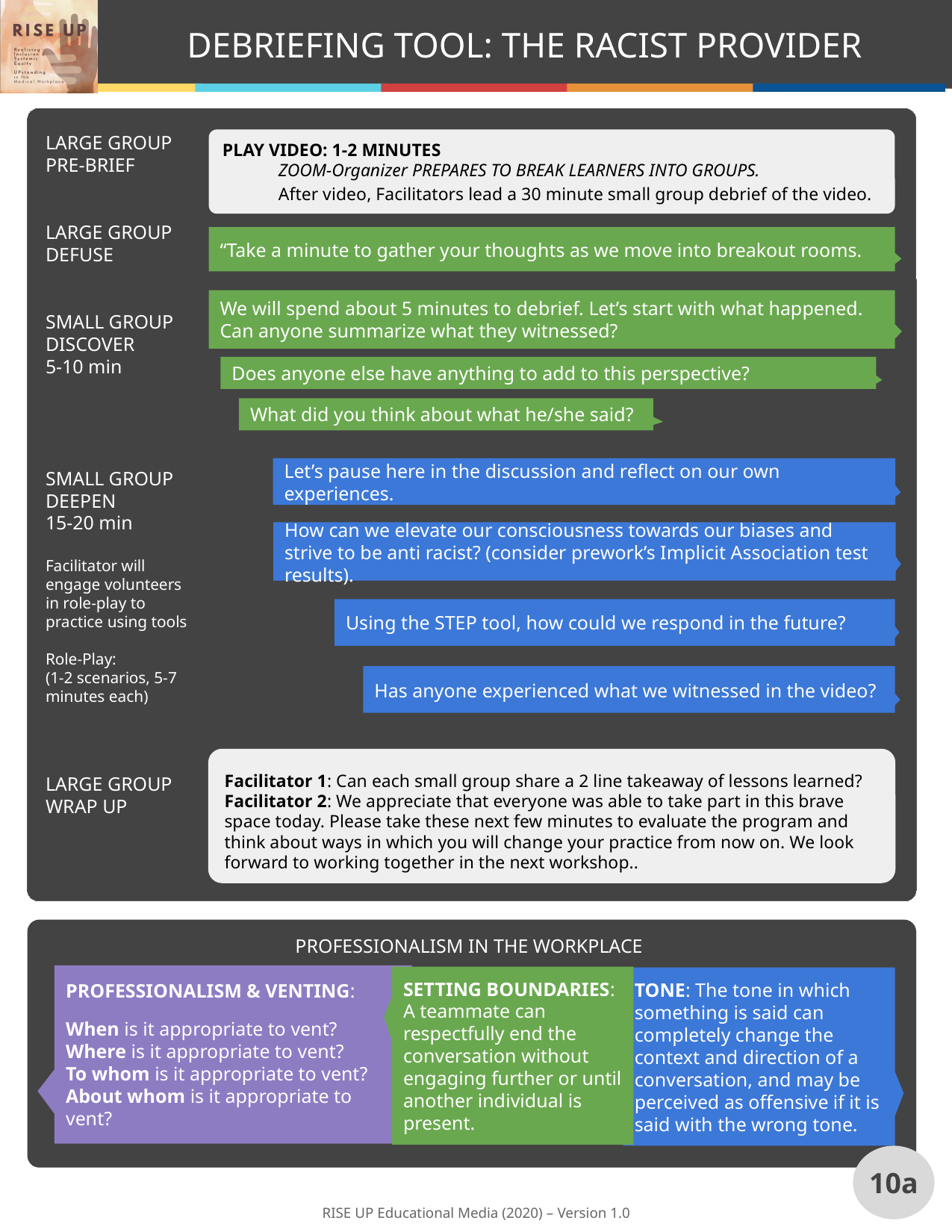

DEBRIEFING TOOL: THE RACIST PROVIDER
LARGE GROUP
PRE-BRIEF
LARGE GROUP
DEFUSE
SMALL GROUP
DISCOVER
5-10 min
SMALL GROUP
DEEPEN
15-20 min
Facilitator will engage volunteers in role-play to practice using tools
Role-Play:
(1-2 scenarios, 5-7 minutes each)
LARGE GROUP
WRAP UP
PLAY VIDEO: 1-2 MINUTES
ZOOM-Organizer PREPARES TO BREAK LEARNERS INTO GROUPS.
After video, Facilitators lead a 30 minute small group debrief of the video.
“Take a minute to gather your thoughts as we move into breakout rooms.
We will spend about 5 minutes to debrief. Let’s start with what happened. Can anyone summarize what they witnessed?
Does anyone else have anything to add to this perspective?
What did you think about what he/she said?
Has anyone experienced what we witnessed in the video?
Facilitator 1: Can each small group share a 2 line takeaway of lessons learned?
Facilitator 2: We appreciate that everyone was able to take part in this brave space today. Please take these next few minutes to evaluate the program and think about ways in which you will change your practice from now on. We look forward to working together in the next workshop..
Let’s pause here in the discussion and reflect on our own experiences.
How can we elevate our consciousness towards our biases and strive to be anti racist? (consider prework’s Implicit Association test results).
Using the STEP tool, how could we respond in the future?
PROFESSIONALISM IN THE WORKPLACE
PROFESSIONALISM & VENTING:
When is it appropriate to vent?
Where is it appropriate to vent?
To whom is it appropriate to vent?
About whom is it appropriate to vent?
TONE: The tone in which something is said can completely change the context and direction of a conversation, and may be perceived as offensive if it is said with the wrong tone.
SETTING BOUNDARIES:
A teammate can respectfully end the conversation without engaging further or until another individual is present.
10a
RISE UP Educational Media (2020) – Version 1.0

## Slide 14
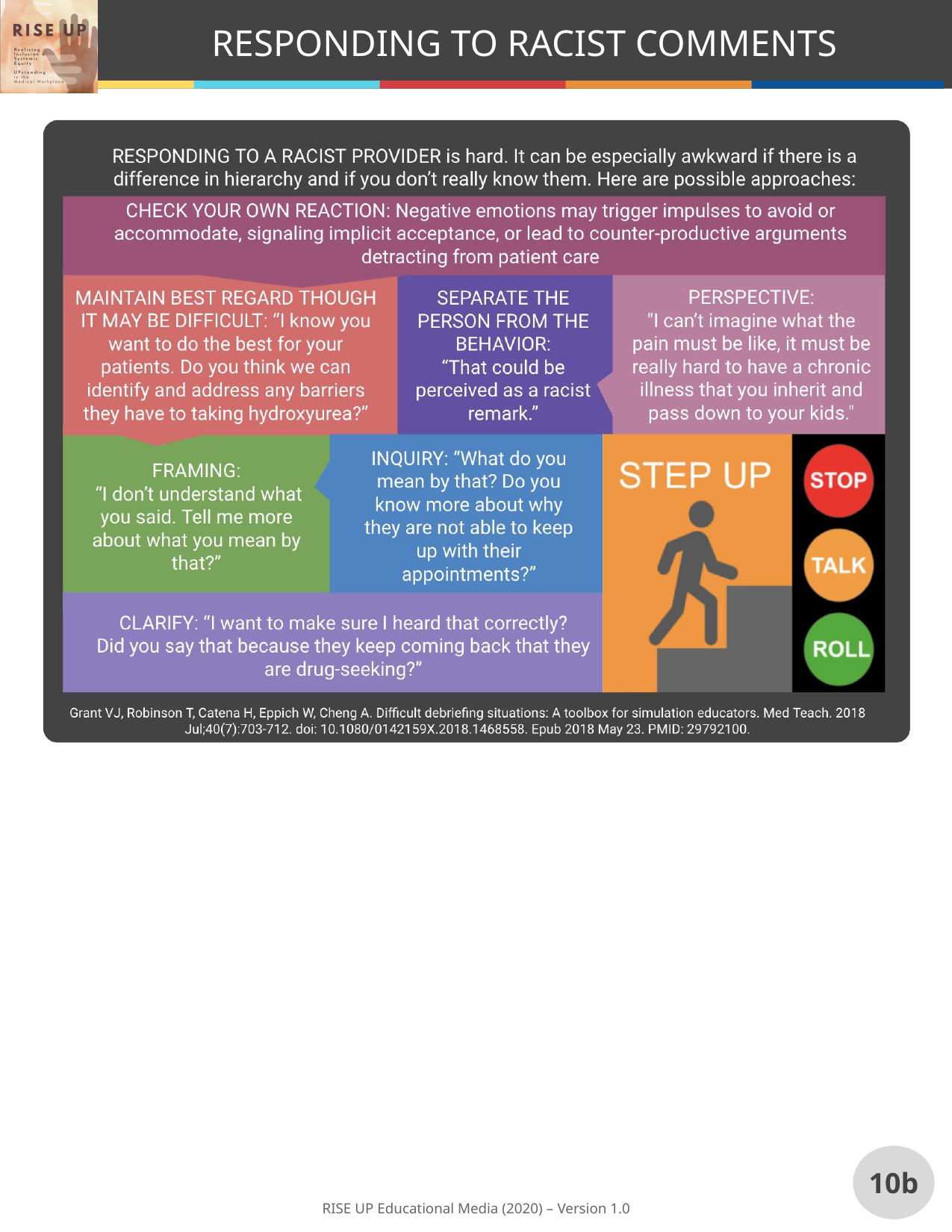

RESPONDING TO RACIST COMMENTS
RESPONDING TO A RACIST PROVIDER is hard. It can be especially awkward if there is a difference in hierarchy and if you don’t really know them. Here are possible approaches:
Grant VJ, Robinson T, Catena H, Eppich W, Cheng A. Difficult debriefing situations: A toolbox for simulation educators. Med Teach. 2018 Jul;40(7):703-712. doi: 10.1080/0142159X.2018.1468558. Epub 2018 May 23. PMID: 29792100.
10b
RISE UP Educational Media (2020) – Version 1.0

## Slide 15
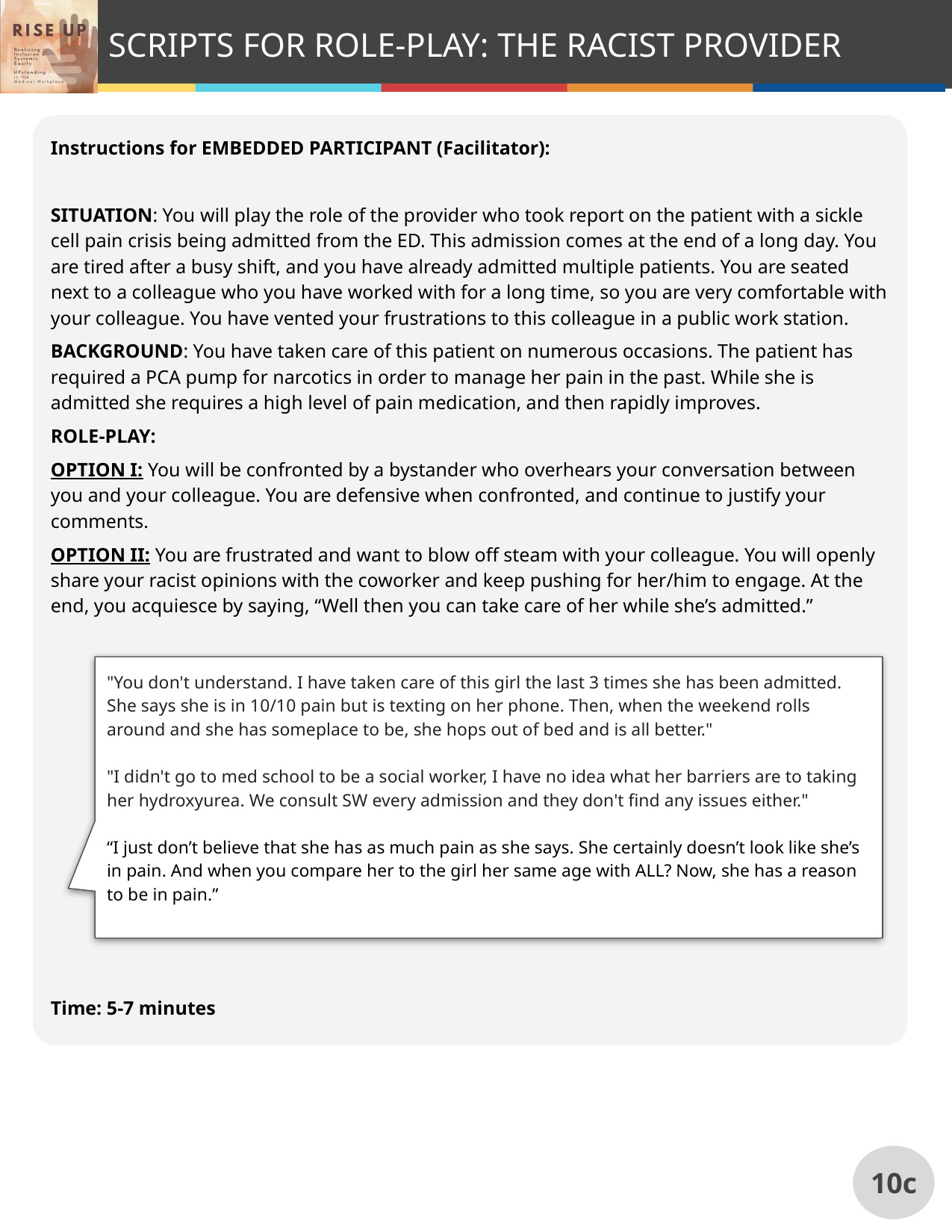

SCRIPTS FOR ROLE-PLAY: THE RACIST PROVIDER
Instructions for EMBEDDED PARTICIPANT (Facilitator):
SITUATION: You will play the role of the provider who took report on the patient with a sickle cell pain crisis being admitted from the ED. This admission comes at the end of a long day. You are tired after a busy shift, and you have already admitted multiple patients. You are seated next to a colleague who you have worked with for a long time, so you are very comfortable with your colleague. You have vented your frustrations to this colleague in a public work station.
BACKGROUND: You have taken care of this patient on numerous occasions. The patient has required a PCA pump for narcotics in order to manage her pain in the past. While she is admitted she requires a high level of pain medication, and then rapidly improves.
ROLE-PLAY:
OPTION I: You will be confronted by a bystander who overhears your conversation between you and your colleague. You are defensive when confronted, and continue to justify your comments.
OPTION II: You are frustrated and want to blow off steam with your colleague. You will openly share your racist opinions with the coworker and keep pushing for her/him to engage. At the end, you acquiesce by saying, “Well then you can take care of her while she’s admitted.”
Time: 5-7 minutes
"You don't understand. I have taken care of this girl the last 3 times she has been admitted. She says she is in 10/10 pain but is texting on her phone. Then, when the weekend rolls around and she has someplace to be, she hops out of bed and is all better."
"I didn't go to med school to be a social worker, I have no idea what her barriers are to taking her hydroxyurea. We consult SW every admission and they don't find any issues either."
“I just don’t believe that she has as much pain as she says. She certainly doesn’t look like she’s in pain. And when you compare her to the girl her same age with ALL? Now, she has a reason to be in pain.”
10c

## Slide 16
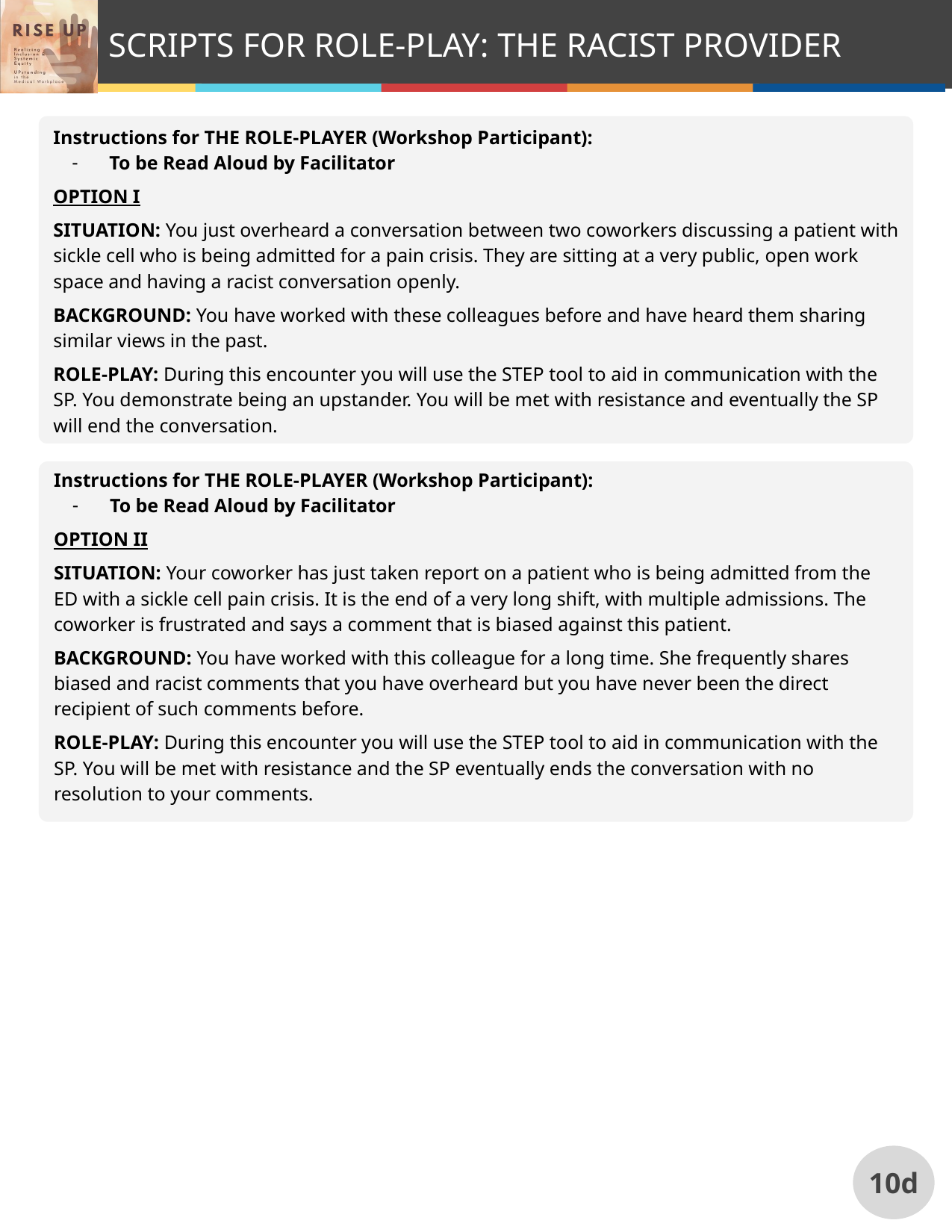

SCRIPTS FOR ROLE-PLAY: THE RACIST PROVIDER
Instructions for THE ROLE-PLAYER (Workshop Participant):
To be Read Aloud by Facilitator
OPTION I
SITUATION: You just overheard a conversation between two coworkers discussing a patient with sickle cell who is being admitted for a pain crisis. They are sitting at a very public, open work space and having a racist conversation openly.
BACKGROUND: You have worked with these colleagues before and have heard them sharing similar views in the past.
ROLE-PLAY: During this encounter you will use the STEP tool to aid in communication with the SP. You demonstrate being an upstander. You will be met with resistance and eventually the SP will end the conversation.
Instructions for THE ROLE-PLAYER (Workshop Participant):
To be Read Aloud by Facilitator
OPTION II
SITUATION: Your coworker has just taken report on a patient who is being admitted from the ED with a sickle cell pain crisis. It is the end of a very long shift, with multiple admissions. The coworker is frustrated and says a comment that is biased against this patient.
BACKGROUND: You have worked with this colleague for a long time. She frequently shares biased and racist comments that you have overheard but you have never been the direct recipient of such comments before.
ROLE-PLAY: During this encounter you will use the STEP tool to aid in communication with the SP. You will be met with resistance and the SP eventually ends the conversation with no resolution to your comments.
10d

## Slide 17
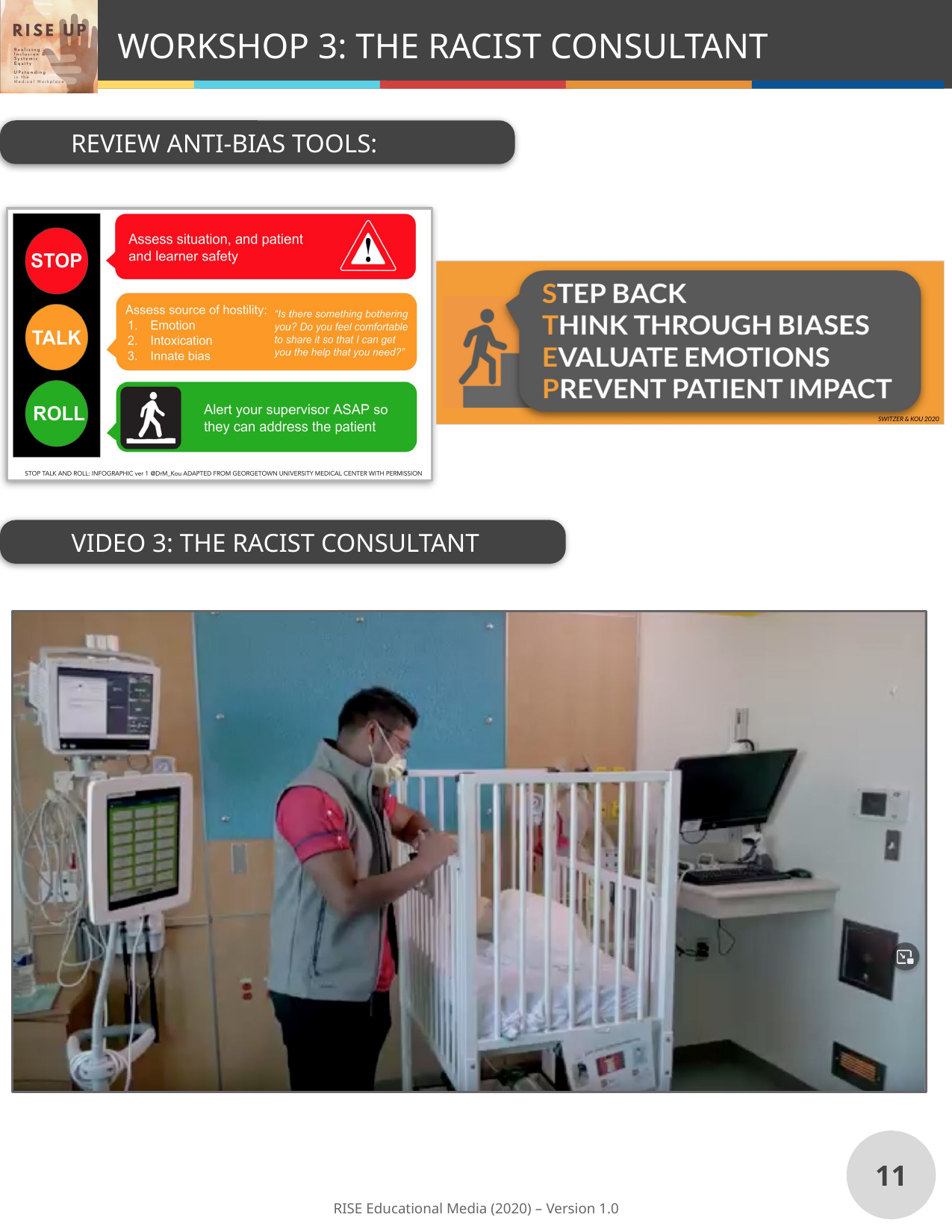

WORKSHOP 3: THE RACIST CONSULTANT
REVIEW ANTI-BIAS TOOLS:
STEP
VIDEO 3: THE RACIST CONSULTANT
11
RISE Educational Media (2020) – Version 1.0

## Slide 18
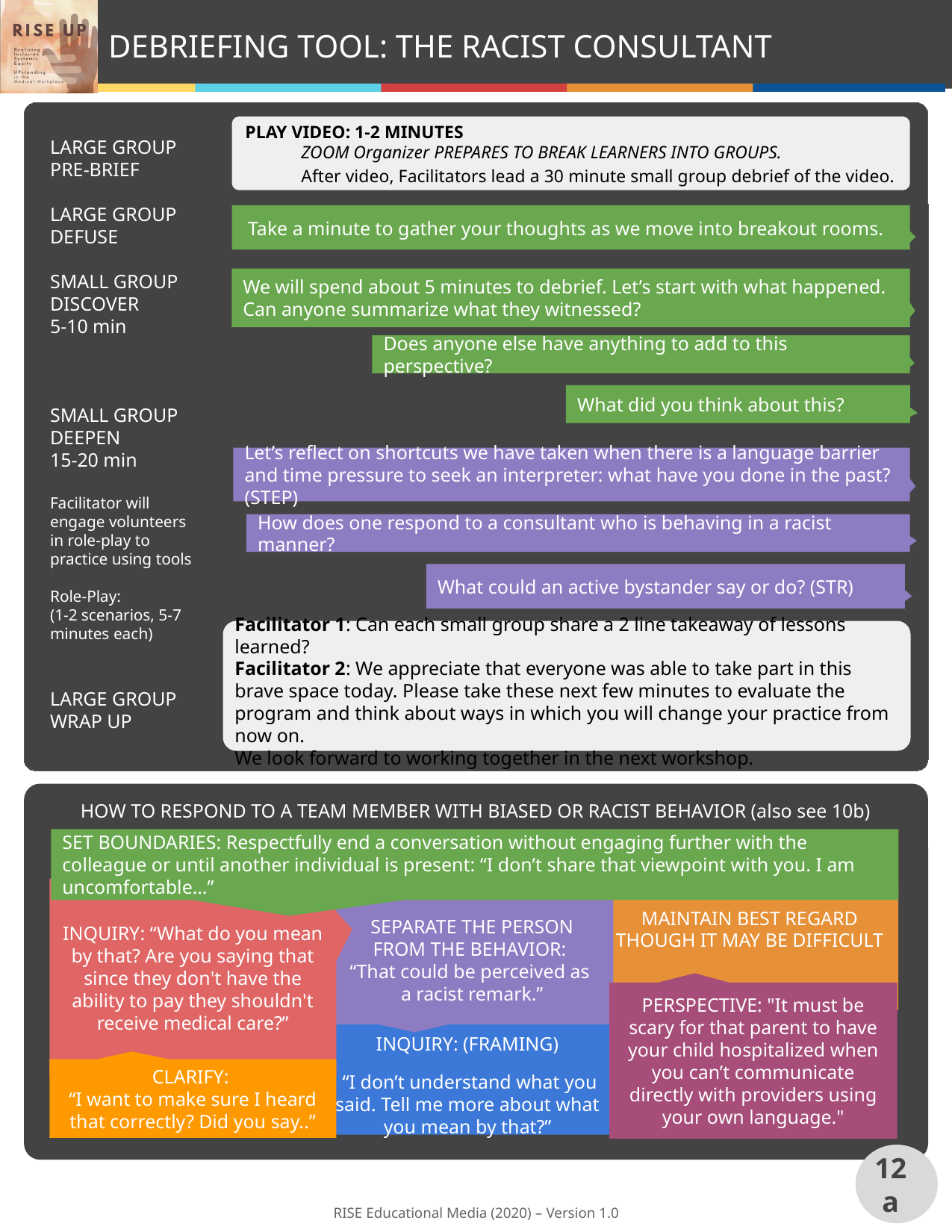

DEBRIEFING TOOL: THE RACIST CONSULTANT
PLAY VIDEO: 1-2 MINUTES
ZOOM Organizer PREPARES TO BREAK LEARNERS INTO GROUPS.
After video, Facilitators lead a 30 minute small group debrief of the video.
 Take a minute to gather your thoughts as we move into breakout rooms.
We will spend about 5 minutes to debrief. Let’s start with what happened.
Can anyone summarize what they witnessed?
Does anyone else have anything to add to this perspective?
What did you think about this?
Let’s reflect on shortcuts we have taken when there is a language barrier and time pressure to seek an interpreter: what have you done in the past? (STEP)
How does one respond to a consultant who is behaving in a racist manner?
What could an active bystander say or do? (STR)
Facilitator 1: Can each small group share a 2 line takeaway of lessons learned?
Facilitator 2: We appreciate that everyone was able to take part in this brave space today. Please take these next few minutes to evaluate the program and think about ways in which you will change your practice from now on.
We look forward to working together in the next workshop.
LARGE GROUP
PRE-BRIEF
LARGE GROUP
DEFUSE
SMALL GROUP
DISCOVER
5-10 min
SMALL GROUP
DEEPEN
15-20 min
Facilitator will engage volunteers in role-play to practice using tools
Role-Play:
(1-2 scenarios, 5-7 minutes each)
LARGE GROUP
WRAP UP
HOW TO RESPOND TO A TEAM MEMBER WITH BIASED OR RACIST BEHAVIOR (also see 10b)
SET BOUNDARIES: Respectfully end a conversation without engaging further with the colleague or until another individual is present: “I don’t share that viewpoint with you. I am uncomfortable…”
INQUIRY: “What do you mean by that? Are you saying that since they don't have the ability to pay they shouldn't receive medical care?”
SEPARATE THE PERSON FROM THE BEHAVIOR:
“That could be perceived as
a racist remark.”
PERSPECTIVE: "It must be scary for that parent to have your child hospitalized when you can’t communicate directly with providers using your own language."
INQUIRY: (FRAMING)
 “I don’t understand what you said. Tell me more about what you mean by that?”
CLARIFY:
“I want to make sure I heard that correctly? Did you say..”
MAINTAIN BEST REGARD THOUGH IT MAY BE DIFFICULT
12a
RISE Educational Media (2020) – Version 1.0

## Slide 19
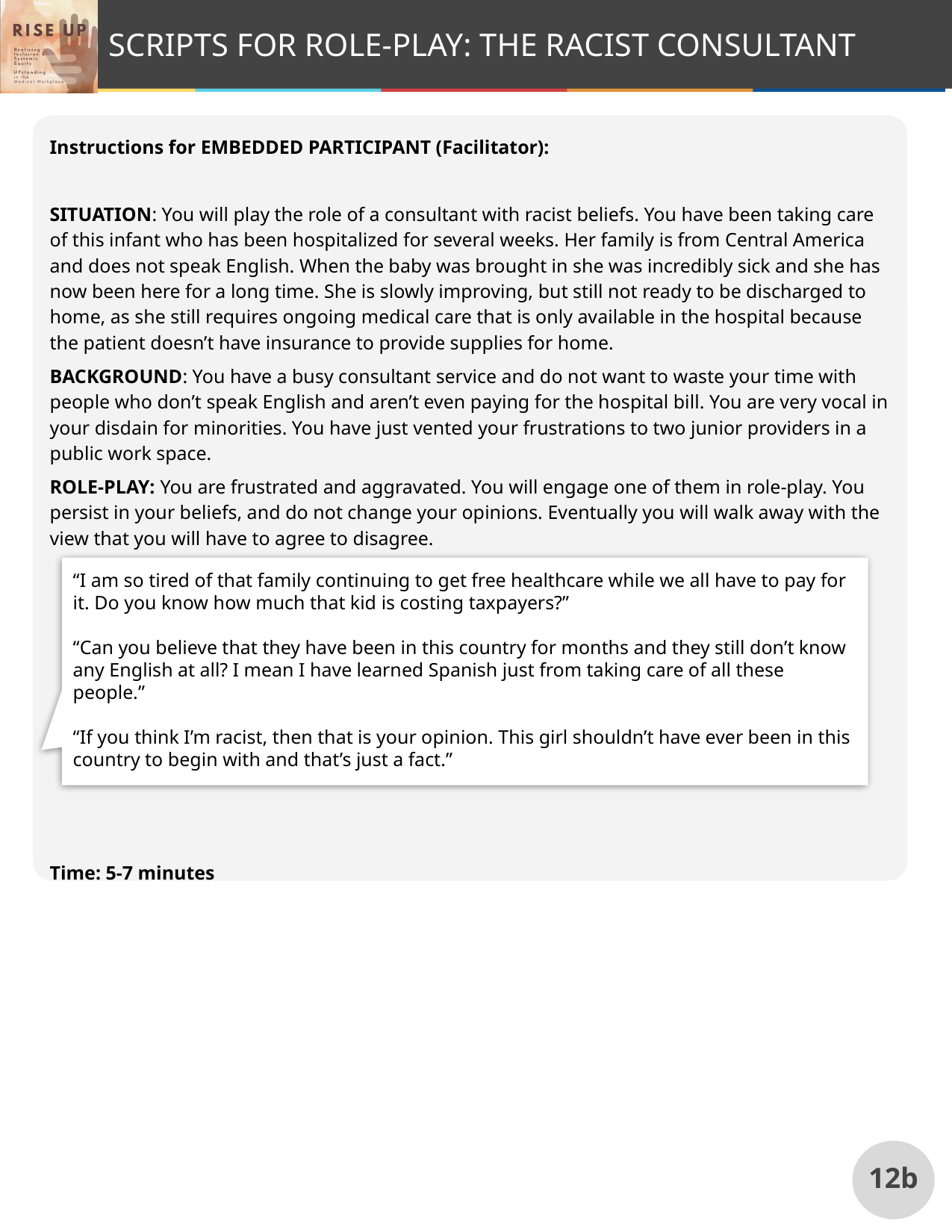

SCRIPTS FOR ROLE-PLAY: THE RACIST CONSULTANT
Instructions for EMBEDDED PARTICIPANT (Facilitator):
SITUATION: You will play the role of a consultant with racist beliefs. You have been taking care of this infant who has been hospitalized for several weeks. Her family is from Central America and does not speak English. When the baby was brought in she was incredibly sick and she has now been here for a long time. She is slowly improving, but still not ready to be discharged to home, as she still requires ongoing medical care that is only available in the hospital because the patient doesn’t have insurance to provide supplies for home.
BACKGROUND: You have a busy consultant service and do not want to waste your time with people who don’t speak English and aren’t even paying for the hospital bill. You are very vocal in your disdain for minorities. You have just vented your frustrations to two junior providers in a public work space.
ROLE-PLAY: You are frustrated and aggravated. You will engage one of them in role-play. You persist in your beliefs, and do not change your opinions. Eventually you will walk away with the view that you will have to agree to disagree.
Time: 5-7 minutes
“I am so tired of that family continuing to get free healthcare while we all have to pay for it. Do you know how much that kid is costing taxpayers?”
“Can you believe that they have been in this country for months and they still don’t know any English at all? I mean I have learned Spanish just from taking care of all these people.”
“If you think I’m racist, then that is your opinion. This girl shouldn’t have ever been in this country to begin with and that’s just a fact.”
12b

## Slide 20
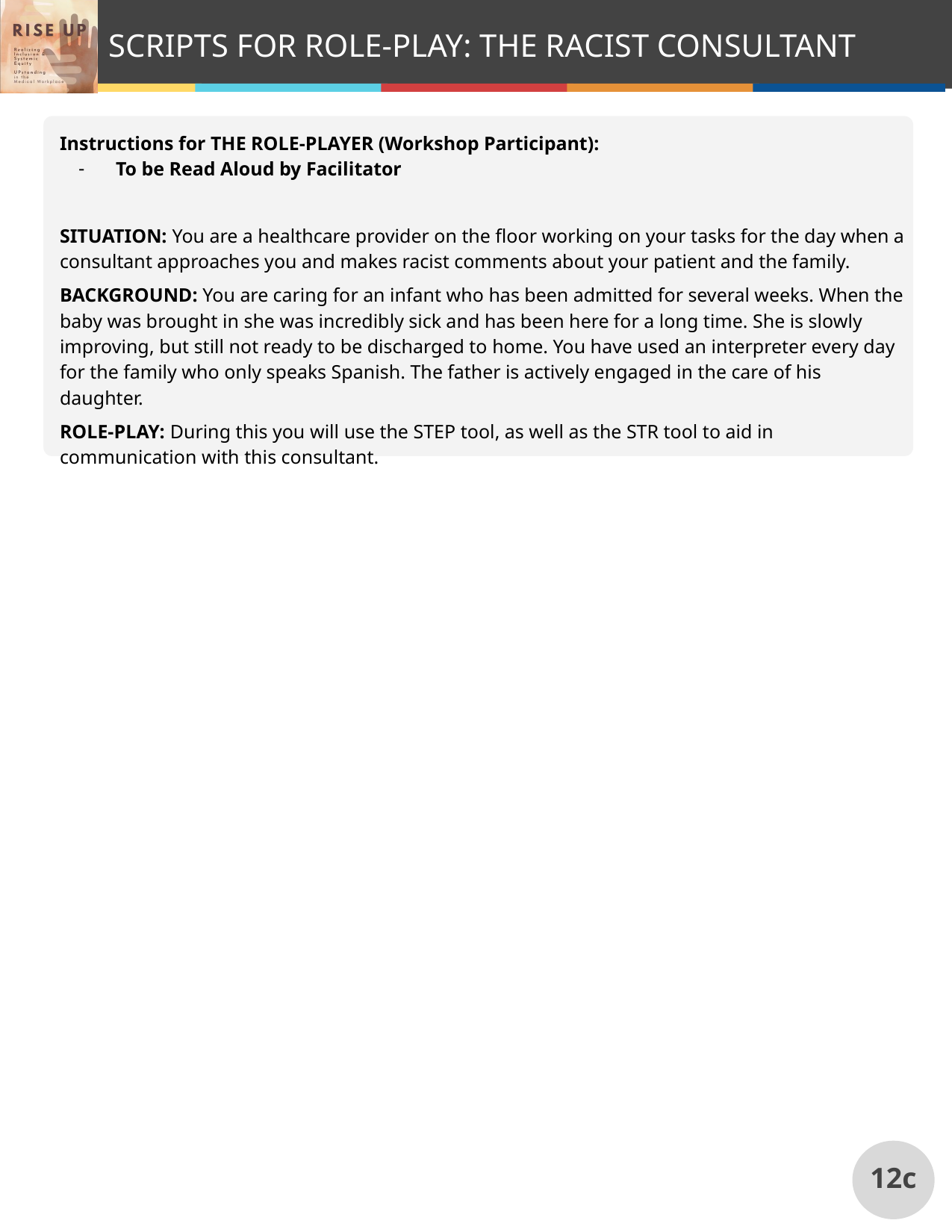

SCRIPTS FOR ROLE-PLAY: THE RACIST CONSULTANT
Instructions for THE ROLE-PLAYER (Workshop Participant):
To be Read Aloud by Facilitator
SITUATION: You are a healthcare provider on the floor working on your tasks for the day when a consultant approaches you and makes racist comments about your patient and the family.
BACKGROUND: You are caring for an infant who has been admitted for several weeks. When the baby was brought in she was incredibly sick and has been here for a long time. She is slowly improving, but still not ready to be discharged to home. You have used an interpreter every day for the family who only speaks Spanish. The father is actively engaged in the care of his daughter.
ROLE-PLAY: During this you will use the STEP tool, as well as the STR tool to aid in communication with this consultant.
12c

## Slide 21
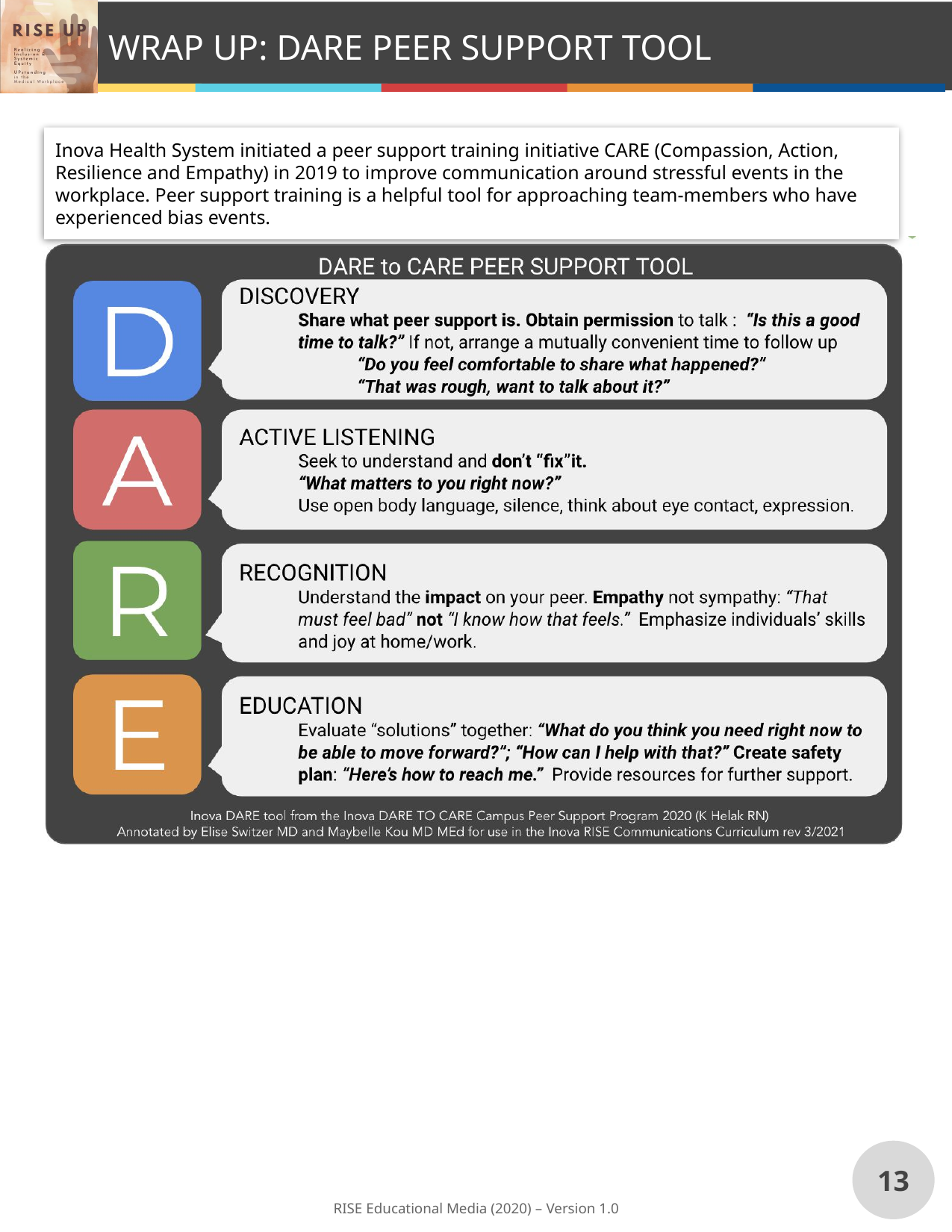

WRAP UP: DARE PEER SUPPORT TOOL
DARE PEER SUPPORT TOOL
Inova Health System initiated a peer support training initiative CARE (Compassion, Action, Resilience and Empathy) in 2019 to improve communication around stressful events in the workplace. Peer support training is a helpful tool for approaching team-members who have experienced bias events.
Inova DARE tool from the Inova DARE TO CARE Campus Peer Support Program 2020:
 Annotated by Elise Switzer MD and Maybelle Kou MD MEd for use in the Inova RISE Communications Curriculum 2020
13
RISE Educational Media (2020) – Version 1.0

## Slide 22
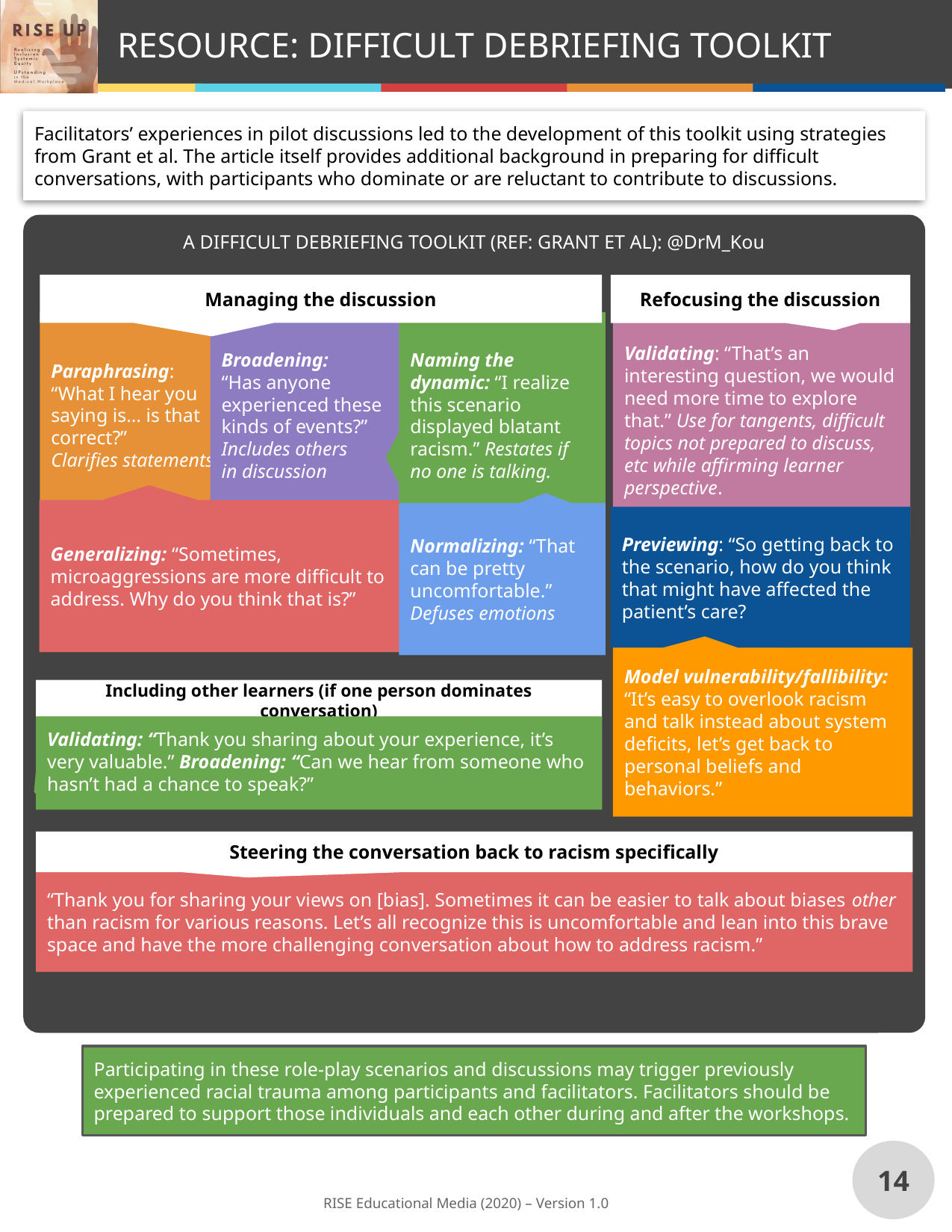

RESOURCE: DIFFICULT DEBRIEFING TOOLKIT
Facilitators’ experiences in pilot discussions led to the development of this toolkit using strategies from Grant et al. The article itself provides additional background in preparing for difficult conversations, with participants who dominate or are reluctant to contribute to discussions.
A DIFFICULT DEBRIEFING TOOLKIT (REF: GRANT ET AL): @DrM_Kou
Refocusing the discussion
Managing the discussion
Validating: “That’s an interesting question, we would need more time to explore that.” Use for tangents, difficult topics not prepared to discuss, etc while affirming learner perspective.
Previewing: “So getting back to the scenario, how do you think that might have affected the patient’s care?
Paraphrasing: “What I hear you saying is… is that correct?”
Clarifies statements
Naming the dynamic: “I realize this scenario displayed blatant racism.” Restates if no one is talking.
Broadening:
“Has anyone experienced these kinds of events?” Includes others
in discussion
Generalizing: “Sometimes, microaggressions are more difficult to address. Why do you think that is?”
Normalizing: “That can be pretty uncomfortable.” Defuses emotions
Model vulnerability/fallibility: “It’s easy to overlook racism and talk instead about system deficits, let’s get back to personal beliefs and behaviors.”
Including other learners (if one person dominates conversation)
Validating: “Thank you sharing about your experience, it’s very valuable.” Broadening: “Can we hear from someone who hasn’t had a chance to speak?”
Steering the conversation back to racism specifically
“Thank you for sharing your views on [bias]. Sometimes it can be easier to talk about biases other than racism for various reasons. Let’s all recognize this is uncomfortable and lean into this brave space and have the more challenging conversation about how to address racism.”
Grant VJ, Robinson T, Catena H, Eppich W, Cheng A. Difficult debriefing situations: A toolbox for simulation educators. Med Teach. 2018 Jul;40(7):703-712. doi: 10.1080/0142159X.2018.1468558. Epub 2018 May 23. PMID: 29792100.
Participating in these role-play scenarios and discussions may trigger previously experienced racial trauma among participants and facilitators. Facilitators should be prepared to support those individuals and each other during and after the workshops.
14
RISE Educational Media (2020) – Version 1.0

## Slide 23
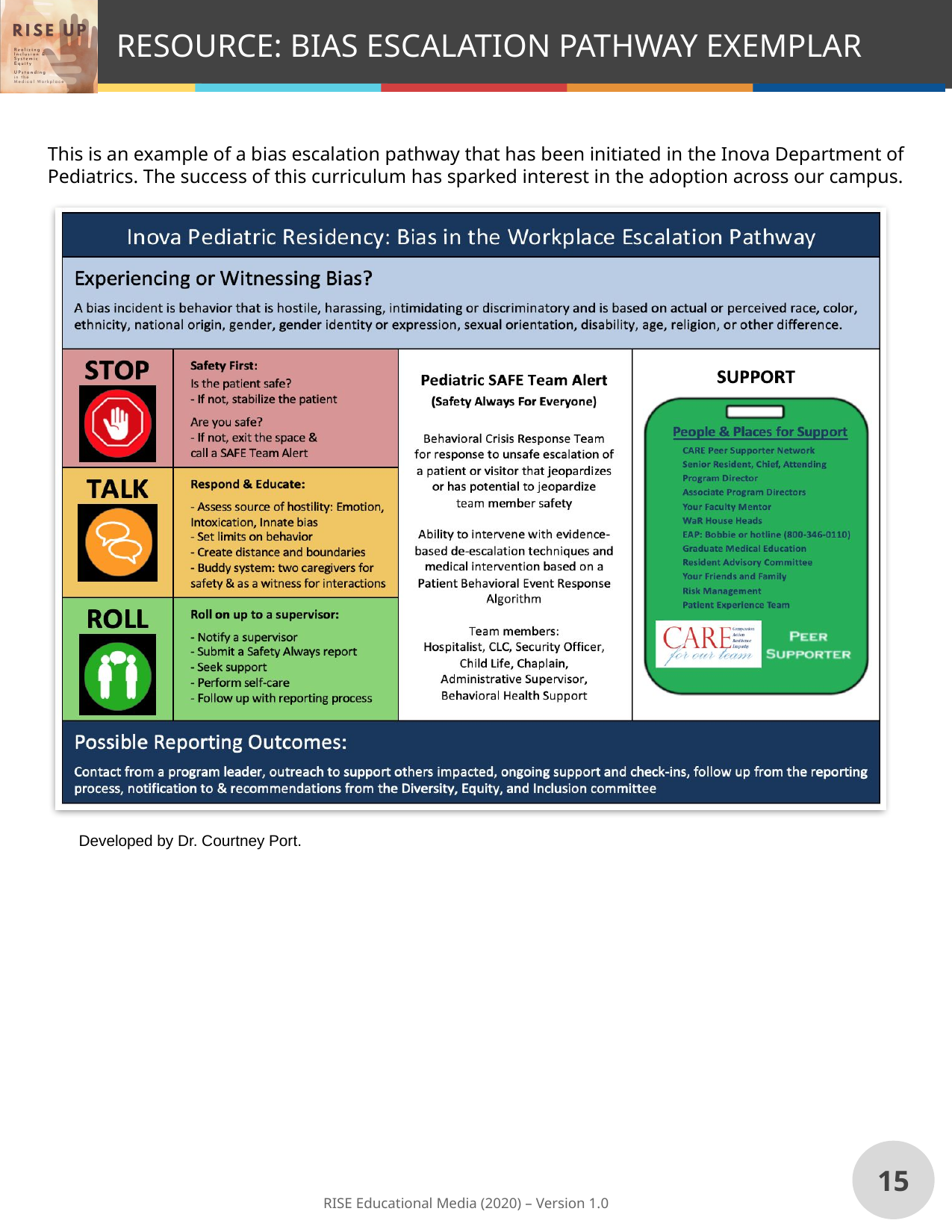

RESOURCE: BIAS ESCALATION PATHWAY EXEMPLAR
This is an example of a bias escalation pathway that has been initiated in the Inova Department of Pediatrics. The success of this curriculum has sparked interest in the adoption across our campus.
Developed by Dr. Courtney Port.
15
RISE Educational Media (2020) – Version 1.0

## Slide 24
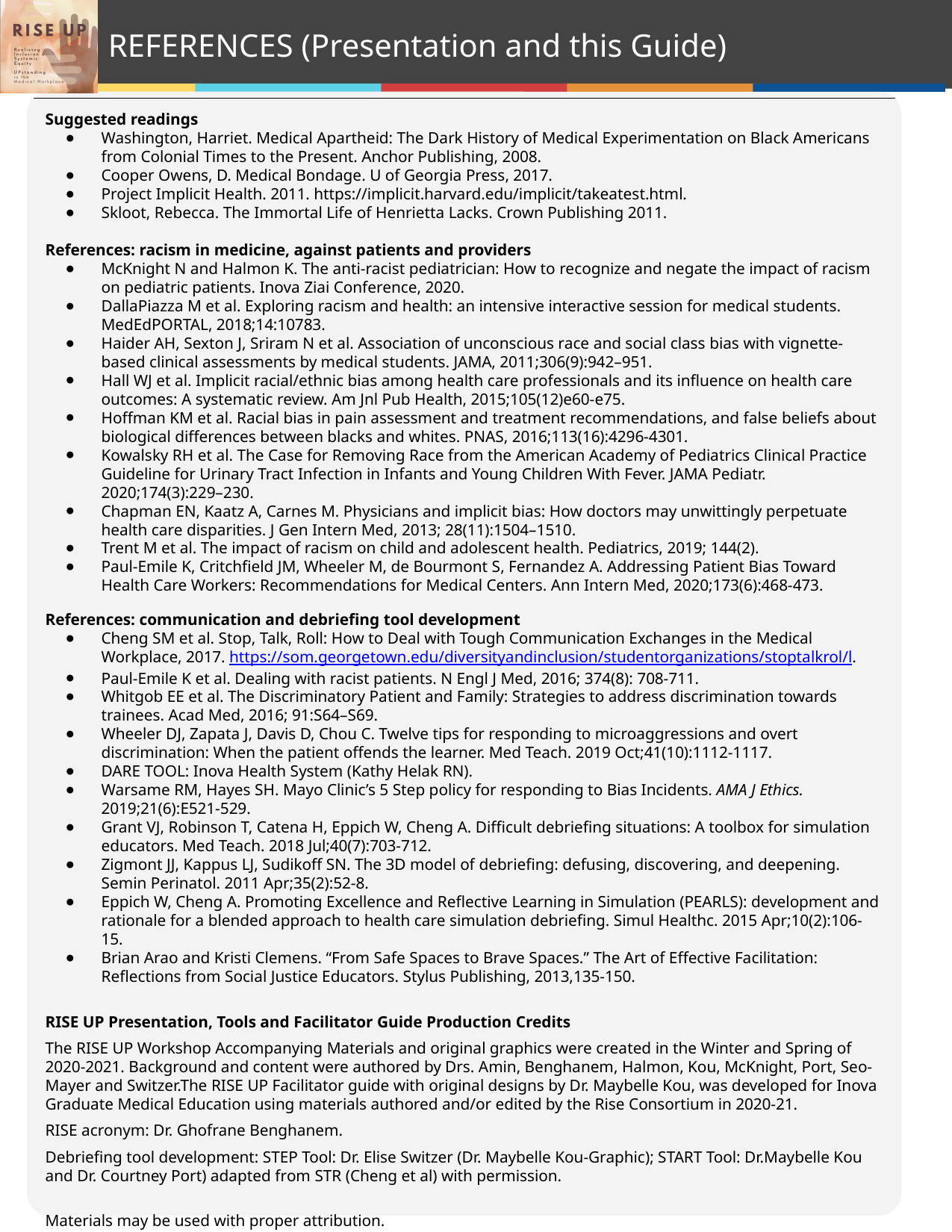

REFERENCES (Presentation and this Guide)
Suggested readings
Washington, Harriet. Medical Apartheid: The Dark History of Medical Experimentation on Black Americans from Colonial Times to the Present. Anchor Publishing, 2008.
Cooper Owens, D. Medical Bondage. U of Georgia Press, 2017.
Project Implicit Health. 2011. https://implicit.harvard.edu/implicit/takeatest.html.
Skloot, Rebecca. The Immortal Life of Henrietta Lacks. Crown Publishing 2011.
References: racism in medicine, against patients and providers
McKnight N and Halmon K. The anti-racist pediatrician: How to recognize and negate the impact of racism on pediatric patients. Inova Ziai Conference, 2020.
DallaPiazza M et al. Exploring racism and health: an intensive interactive session for medical students. MedEdPORTAL, 2018;14:10783.
Haider AH, Sexton J, Sriram N et al. Association of unconscious race and social class bias with vignette-based clinical assessments by medical students. JAMA, 2011;306(9):942–951.
Hall WJ et al. Implicit racial/ethnic bias among health care professionals and its influence on health care outcomes: A systematic review. Am Jnl Pub Health, 2015;105(12)e60-e75.
Hoffman KM et al. Racial bias in pain assessment and treatment recommendations, and false beliefs about biological differences between blacks and whites. PNAS, 2016;113(16):4296-4301.
Kowalsky RH et al. The Case for Removing Race from the American Academy of Pediatrics Clinical Practice Guideline for Urinary Tract Infection in Infants and Young Children With Fever. JAMA Pediatr. 2020;174(3):229–230.
Chapman EN, Kaatz A, Carnes M. Physicians and implicit bias: How doctors may unwittingly perpetuate health care disparities. J Gen Intern Med, 2013; 28(11):1504–1510.
Trent M et al. The impact of racism on child and adolescent health. Pediatrics, 2019; 144(2).
Paul-Emile K, Critchfield JM, Wheeler M, de Bourmont S, Fernandez A. Addressing Patient Bias Toward Health Care Workers: Recommendations for Medical Centers. Ann Intern Med, 2020;173(6):468-473.
References: communication and debriefing tool development
Cheng SM et al. Stop, Talk, Roll: How to Deal with Tough Communication Exchanges in the Medical Workplace, 2017. https://som.georgetown.edu/diversityandinclusion/studentorganizations/stoptalkrol/l.
Paul-Emile K et al. Dealing with racist patients. N Engl J Med, 2016; 374(8): 708-711.
Whitgob EE et al. The Discriminatory Patient and Family: Strategies to address discrimination towards trainees. Acad Med, 2016; 91:S64–S69.
Wheeler DJ, Zapata J, Davis D, Chou C. Twelve tips for responding to microaggressions and overt discrimination: When the patient offends the learner. Med Teach. 2019 Oct;41(10):1112-1117.
DARE TOOL: Inova Health System (Kathy Helak RN).
Warsame RM, Hayes SH. Mayo Clinic’s 5 Step policy for responding to Bias Incidents. AMA J Ethics. 2019;21(6):E521-529.
Grant VJ, Robinson T, Catena H, Eppich W, Cheng A. Difficult debriefing situations: A toolbox for simulation educators. Med Teach. 2018 Jul;40(7):703-712.
Zigmont JJ, Kappus LJ, Sudikoff SN. The 3D model of debriefing: defusing, discovering, and deepening. Semin Perinatol. 2011 Apr;35(2):52-8.
Eppich W, Cheng A. Promoting Excellence and Reflective Learning in Simulation (PEARLS): development and rationale for a blended approach to health care simulation debriefing. Simul Healthc. 2015 Apr;10(2):106-15.
Brian Arao and Kristi Clemens. “From Safe Spaces to Brave Spaces.” The Art of Effective Facilitation: Reflections from Social Justice Educators. Stylus Publishing, 2013,135-150.
RISE UP Presentation, Tools and Facilitator Guide Production Credits
The RISE UP Workshop Accompanying Materials and original graphics were created in the Winter and Spring of 2020-2021. Background and content were authored by Drs. Amin, Benghanem, Halmon, Kou, McKnight, Port, Seo-Mayer and Switzer.The RISE UP Facilitator guide with original designs by Dr. Maybelle Kou, was developed for Inova Graduate Medical Education using materials authored and/or edited by the Rise Consortium in 2020-21.
RISE acronym: Dr. Ghofrane Benghanem.
Debriefing tool development: STEP Tool: Dr. Elise Switzer (Dr. Maybelle Kou-Graphic); START Tool: Dr.Maybelle Kou and Dr. Courtney Port) adapted from STR (Cheng et al) with permission.
Materials may be used with proper attribution.
