# Supplementary material for: Realizing Inclusion and Systemic Equity in Medicine: Upstanding in the Medical Workplace (RISE UP)—an Antibias Curriculum
Source: MedEdPORTAL. 2022 Apr 6;18:11233. doi: 10.15766/mep_2374-8265.11233 (PMC8983799; doi:10.15766/mep_2374-8265.11233)
Supplement: Supplementary file 1 — Video 1 - The Racist Patient.mp4Video 2 - The Racist Provider.mp4Video 3 - The Racist Consultant.mp4Workshop Slides.pptxFacilitator Guide.pptxPreworkshop Survey.docxPostworkshop Survey.docxSimulation Video Transcripts.docx [file mep_2374-8265.11233-s001.zip › F. Preworkshop Survey.docx]

**RISE UP Pre-Workshop Survey for Participants**

Realizing Inclusion and Systemic Equity: Learner pre-workshop survey: please spend 5 minutes to fill out this survey.

* Required

1. **Please enter your favorite fruit and the last 4 # of your cell phone number. For example, Kiwi-7890. This will be used to track changes over time throughout this workshop series without identifying individuals. ***
2. **Have you personally ever witnessed bias in the medical workplace? *** *Mark only one.*
   Yes / No / Unsure
3. **Have you personally ever experienced bias in the medical workplace? *** *Mark only one.*
   Yes / No / Unsure
4. **If you answered yes to either of the previous 2 questions, please select the role of the person(s) who demonstrated biased behavior.** * *Check all that apply.*

N/A, I answered No to both of the previous two questions

Patient
Family member of the patient
Nurse
Resident
Faculty
Medical Student
Other Medical Professional (RT, Med Tech, EMT, etc)
Other:

1. **Where do you seek support on campus when you witness or experience discriminatory behavior? ***
2. **How comfortable do you feel discussing bias as it relates to the following topics with colleagues? *** *Mark only one per topic.*

**Race or Ethnicity:** Very comfortable / Comfortable / Neither comfortable nor uncomfortable / Uncomfortable / Very uncomfortable

**Gender, gender identity or gender expression:** Very comfortable / Comfortable / Neither comfortable nor uncomfortable / Uncomfortable / Very uncomfortable

**Sexual orientation:** Very comfortable / Comfortable / Neither comfortable nor uncomfortable / Uncomfortable / Very uncomfortable

**Spirituality and faith:** Very comfortable / Comfortable / Neither comfortable nor uncomfortable / Uncomfortable / Very uncomfortable

1. **Please state your level of agreement with the following statements: *** *Mark only one per topic.*

**I have the tools to respond to discriminatory behavior in the workplace.** Strongly Agree / Agree / Neither agree nor disagree / Disagree / Strongly Disagree

**I know how to escalate witnessed or experienced discriminatory behavior in the workplace.** Strongly Agree / Agree / Neither agree nor disagree / Disagree / Strongly Disagree

1. **In the past two years have you completed formal training related to the following topics (workshops)? *** *Check all that apply.* implicit bias / cultural competency / difficult conversations / delivering feedback / peer support
2. **Please select your role. If multiple roles, please select the one that best fits with your reason for attending this meeting.** * *Mark only one.*

Faculty

Chief Resident

Resident: PGY1

Resident: PGY2

Resident: PGY3

Resident: PGY-4+

Medical Student

Prefer Not to Answer

Other:
