# Supplementary material for: Realizing Inclusion and Systemic Equity in Medicine: Upstanding in the Medical Workplace (RISE UP)—an Antibias Curriculum
Source: MedEdPORTAL. 2022 Apr 6;18:11233. doi: 10.15766/mep_2374-8265.11233 (PMC8983799; doi:10.15766/mep_2374-8265.11233)
Supplement: Supplementary file 1 — Video 1 - The Racist Patient.mp4Video 2 - The Racist Provider.mp4Video 3 - The Racist Consultant.mp4Workshop Slides.pptxFacilitator Guide.pptxPreworkshop Survey.docxPostworkshop Survey.docxSimulation Video Transcripts.docx [file mep_2374-8265.11233-s001.zip › G. Postworkshop Survey.docx]

**RISE UP Post-Workshop Survey for Participants**

Realizing Inclusion and Systemic Equity: Learner post-workshop survey: please spend 5 minutes to fill out this survey.

* Required

1. **Please enter your favorite fruit and the last 4 # of your cell phone number. For example, Kiwi-7890. This will be used to track changes over time without identifying individuals. ***
2. **After attending this workshop, how comfortable do you feel discussing bias as it relates to the following topics with colleagues? *** Mark only one per topic.

**Race or Ethnicity:** Very comfortable / Comfortable / Neither comfortable nor uncomfortable / Uncomfortable / Very uncomfortable

**Gender, gender identity or gender expression:** Very comfortable / Comfortable / Neither comfortable nor uncomfortable / Uncomfortable / Very uncomfortable

**Sexual orientation:** Very comfortable / Comfortable / Neither comfortable nor uncomfortable / Uncomfortable / Very uncomfortable

**Spirituality and faith:** Very comfortable / Comfortable / Neither comfortable nor uncomfortable / Uncomfortable / Very uncomfortable

3. **Think about the STR Tool (Stop, Talk, and Roll) for responding to experienced or witnessed bias when rating your agreement with the following statements.** * *Mark only one per statement.*

**The STR Tool is easy to learn.** Strongly Agree / Agree / Neither agree nor disagree / Disagree / Strongly Disagree

**The STR Tool is easy to remember.** Strongly Agree / Agree / Neither agree nor disagree / Disagree / Strongly Disagree

**The STR Tool is an effective method for responding to bias in the medical workplace.** Strongly Agree / Agree / Neither agree nor disagree / Disagree / Strongly Disagree

**I am satisfied with the STR Tool.** Strongly Agree / Agree / Neither agree nor disagree / Disagree / Strongly Disagree

**I will likely use the STR Tool if responding to bias in the future.** Strongly Agree / Agree / Neither agree nor disagree / Disagree / Strongly Disagree

4. **Think about the STEP Tool (Step back, Think through biases, Evaluate emotions, Prevent patient impact) for addressing personal biases when rating your agreement with the following statements.** * *Mark only one per statement.*

**The STEP Tool is easy to learn.** Strongly Agree / Agree / Neither agree nor disagree / Disagree / Strongly Disagree

**The STEP Tool is easy to remember.** Strongly Agree / Agree / Neither agree nor disagree / Disagree / Strongly Disagree

**The STEP Tool is an effective method for responding to bias in the medical workplace.** Strongly Agree / Agree / Neither agree nor disagree / Disagree / Strongly Disagree

**I am satisfied with the STEP Tool.** Strongly Agree / Agree / Neither agree nor disagree / Disagree / Strongly Disagree

**I will likely use the STEP Tool if addressing my personal biases in the future.** Strongly Agree / Agree / Neither agree nor disagree / Disagree / Strongly Disagree

5. **Think about the DARE Tool (Discover, Actively listen, Recognize, Educate) for providing peer support when rating your agreement with the following statements.** * *Mark only one per statement.*

**The DARE Tool is easy to learn.** Strongly Agree / Agree / Neither agree nor disagree / Disagree / Strongly Disagree

**The DARE Tool is easy to remember.** Strongly Agree / Agree / Neither agree nor disagree / Disagree / Strongly Disagree

**The DARE Tool is an effective method for responding to bias in the medical workplace.** Strongly Agree / Agree / Neither agree nor disagree / Disagree / Strongly Disagree

**I am satisfied with the DARE Tool.** Strongly Agree / Agree / Neither agree nor disagree / Disagree / Strongly Disagree

**I will likely use the DARE Tool if providing peer support in the future.** Strongly Agree / Agree / Neither agree nor disagree / Disagree / Strongly Disagree

6. **Think about the Example Scripts (What to say to when responding to racism) used during small group discussion and role-play.** * *Mark only one per statement.*

**The Scripts were helpful examples of what to say when encountering bias.** Strongly Agree / Agree / Neither agree nor disagree / Disagree / Strongly Disagree

**I will likely use one of these Scripts if responding to bias in the future.** Strongly Agree / Agree / Neither agree nor disagree / Disagree / Strongly Disagree

**Role-playing to practice these Scripts was beneficial.** Strongly Agree / Agree / Neither agree nor disagree / Disagree / Strongly Disagree

7. **Please state your level of agreement with the following statements:** * *Mark only one per statement.*

**I have the tools to respond to discriminatory behavior in the workplace.** Strongly Agree / Agree / Neither agree nor disagree / Disagree / Strongly Disagree

**I know how to escalate witnessed or experienced discriminatory behavior in the workplace.** Strongly Agree / Agree / Neither agree nor disagree / Disagree / Strongly Disagree

8. **Please state your level of agreement with the following statements.** * *Mark only one oval per statement.*

**The information covered in this workshop was useful to my professional work.** Strongly Agree / Agree / Neither agree nor disagree / Disagree / Strongly Disagree

**This workshop prepared me to recognize and respond to bias in the medical workplace.** Strongly Agree / Agree / Neither agree nor disagree / Disagree / Strongly Disagree

9. **I would recommend this workshop to a colleague.** * *Mark only one.*

Yes / No / Unsure

10. **What will you incorporate from this workshop into your practice?** *

11. **Which part of this workshop most resonated with you? How so?** *

12. **What would you like to hear more about on this topic?** *

13. **Please give suggestions to improve this workshop.** *
