# Supplementary material for: Realizing Inclusion and Systemic Equity in Medicine: Upstanding in the Medical Workplace (RISE UP)—an Antibias Curriculum
Source: MedEdPORTAL. 2022 Apr 6;18:11233. doi: 10.15766/mep_2374-8265.11233 (PMC8983799; doi:10.15766/mep_2374-8265.11233)
Supplement: Supplementary file 1 — Video 1 - The Racist Patient.mp4Video 2 - The Racist Provider.mp4Video 3 - The Racist Consultant.mp4Workshop Slides.pptxFacilitator Guide.pptxPreworkshop Survey.docxPostworkshop Survey.docxSimulation Video Transcripts.docx [file mep_2374-8265.11233-s001.zip › H. Simulation Video Transcripts.docx]

**Video 1: The Racist Patient**

*Dr. Harris, Pediatric Intern, knocks and enters the room.*

Dr. Harris: Hello, I’m Dr. Harris. I’m one of the hospital interns. Nice to meet you guys. I know you had a long night down in the Emergency Room. We’ll do our best to get you tucked in as soon as we can. I spoke with the Emergency Room doctors about your daughter and it sounds like she’s been very sick lately so I’m here to hear a little bit more about the story and what’s going on with her.

Racist Parent: I’m not talking to you. I don’t want a Black person taking care of my daughter. Can you - can you get out of my room?

Dr. Harris: Uh…. umm…. Let me go get my Senior Resident. Excuse me.

*Dr. Harris exits the room.*

*Dr. Rodriguez, Senior Resident, knocks and enters the room.*

Dr. Rodriguez: My name is Dr. Rodriguez. I’m the Senior in charge of your daughter’s care. I understand you did not walk to speak to my Intern, but we have some things to discuss to care for her.

Racist Parent: I do not want someone of YOUR KIND taking care of my daughter. You don’t even speak English. Just get out.

Dr. Rodriguez: I’m - I’m sorry. I’ll get my Attending.

Racist Parent: Just go.

*Dr. Rodriguez exits the room.*

*Dr. Ahmad, Hospitalist Attending, enters the room.*

Dr. Ahmad: Hello! Hi, I’m Dr. Ahmad. I’m one of the Attendings here.

Racist Parent: Oh my god… what is wrong with you people? I don’t want an incompetent foreigner taking care of my daughter.

Dr. Ahmad: We’re all here to help her, but only if you allow us to…

Racist Parent: No. This isn’t going to work. I need you to leave.

Dr. Ahmad: In this situation, then I have to ask my colleague in the morning to assess her...

Racist Patient: Whatever you need to do. Just go!

Dr. Ahmad: Okay….

*Dr. Ahmad exits the room.*

*The next morning, Dr. Smith enters the room.*

Dr. Smith: Hi, I’m Dr. Smith. I’m the pediatrician who’s going to be taking care of your daughter.

Racist Parent: FINALLY! A normal doctor.

Dr. Smith: Okay. I understand there were some issues overnight with the team, but…

Racist Parent: Yeah, it was like being in a third world country all night. I’m just glad we finally have a white doctor. So - YOU need to be taking care of my daughter now.

Dr. Smith: Okay…

**Video 2: The Racist Provider:**

Resident 1: Okay… so 17 year old Black female, Sickle Cell Type SS, being admitted for sickle cell pain crisis, third in past 6 months, requiring IV morphine x 2 in ED. Followed by hematology here at Inova. Anything else? Okay, thank you!

Resident 2: Hey, who was that?

Resident 1: Oh my god, you know who. She was here just two months ago. It’s like she has nothing better to do than to just be admitted and waste our time.

Resident 2: I feel like she just gets so bored with home that she just decides to come in for entertainment or something.

Resident 1: Remember how long she was here last time?

Resident 2: Oh my god - it was forever!

Resident 1: She’s - you know she’s got to be addicted to pain pills.

Resident 2: She was on dilaudid PCA FOREVER last time.

Resident 1: I know, I know! You know - seriously, even our onc kids, who have, like, a REASON to be here, they’re not on as many pain pills as she is!

Resident 2: I know! I know! I just don’t understand why she can’t just take her hydroxyurea like everyone else and stay at home instead of wasting our time.

Resident 1: Oh my god. Okay, let’s get going.

**Video 3: The Racist Consultant:**

Dr. Bradford: Hi Mr. Cruz!

Mr. Cruz: Hola, mucho gusto. ¿Cómo estás? (Hi nice to meet you. How are you?)

Dr. B: (speaking very loudly and slowly) Good, good. Um… so, it looks like everything is the same as before. No changes. We’re just waiting for her to…

Mr. Cruz: Uh - no entiendo. No hablo inglés, yo (Uh - I don't understand. I don't speak English)

Dr. B: Okay… umm… BUENO. She’s fine. Bueno.

Mr. Cruz: Pero, ¿qué es bueno? (But, what is good?)

Dr. C: Good. She’s fine. She’s fine. And no changes. Okay?

Mr. Cruz: Tal vez puede usar el ipad como otros doctores… (Maybe you can use the ipad like the other doctors ...)

Dr. C: No, no. She’s - she’s okay. She’s fine. No changes. Questions? Eh, preguntas?

Mr. Cruz: Si, tengo preguntas! (Yes, I have questions!)

Dr. B: Ask the primary team, okay?

Mr Cruz: ¿A quien? (Who?)

Dr. C: The PRIMARY TEAM. They’ll answer preguntas.

Mr. Cruz: No entiendo… (I don’t understand...)

Dr. C: PRIMARY TEAM. Preguntas. Okay? She’s fine. Bueno!

Mr. Cruz: (Gives up) Okay.

Dr. C: Okay! Gracias.

*Dr. C exits the room and walks to the workstation where two residents are sitting.*

Dr. C: (Sigh)

Resident 1: Hey, Dr. Bradford.

Dr. Bradford: Can you guys believe this? We are still taking care of that girl after all of this time. She has been here for MONTHS, she will never have to pay a dime. We as taxpayers are the ones that are going to pay for it. It is such a waste of resources. She should just go back to her own country where they can take care of her there.

*Residents 1 and 2 exchange looks but say nothing.*
